# Growth of non‐English‐language literature on biodiversity conservation

**DOI:** 10.1111/cobi.13883

**Published:** 2022-03-24

**Authors:** Shawan Chowdhury, Kristofer Gonzalez, M. Çisel Kemahlı Aytekin, Seung‐Yun Baek, Michał Bełcik, Sandro Bertolino, Sjoerd Duijns, Yuqing Han, Kerstin Jantke, Ryosuke Katayose, Mu‐Ming Lin, Elham Nourani, Danielle Leal Ramos, Marie‐Morgane Rouyer, William Sidemo‐Holm, Svetlana Vozykova, Veronica Zamora‐Gutierrez, Tatsuya Amano

**Affiliations:** ^1^ School of Biological Sciences University of Queensland Brisbane Queensland Australia; ^2^ Centre for Biodiversity and Conservation Science, School of Biological Sciences University of Queensland Brisbane Queensland Australia; ^3^ Environmental Science and Resource Management California State University Channel Islands Camarillo California USA; ^4^ Department of Molecular Biology and Genetics Koç University Istanbul Turkey; ^5^ Graduate School of Agricultural Science Tokyo University of Agriculture and Technology Fuchu Japan; ^6^ Institute of Nature Conservation, Polish Academy of Sciences Kraków Poland; ^7^ Department of Life Sciences and Systems Biology University of Turin Torino Italy; ^8^ Sovon Dutch Centre for Field Ornithology Nijmegen The Netherlands; ^9^ State Key Laboratory of Biocontrol, Department of Ecology/School of Life Sciences Sun Yat‐sen University Guangzhou China; ^10^ Center for Earth System Research and Sustainability University of Hamburg Hamburg Germany; ^11^ School of Environmental Science and Engineering Southern University of Science and Technology Shenzhen China; ^12^ Department of Migration Max Planck Institute of Animal Behavior Radolfzell Germany; ^13^ Department of Biology University of Konstanz Konstanz Germany; ^14^ Plant Technology and Environmental Monitoring Ltd Technological Park of São José dos Campos São José dos Campos Brazil; ^15^ CEFE, Univ Montpellier, CNRS, EPHE, IRD Montpellier France; ^16^ Centre for Environmental and Climate Science Lund University Lund Sweden; ^17^ Faculty of Energy and Ecotechnology (GreenTech) ITMO University St Petersburg Russia; ^18^ CONACYT ‐ Centro Interdisciplinario de Investigación para el Desarrollo Integral Regional Unidad Durango (CIIDIR) Instituto Politécnico Nacional Ciudad de México México

**Keywords:** biodiversity information, evidence synthesis, global biodiversity databases, languages, language barrier, publication bias, 生物多样性信息;证据综合;全球生物多样性数据库;语言;语言障碍;出版偏向, barrera del lenguaje, bases de datos de la biodiversidad mundial, idiomas, información sobre la biodiversidad, sesgo de publicación, síntesis de evidencias

## Abstract

English is widely recognized as the language of science, and English‐language publications (ELPs) are rapidly increasing. It is often assumed that the number of non‐ELPs is decreasing. This assumption contributes to the underuse of non‐ELPs in conservation science, practice, and policy, especially at the international level. However, the number of conservation articles published in different languages is poorly documented. Using local and international search systems, we searched for scientific articles on biodiversity conservation published from 1980 to 2018 in English and 15 non‐English languages. We compared the growth rate in publications across languages. In 12 of the 15 non‐English languages, published conservation articles significantly increased every year over the past 39 years, at a rate similar to English‐language articles. The other three languages showed contrasting results, depending on the search system. Since the 1990s, conservation science articles in most languages increased exponentially. The variation in the number of non‐English‐language articles identified among the search systems differed markedly (e.g., for simplified Chinese, 11,148 articles returned with local search system and 803 with Scopus). Google Scholar and local literature search systems returned the most articles for 11 and 4 non‐English languages, respectively. However, the proportion of peer‐reviewed conservation articles published in non‐English languages was highest in Scopus, followed by Web of Science and local search systems, and lowest in Google Scholar. About 20% of the sampled non‐English‐language articles provided no title or abstract in English; thus, in theory, they were undiscoverable with English keywords. Possible reasons for this include language barriers and the need to disseminate research in countries where English is not widely spoken. Given the known biases in statistical methods and study characteristics between English‐ and non‐English‐language studies, non‐English‐language articles will continue to play an important role in improving the understanding of biodiversity and its conservation.

## Resumen

**Resumen**: El inglés es reconocido como el idioma de la ciencia y las publicaciones en inglés (PI) cada vez son más. Con frecuencia se asume que el número de publicaciones en idiomas diferentes al inglés está disminuyendo. Esta suposición contribuye al uso reducido de las publicaciones que no están en inglés en las ciencias, prácticas y políticas de la conservación, especialmente a nivel internacional. Sin embargo, el número de artículos de conservación publicados en diferentes idiomas está muy mal documentado. Usamos sistemas de búsqueda locales e internacionales para buscar artículos científicos sobre la conservación de la biodiversidad publicados entre 1980 y 2018 en inglés y en quince idiomas diferentes al inglés. También comparamos la tasa de incremento de publicaciones en los diferentes idiomas. En doce de los quince idiomas diferentes al inglés, los artículos de conservación publicados incrementaron significativamente cada año durante los últimos 39 años, una tasa similar a los artículos en inglés. Los otros tres idiomas mostraron resultados contrastantes según el sistema de búsqueda. Desde la década de 1990, los artículos sobre ciencias de la conservación incrementaron exponencialmente en la mayoría de los idiomas. La variación en el número de artículos identificados en idiomas diferentes al inglés difirió notablemente de acuerdo con los sistemas de búsqueda (p. ej.: en el caso del chino simplificado, obtuvimos 11,148 artículos con el sistema de búsqueda local y 803 con Scopus). Google Scholar y los sistemas locales de búsqueda arrojaron la mayor cantidad de artículos en 11 y 4 idiomas diferentes al inglés, respectivamente. Sin embargo, la proporción de artículos sobre conservación revisados por pares y publicados en idiomas diferentes al inglés fue mayor en Scopus, seguida por Web of Science y los sistemas locales de búsqueda, con la menor proporción en Google Scholar. Aproximadamente el 20% de la muestra de artículos en idiomas diferentes al inglés no contaban con título o con resumen en inglés; por lo tanto, en teoría, eran imposibles de encontrar mediante palabras clave en inglés. Las posibles explicaciones de esto incluyen las barreras del idioma y la necesidad de difundir la investigación en países en los que el inglés no se habla extensamente. Con los sesgos conocidos de los métodos estadísticos y de las características de estudio entre los trabajos en inglés y en otros idiomas, los artículos en idiomas diferentes al inglés seguirán desempeñando un papel importante en el entendimiento de la biodiversidad y su conservación.

Incremento de la Literatura sobre la Conservación de la Biodiversidad en Idiomas Diferentes al Inglés

## 摘要

如今, 英语在科学研究中被广泛的使用, 同时英语出版物 (ELPs) 也在迅速增加。非英语科学出版物的数量通常被认为正在减少。这一假设导致非英语出版物在保护科学、实践和政策中的使用不足, 特别是在国际层面。并且, 关于以不同语言发表的保护科学的文献数量的报道也鲜有记载。因此，我们使用本土和国际搜索系统搜索并统计了1980年至2018年间以英语和15种非英语发表的关于生物多样性和保护的文献。此外, 我们还比较了不同语言文献的增长率。我们发现, 在过去39年中, 每年出版的关于保护的非英语文献中, 至少有12种语言的文献数量显著增加, 增长速度与英语文献相似。根据搜索系统的不同, 另外3种语言文献的结果却相反。保护相关的文献从20世纪80年代初开始出现, 自20世纪90年代以来, 大多数语言的保护相关的文献呈指数级增长。我们还观察到, 在搜索系统中发现的非英语文献数量存在显著差异。谷歌学术和本土文献搜索系统中共有11种和4种非英语语言的文献最多。然而, 以非英语语言发表同行评议的相关文献的比例在Scopus中最高, 其次是Web of Science和本土搜索系统, 在谷歌学术中最低。在抽样的非英语文献中, 约有20%的文献没有英文标题或摘要。因此, 理论上 无法使用英语关键词检索。造成这种情况的原因可能是因为语言障碍, 或者在英语不普及的国家是否有必要传播科学研究。鉴于英语和非英语研究在统计结果和研究特征方面存在的已知差异, 非英语文献将继续在提高我们对生物多样性和保护的理解方面发挥重要作用。

增长中的生物多样性保护的非英语文献

## INTRODUCTION

Nearly, 77% of Earth's land and 87% of its ocean have been modified owing to human activities (Ceballos et al., 2015; Chowdhury et al., 2021; Venter et al., 2016). To effectively conserve the remaining biodiversity, making the best use of available scientific knowledge is essential (Sutherland et al., 2004; Tilman et al., 2017; Fraixedas et al., 2022). However, there is a substantial geographical bias in the availability of scientific knowledge on biodiversity conservation, especially for scientific knowledges published in English (Hickisch et al., 2019; Wilson et al., 2016). Such biases seriously hinder the understanding of local biodiversity and the development and implementation of effective conservation actions and policies in regions with limited scientific information, particularly the Global South (e.g., Blicharska et al., 2017; Toomey, 2016; Nuñez et al., 2021).

Despite the current underuse of non‐English‐language scientific knowledge at the global level (Lynch et al., 2021), many articles are published in languages other than English. For example, nearly 36% of conservation articles published in 2014 were in non‐English languages (Amano et al., 2016). Most information on endemic and threatened bat species in Japan is only available in Japanese (Preble et al., 2021). Similarly, scientific articles on China's Belt and Road Initiative, a continental‐scale infrastructure development that has potentially disastrous consequences for biodiversity in the region (Lechner et al., 2018), are often available only in simplified Chinese (Teo et al., 2020).

The majority of research published in non‐English languages often remains unrecognized by scientists and decision makers; consequently, it is underused in global conservation planning and decisions (Fabian et al., 2019). Ignoring the vast amount of scientific knowledge available only in non‐English languages could have serious consequences for conservation science, policies, and practices for the following three reasons. First, non‐English‐language studies provide local knowledge generated by field practitioners, who often find it challenging to publish their work in English if they are non‐native English speakers (Amano et al., 2016). Second, because there can be a systematic difference in statistical results between English‐ and non‐English‐language studies, ignoring non‐English‐language studies can bias the outcomes of evidence syntheses, such as meta‐analyses (Konno et al., 2020). Finally, non‐English‐language studies can provide important scientific knowledge on conservation in areas and for species where little or even no English‐language evidence is available (Amano et al., 2021). The role of non‐English‐language studies could be especially crucial in countries with emerging or developing economies, which are also the areas in which most biodiversity hotspots occur (Cincotta et al., 2000). English is often not widely spoken in these countries, and difficulties in publishing in English are considered key barriers for conservation scientists and practitioners in such countries (e.g., Müller & Opgenoorth, 2014; Nuñez et al., 2019; Valenzuela‐Toro & Viglino, 2021).

Since the end of World War II, English has become the *lingua franca* of science, and even non‐native English speakers have increasingly been publishing their work in English (Fung, 2008; Gordon, 2012; López‐Navarro et al., 2015). It is often assumed that scientific articles are being published less frequently in non‐English languages (e.g., Montgomery, 2013; Baethge, 2013; Di Bitetti & Ferreras, 2017), and this partly contributes to the current underestimation of the importance of non‐English‐language literature in conservation science, policies, and practices (Amano et al., 2021). However, temporal changes in the number of conservation articles published in different languages are not well documented (Amano et al., 2016). We sought to investigate the changes over time in the number of scientific articles on biodiversity conservation published in 16 languages. Most previous investigations on the availability of non‐English‐language conservation studies have been conducted based on literature searches in either Web of Science or Scopus, although most non‐English‐language journals are not indexed in these search systems (Haddaway et al., 2020; Nuñez & Amano, 2021). Amano et al. (2016) estimated the number of conservation‐related scientific documents by language, but only for a single year (2014) and based only on a search in Google Scholar. Although Google Scholar supports literature searches in most major languages and thus effectively identifies non‐English‐language articles, it misses some important literature (Haddaway et al., 2015; Gusenbauer & Haddaway, 2020). A more promising approach for estimating changes in the number of conservation‐related articles in different languages is to search each language's literature in multiple literature search systems, including local‐language‐specific databases (Karlsson et al., 2007). However, no such attempt has been made to date.

To determine the temporal trends in published articles on biodiversity conservation, we compiled the number of articles published annually in English and in 15 non‐English languages by searching both local (for non‐English languages) and international (for English and non‐English languages) literature search systems. We then estimated and compared the rate of change in the number of conservation articles among the languages to test the common assumption that the number of published non‐English‐language articles has decreased, which implies they play a less important role in conservation.

## METHODS

To determine how the number of conservation articles published in different languages has changed over time, we searched multiple literature databases and search platforms and compared the number of published articles per year in each language. We selected the timeframe of 1980−2018 (39 years) because the number of articles on biodiversity and conservation started increasing substantially in the 1980s (e.g., Soulé, 1985). We searched scientific documents for the keywords *biodiversity* AND *conservation* in a set of international and local literature databases and search platforms in 16 languages (English, Spanish, Portuguese, simplified Chinese, traditional Chinese, French, Italian, German, Japanese, Korean, Swedish, Polish, Turkish, Russian, Persian, and Dutch). These 16 languages are the national languages of the 20 countries that produced the most scientific and technical journal articles in 2009 (Amano et al., 2016). The international search systems, we used were Google Scholar, Web of Science, and Scopus.

### Searches in Web of Science

When searching for published articles by year in Web of Science, we used the following databases: Web of Science Core Collection, Current Contents Connect, Data Citation Index, Derwent Innovations Index, KCI‐Korean Journal Database, MEDLINE, Russian Science Citation Index, and SciELO Citation Index. We limited the year accordingly (1980–2018), searched with TS = (*biodiversity* AND *conservation*) (i.e., using English keywords), and recorded the number of articles in each year for each language using the information provided in the search results about the articles’ languages. The Web of Science search was conducted on 4 December 2020.

### Searches in Google Scholar

For Google Scholar, we used the translation of the same two English keywords in 15 non‐English languages (translated keywords in Appendix S1). We included at least one native speaker of each language in the language search. All had a bachelor's degree, but often had higher research (i.e., master's or doctorate) degrees, in ecology or conservation (Table 1). Searches were restricted to websites written in each language (apart from Swedish, Russian, and Persian because these options were unavailable [Amano et al., 2016]). We limited the year accordingly (e.g., 1980−1980, 1981−1981, … 2018−2018) and recorded the number of articles in each year for each language. The search on Google Scholar was conducted from 5 November 2019 to 13 November 2019 in Brisbane, Australia. Although search results on Google Scholar may vary depending on the location of searches, we assumed that this issue would not affect our estimates of temporal trends in the number of articles published in each language, given that all searches were conducted in the same location.

**TABLE 1:** Local literature search systems covering non‐English‐language literature used in searches to identify relevant studies

Language	Name of native speakers in charge of the assessment	Database	URL	Search date
Spanish	Veronica Zamora‐Gutierrez	SciELO	https://scielo.org/en	7−14 May 2020
Portuguese	Danielle Leal Ramos	SciELO	https://scielo.org/en	7−14 May 2020
Chinese (simplified)	Yuqing Han	CNKI	https://cnki.net/	7−14 May 2020
Chinese (traditional)	Mu‐Ming Lin	Airiti Library	https://www.airitilibrary.com/	7−14 May 2020
French	Marie‐Morgane Rouyer	Persee	https://www.persee.fr/	6 February 2021
German	Kerstin Jantke	BASE	https://de.base‐search.net/	27 January 2021
Japanese	Ryosuke Katayose	J‐Stage	https://www.jstage.jst.go.jp/browse/‐char/en	7−14 May 2020
Korean	Seung‐Yun Baek	Korean Citation Index	https://www.kci.go.kr/kciportal/main.kci?locale=en	7 September 2020
Polish	Michał Bełcik	Polska Bibliografia Naukowa	https://pbn.nauka.gov.pl/core/#/home	12 October 2020
Turkish	M. Çisel Kemahlı Aytekin	DergiPark	https://dergipark.org.tr/en/	18 September 2020
Russian	Svetlana Vozykova	Elibrary	https://elibrary.ru/defaultx.asp?	15 September 2020
Persian	Elham Nourani	SID	https://www.sid.ir/	7 September 2020
Dutch	Sjoerd Duijns	Narcis	https://www.narcis.nl/	7−14 May 2020

### Searches in Scopus

Similarly, for Scopus, we limited the year accordingly, searched with TITLE‐ABS‐KEY (biodiversity and conservation) (i.e., with English keywords), and recorded the number of articles per year for each language based on the information provided in the search results. The search on Scopus was conducted from 14 November 2019 to 23 November 2019. Neither Web of Science nor Scopus distinguish between simplified and traditional Chinese, so we used search results for Chinese in general for these two search systems.

### Searches in local literature search systems

Because international literature search systems are unlikely to fully include non‐English‐language articles, we also identified the most comprehensive and relevant literature search system in each of the 15 non‐English languages, based on discussions with the native speakers of each language involved in this study. In cases where we found more than one non‐English‐language search system for a language, we adopted the most comprehensive search system based on discussions with each native speaker. We identified 12 local literature search systems covering 13 non‐English languages, but we could not find any for the other two languages: Swedish and Italian (Table 1). In each literature search system, we searched with the translation of the same two keywords, *biodiversity* AND *conservation*, in each language (translated keywords in Appendix S1), selected the year range (or manually collated the number of articles by year for Turkish), and recorded the total number of articles published each year (see Table 1 for the date of each search). Our preliminary searches in Polish and German identified many irrelevant articles; thus, the translation of *biodiversity* was used only for Polish and German.

### Assessment of relevance, quality, and visibility of non‐English‐language articles

Because we used only two keywords, search results may include articles not relevant to biodiversity conservation. Further, the quality and visibility of the articles identified may also differ among literature search systems and languages. Therefore, we also investigated the relevance (i.e., whether each article was actually about biodiversity conservation), quality (whether each article was peer reviewed), and visibility (whether each article provided the title and abstract in English) of the non‐English‐language articles identified in each literature search system in each language. The assessment was conducted by native speakers of each language who were involved in this study.

The assessment was based on a subset of articles sampled from the 2018 search results for each literature search system in each language. Using the ‘sample.size.prop’ function in R package samplingbook (Manitz et al., 2020), we first determined the sample size for each literature search system in each language (assuming the expected proportion was *p* = 0.5, a finite small population correction of the total number of articles identified in 2018 on the system in the language, precision [*e*] of 0.1, and confidence level of 0.95).

We then determined the number of articles to sample from each search result as follows. If the sample size estimated from the R function was ≥50, the estimated sample size was the number of articles to sample from the search results of the search system. If the estimated sample size was <50, 50 articles from the search result, or all articles if there were fewer than 50 articles were sampled.

Next, we extracted the determined number of articles from the search result for 2018 at regular intervals. For example, if the search identified 1000 articles and the number of articles to sample was 88, we sampled the first article and one in every 11 articles (i.e., 1000/88 = 11) from the search result. Google Scholar shows only the first 1000 articles identified. Therefore, if the total number of articles identified was >1000, we divided 1000 by the number of articles to sample to identify the interval of our samples. For example, if the total number of articles identified on Google Scholar was 20,000 and the number of articles to sample was 96, we sampled the first paper and one in every 10 papers (1000/96 = 10) from the search results.

For each of the articles sampled as described above, we read at least the title and abstract of the article, and the main text if needed, and recorded the following information: whether the article was actually about biodiversity conservation (i.e., articles that mentioned biodiversity or its conservation in any part of the article); the language of the main text; whether an English title was provided; whether an English abstract was provided; article type (peer‐reviewed journal articles, books, theses, or other); and the URL (web link).

### Analyses

To estimate the rate of change in the number of articles on biodiversity conservation, we fitted generalized linear models with a negative binomial distribution with the glmmTMB package (Brooks et al., 2017) in R 4.0.4 (R Core Team, 2021). We used the number of articles published in each language per year as the response variable and the published year (centered) as the explanatory variable. Because some languages seemed to show a nonlinear pattern in changes in the number of articles over time, we compared models with and without the quadratic year term based on Akaike information criterion. To deal with the zero‐inflated nature of the data, we removed data from the years prior to the first year in which each language had at least one published study, and we included only languages with at least 10 years of published studies in the analyses. The estimated coefficient of the year term from the model without the quadratic term was used as the rate of change in the number of conservation articles. We used the ggplot2 (Wickham, 2011) package for visualization. Due to the small sample size, we could not estimate the coefficients for quadratic models in some language‐database combinations. For this, we discuss only the linear models in the main text and provide output of the quadratic model in Appendix S3.

## RESULTS

**FIGURE 1:**
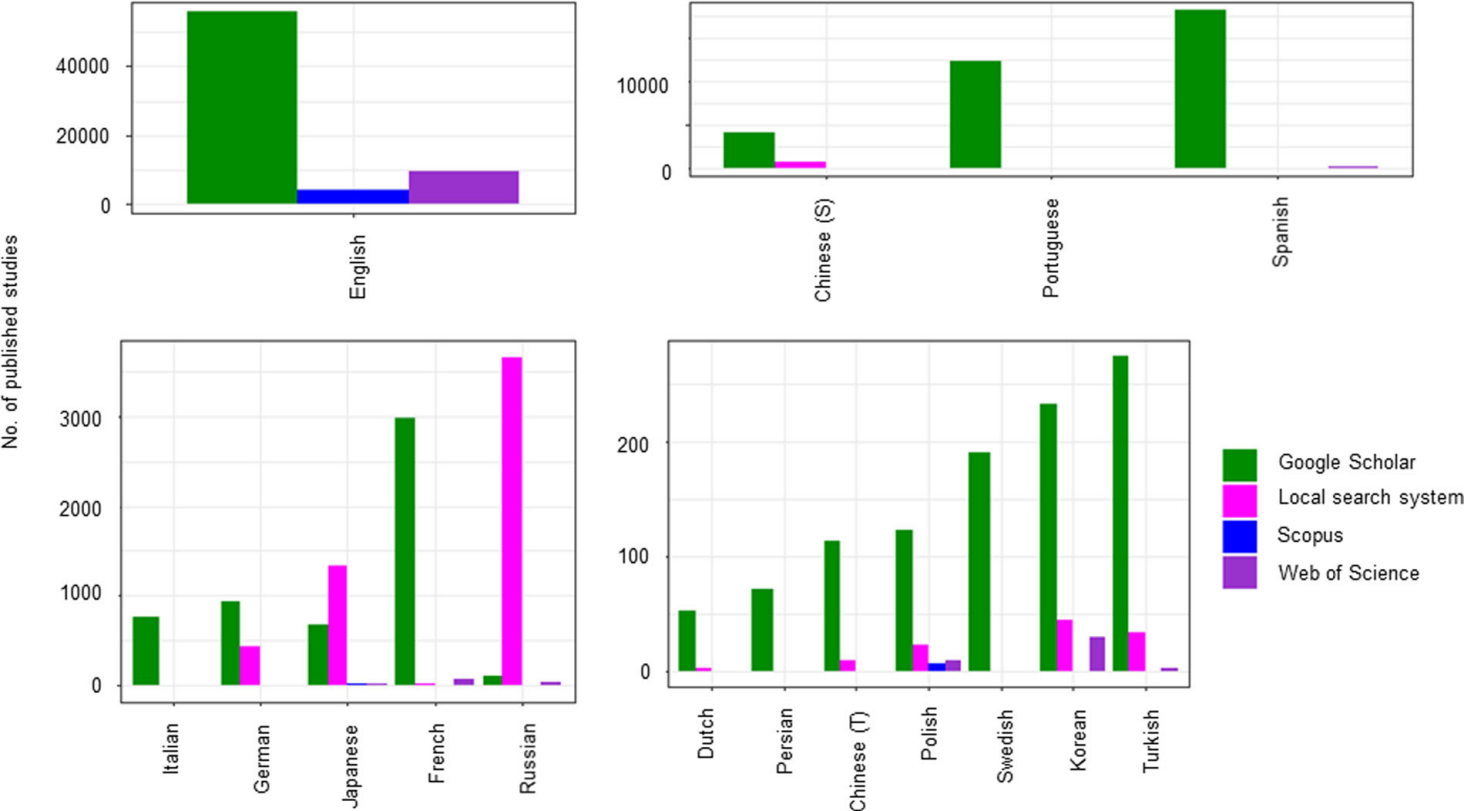
Number of biodiversity conservation articles published in 2018 in 16 languages based on searches for the keywords *biodiversity* AND *conservation* (translated into each language) (translated keywords in Appendix S1) in three international (Google Scholar, Scopus, and Web of Science) and 11 local (see Table 1) search systems (S, simplified; T, traditional). Because neither Scopus nor Web of Science distinguish between simplified and traditional Chinese, search results for Chinese in general from the two search systems are shown under simplified Chinese

Searches on Google Scholar identified the largest number of non‐English‐language articles on biodiversity conservation across the 16 languages (a total of 97,014 articles), whereas those on Web of Science (10,226 articles) and Scopus (4483 articles) resulted in a much smaller number of non‐English‐language articles (Figure 1). Searches on the 11 local search systems for 13 languages identified 42,331 articles, but this number is still much smaller than that based on Google Scholar. When comparing search results within each language, searches on Google Scholar identified many more articles than other search systems for most languages except Portuguese, Spanish, Japanese, and Russian, for which searches on the local search systems identified the largest number of articles (Figure 1).

**FIGURE 2:**
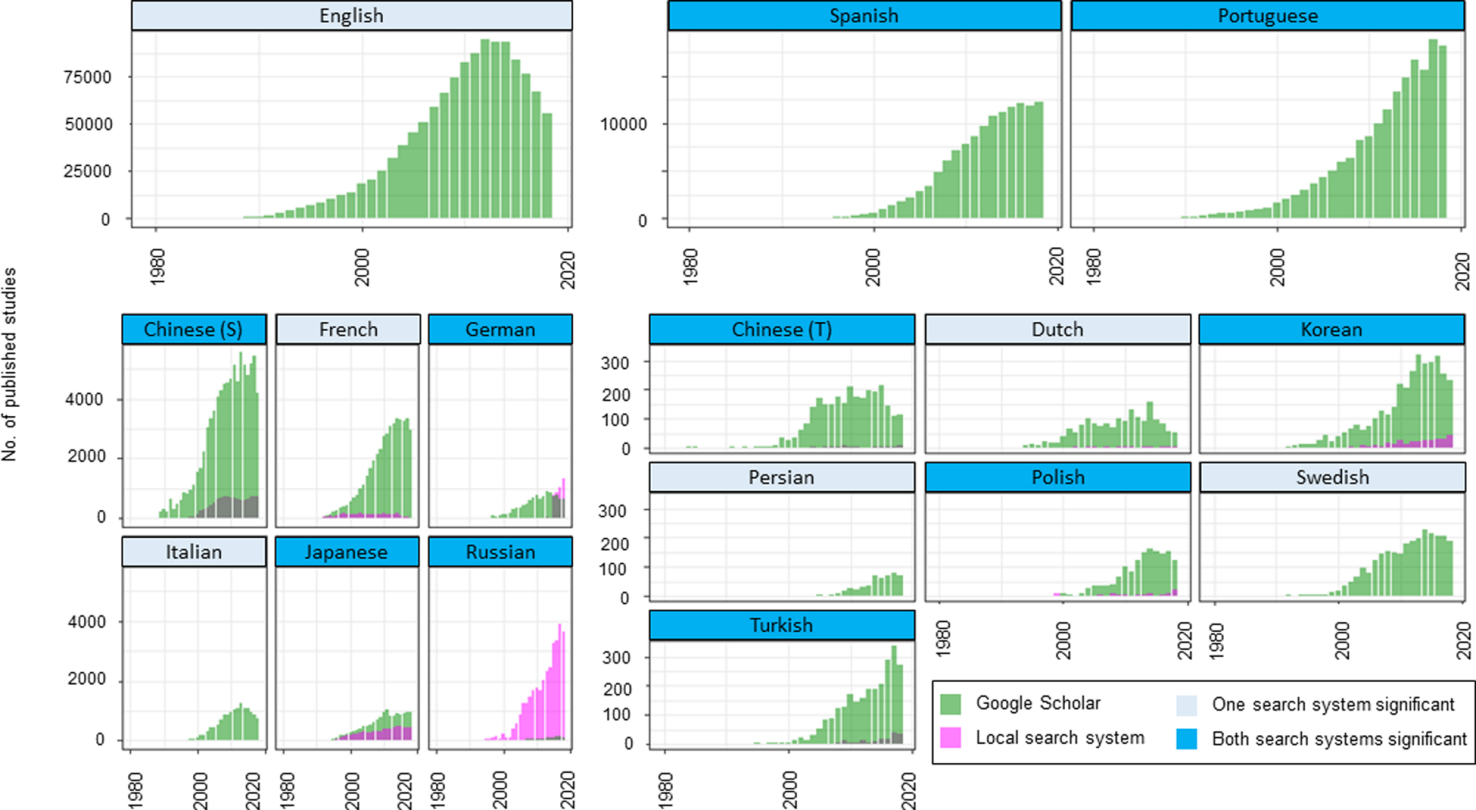
Changes in the number of biodiversity conservation articles published from 1980 to 2018 in 16 languages based on searches for the keywords *biodiversity* AND *conservation* (translated into each language) (translated keywords in Appendix S1) in Google Scholar and local literature search systems (S, simplified; T, traditional). See Appendix S2 for the same results based on Web of Science and Scopus

**FIGURE 3:**
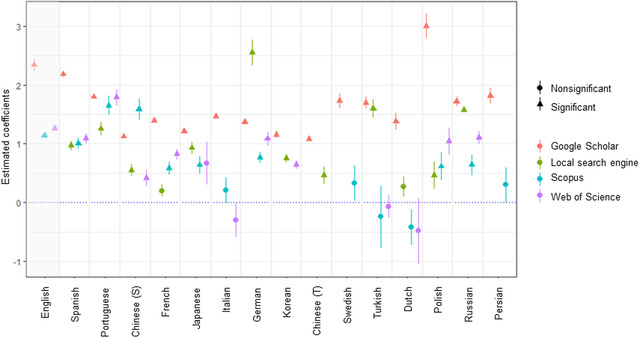
Estimated change in the number of biodiversity conservation articles published from 1980 to 2018 in 16 languages based on searches in different literature search systems (dots, estimated coefficient of the year term in the generalized linear model [response variable, number of articles published each year; explanatory variable, year]; bars, SE; S, simplified; T, traditional). Because neither Scopus nor Web of Science distinguish between simplified and traditional Chinese, search results for Chinese in general from the two search engines are shown under simplified Chinese. The order of languages on the x‐axis is based on total number of publications in 2018 identified using Google Scholar (English, most publications; Persian, fewest publications) (details in Figure 1)

In most languages and on most search systems, conservation articles increased exponentially in the late 1990s (Figure 2) (results from Web of Science and Scopus in Appendix S2). The number of conservation articles published each year has increased significantly over the past 39 years for most languages (Figure 3 & Appendix S3). For Spanish, Portuguese, German, Korean, and Russian, the number of conservation articles published each year increased at a rate similar to, or even faster than, English‐language articles (Figure 2). However, the number of published conservation articles identified through Google Scholar declined in the early to mid‐2010s in English and some non‐English languages (Figure 2). This result was corroborated by the better performance of the model with the quadratic year term (Appendix S2). Web of Science and Scopus tended to show a lower rate of increase than Google Scholar and local search systems, and they showed no growth for Italian, Swedish, Turkish, Dutch, and Persian (Figure 2 & Appendix S2). The number of articles published annually in Italian, Turkish, and Dutch showed a nonsignificant change based on Scopus and Web of Science, but showed a significant increase based on Google Scholar (and the local search system for Turkish; Appendix S3).

**FIGURE 4:**
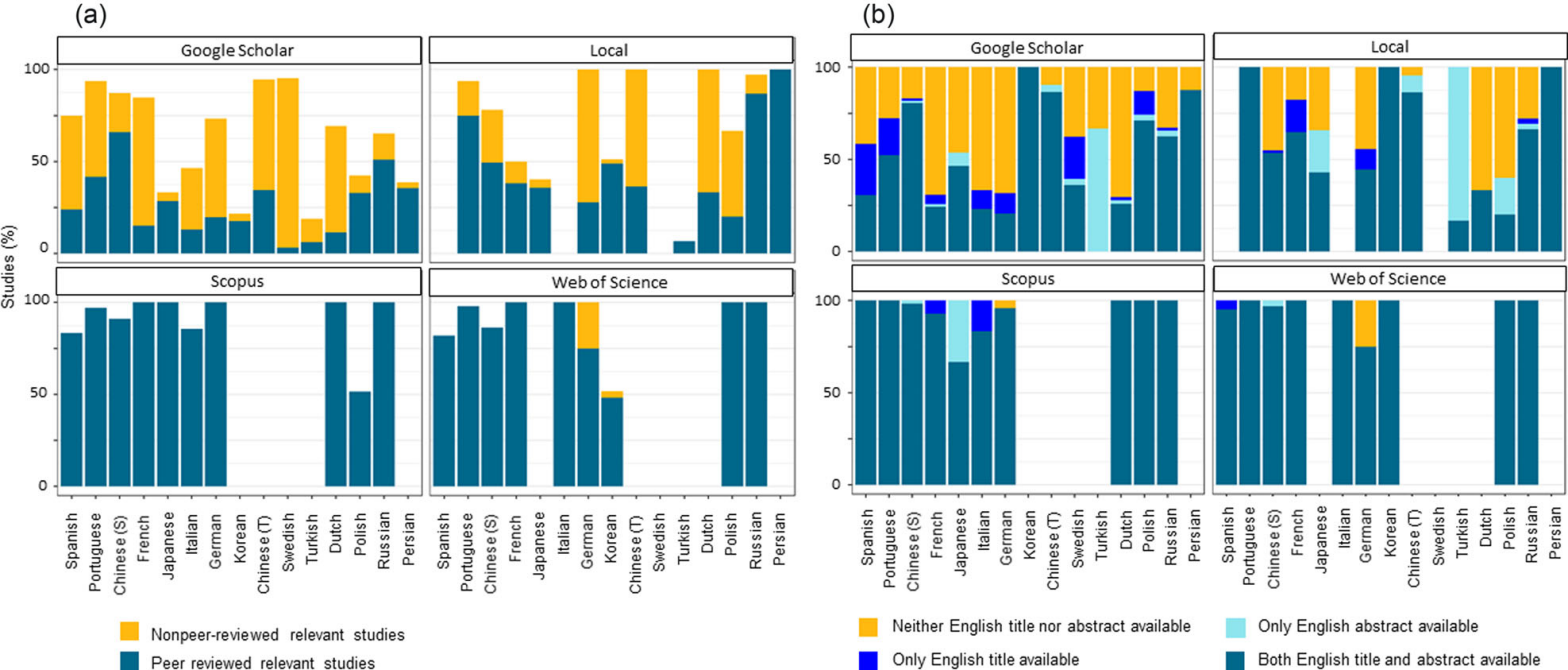
Percentage (a) of nonpeer‐reviewed and peer‐reviewed studies that mentioned biodiversity conservation (one study represents one published article) identified in four types of literature search systems for 15 non‐English languages in 2018 and (b) of peer‐reviewed, relevant, non‐English‐language studies with neither title nor abstract available in English, with only title available in English, with only abstract available in English, and with title and abstract available in English (S, simplified; T, traditional). Data are unavailable in some language‐search system combinations because local search engines for the language could not be identified or the search in the search system did not identify any article in the language. The order of languages on the x‐axis is based on the total number of publications in 2018 identified using Google Scholar

Although the percentage of relevant studies was generally high (> 75%) across search systems for most languages (e.g., over 86% for simplified Chinese), it varied substantially among search systems and languages. Scopus showed the highest mean percentage of relevant studies across languages (91%), followed by Web of Science (89%), local literature search systems (68%), and Google Scholar (66%) (Figure 4a). The most relevant articles identified on Scopus and Web of Science were peer‐reviewed studies (both 91% on average). In comparison, the proportion of peer‐reviewed relevant articles was considerably lower in both Google Scholar and local literature search systems (26% and 42%, respectively, on average) (Figure 4a).

Most peer‐reviewed relevant articles identified on Scopus and Web of Science provided the title and abstract in English. In contrast, a considerable proportion of the peer‐reviewed relevant articles identified on Google Scholar (39% on average) and local literature search systems (25%) provided neither the title nor the abstract in English, making them, in theory, undetectable when searching with English keywords (Figure 4b).

## DISCUSSION

The common assumption that the number of non‐English‐language publications is decreasing was not supported by our results. We found that the number of non‐English‐language articles on biodiversity conservation published each year actually been increased over the past 39 years in 12 of the 15 languages. For Dutch, Italian, and Turkish, there was a significant increase in the number of published articles based on Google Scholar or local search systems and a nonsignificant change based on Web of Science and Scopus. Given a sizable number of non‐English‐language journals and articles are indexed by neither Web of Science nor Scopus (Figure 1), this result indicates that the number of conservation articles in Dutch, Italian, and Turkish may have also increased, or at least remained constant, over the same period. The reason for the slower growth of articles in these three languages is unknown, but our results indicated that speakers of these three languages may especially prefer publishing their work in English, compared with speakers of the other 12 languages.

This result suggests that an increasing number of people, presumably those whose first language is not English, choose to publish their conservation work in English and a non‐English language. This choice may stem from one of the following reasons. First, language barriers, which cause difficulties in publishing in English, may force non‐native English speakers to publish their work in their first language. Science written by non‐native English speakers tends to be considered of low quality (Politzer‐Ahles et al., 2020) and is often rejected by English‐language journals (Ramírez‐Castañeda, 2020) simply because of the writing style. Non‐native English speakers, especially those in the early stages of their career and those with low socioeconomic status who are disproportionately affected by language barriers (due to high costs of learning English as a second language and using professional editing services [Ramírez‐Castañeda, 2020]), may consequently turn to journals published in their first language as more promising publication outlets. Second, non‐native English speakers may believe that the topic of their work is not of international importance (e.g., because it is about species and ecosystems that are specific to their country). Thus, they conclude that their results are not substantial enough to be published in international journals, leading to submission to and publication in local non‐English‐language journals. Finally, people may also deliberately choose to publish their work in a non‐English language for constructive reasons. For example, the publication of science only in English represents a barrier to the use of scientific knowledge by decision makers (Amano et al., 2016). Recognizing this language barrier, scientists may choose to publish their work in the language of the country, where they aim to disseminate their work to decision makers and the general public. Both types of language barriers (i.e., language barriers to publications and to the use of science in decision‐making) continue to be pervasive issues in countries where English is not widely spoken, which may explain the increasing trend of non‐English‐language articles on biodiversity conservation.

We observed a marked variation in the number of non‐English‐language articles identified among the different literature search systems. Overall, Google Scholar identified the largest number of non‐English‐language articles for most languages, followed by local search systems. In contrast, Scopus and Web of Science identified less than one‐twentieth and one‐ninth the number of non‐English‐language articles identified on Google Scholar, respectively. The percentages of peer‐reviewed relevant articles were much lower in Google Scholar than Scopus and Web of Science, which was also reported by Haddaway et al. (2015). However, even when focusing only on peer‐reviewed relevant articles, Google Scholar seems to identify many more non‐English‐language articles than Scopus and Web of Science (Appendix S4). Further, it is now increasingly recognized that gray literature, which is often not peer reviewed, also plays an important role in environmental evidence synthesis (Haddaway & Bayliss, 2015). Google Scholar allowed us to identify such potentially important gray literature, whereas Scopus and Web of Science do not normally index gray literature (blue bars in Figure 4a). Local search systems identified far more articles than Google Scholar for some languages, such as Portuguese, Spanish, Japanese, and Russian. This finding indicates that the most effective search system for identifying non‐English‐language literature varies among languages.

We also found that searching with non‐English‐language keywords on Google Scholar and local literature search systems identified articles with neither the title nor the abstract available in English (approximately 20% of all articles identified), many of which were not discoverable with English keywords on Scopus and Web of Science. Overall, Google Scholar seems to be the most effective system in many languages, but using multiple search systems, especially Google Scholar and local search systems, is important for identifying important non‐English‐language articles.

One potential limitation of our study is that we searched non‐English articles on a very broad topic (anything related to biodiversity and conservation) with a simple search string. The patterns we identified could be different for articles on more specific topics, although a recent study also showed that peer‐reviewed studies on the effectiveness of conservation actions are being published at an increasing rate in many languages (Amano et al., 2021). Future studies should reassess how the availability of non‐English‐language literature might vary among, for example, taxonomic groups (e.g., birds vs. insects) and research topics (e.g., studies on ecological threats, the state of biodiversity, and the effectiveness of conservation intervention) to better understand where non‐English‐language literature can fill knowledge gaps that are not covered by the English literature.

Second, we did not consider the potential duplication of articles among languages. Some authors whose first language is not English may publish the same work in both their first language (e.g., as a thesis) and English (e.g., as an academic journal article) or the same paper may be found with searches in multiple languages. Considering that Google Scholar also covers gray literature, the results based on Google Scholar searches could be especially vulnerable to the effect of such duplicated articles. However, this limitation is unlikely to undermine our conclusion that the number of non‐English‐language articles is increasing because the results were consistent across languages and search systems, including the Web of Science and Scopus (Figure 3 & Appendix S3), which is essentially restricted to academic journal papers.

Third, we assessed only 15 non‐English languages following Amano et al. (2016). We are aware that many languages were not covered in this study, although the languages assessed likely cover a large number of the languages spoken in the Global North and South. Finally, although we assessed the proportion of peer‐reviewed articles to measure the quality of studies published in each language, study quality depends on many other factors, such as study design and sample size (Christie et al., 2020). Thus, our results do not necessarily indicate that high‐quality research has been increasingly published in non‐English languages. A recent study showed that non‐English‐language studies adopt less robust study designs than English‐language studies in biodiversity conservation (Amano et al., 2021). In healthcare, previous studies comparing study quality between languages have reported mixed results, and it is thus recommended that “studies published in languages other than English [should not] be generally excluded [from evidence syntheses] for the reason of study quality” (Song et al., 2010). Testing differences in conservation and ecology study quality between languages is a priority for future studies.

We found that the number of non‐English‐language conservation articles increased in most languages. Recent studies have also shown between‐language bias in study characteristics and statistical results (Konno et al., 2020; Amano et al., 2021). Focusing on meta‐analysis in ecology and conservation, Konno et al. (2020) demonstrated that excluding non‐English‐language studies can cause substantial changes in overall mean effect sizes and even their direction (Konno et al., 2020). Amano et al. (2021) identified non‐English‐language studies providing evidence of the effectiveness of conservation actions, especially in areas and for species with little or even no English‐language evidence. Together, these findings indicate that the importance of non‐English‐language articles in ecological evidence syntheses is not diminishing. To derive robust conclusions in evidence syntheses and correctly inform conservation actions and policies, synthesizing the best available knowledge in an unbiased manner is essential. This seems possible only by incorporating scientific knowledge that has long been accumulating and continues to be produced in languages other than English.

Scientific literature written in non‐English languages has largely been ignored at the international level (Lynch et al., 2021), but our results indicate that conservation science, practices, and policies can greatly benefit from facilitating the better use of non‐English‐language literature. This could be achieved through, for example, involving native speakers of major non‐English languages in evidence syntheses to search and screen literature in multiple languages or by integrating machine translation into automated or semiautomated evidence synthesis tools. Well‐known international literature search systems, such as Web of Science and Scopus, and other search systems that fully include non‐English‐language literature should be used. Using Google Scholar, together with local, language‐specific search systems, such as those listed in Table 1, would be a good starting point for such multilanguage literature searches. Increasing the visibility of non‐English‐language literature would also be effective. When publishing in non‐English languages, authors should make sure to provide an English title and abstract or upload preprints on international repositories. Similarly, creating a list of non‐English‐language journals in the relevant discipline may also be useful (e.g., see a list of non‐English‐language journals in ecology and conservation https://translatesciences.com/resources/#non‐english‐journals).

Our results show that despite the increased use of English as a common language of science, language continues to be a factor in biodiversity conservation. Although overcoming the issue of language barriers is a challenging process, addressing this issue can boost understanding of life on Earth and improve decision‐making for conservation.

## Supporting information


**Table S1**. List of keywords that were used to search non‐English‐language literature.
**Figure S2**. The number of conservation articles published between 1980 and 2018 in 16 languages based on searches with Scopus and Web of Science. Both Scopus and Web of Science do not distinguish simplified and traditional Chinese, so search results for Chinese can include articles in both languages. Here, Chinese (S) and Chinese (T) represent simplified and traditional Chinese, respectively.
**Table S3**. Results of generalized linear models (response variable: the number of articles published each year, explanatory variable: year) showing the annual rate of changes in the number of conservation articles published each year in 16 languages based on searches with different search systems.
**Figure S4**. The estimated number of peer‐reviewed (red) and non‐peer‐reviewed (blue) relevant (i.e., those that mention biodiversity and conservation) articles published in 2018 for different language‐search systems combinations.
**Table S5**. Number of year‐wise published studies on biodiversity conservation in 16 languages using different search systems.

Supplementary material
