# Supplementary material for: Growth of non‐English‐language literature on biodiversity conservation
Source: Conserv Biol. 2022 Mar 24;36(4):e13883. doi: 10.1111/cobi.13883 (PMC9539909; doi:10.1111/cobi.13883)
Supplement: Supplementary file 1 — Table S1. List of keywords that were used to search non‐English‐language literature. Figure S2. The number of conservation articles published between 1980 and 2018 in 16 languages based on searches with Scopus and Web of Science. Both Scopus and Web of Science do not distinguish simplified and traditional Chinese, so search results for Chinese can include articles in both languages. Here, Chinese (S) and Chinese (T) represent simplified and traditional Chinese, respectively. Table S3. Results of generalized linear models (response variable: the number of articles published each year, explanatory variable: year) showing the annual rate of changes in the number of conservation articles published each year in 16 languages based on searches with different search systems. Figure S4. The estimated number of peer‐reviewed (red) and non‐peer‐reviewed (blue) relevant (i.e., those that mention biodiversity and conservation) articles published in 2018 for different language‐search systems combinations. Table S5. Number of year‐wise published studies on biodiversity conservation in 16 languages using different search systems. [file COBI-36-0-s001.docx]

**Appendix I**

**Table S1.** List of keywords that were used to search non-English-language literature.

| **Language** | **Translated keywords** |
| --- | --- |
| Spanish | “biodiversidad” “conservación” |
| Portuguese | "biodiversidade" "conservação" |
| Chinese (Simplified) | “生物多样性” and “保护” |
| Chinese (Traditional) | "生物多樣性" and "保育" |
| French | "biodiversité" |
| German | “Biodiversität” und “Naturschutz” |
| Japanese | “生物多様性” and “保全” |
| Korean | "biodiversity" and "conservation" |
| Polish | “Bioróżnorodność” |
| Turkish | "biodiversity" and "conservation" |
| Russian | “биоразнообразие” and “сохранение” |
| Persian | "تنوع زیستی” و “حفاظت" |
| Dutch | “biodiversiteit” and “bescherming” |
| Italian | Biodiversità and Conservazione |
| Swedish | "biologisk mångfald" and "bevarande" |

**Appendix II**


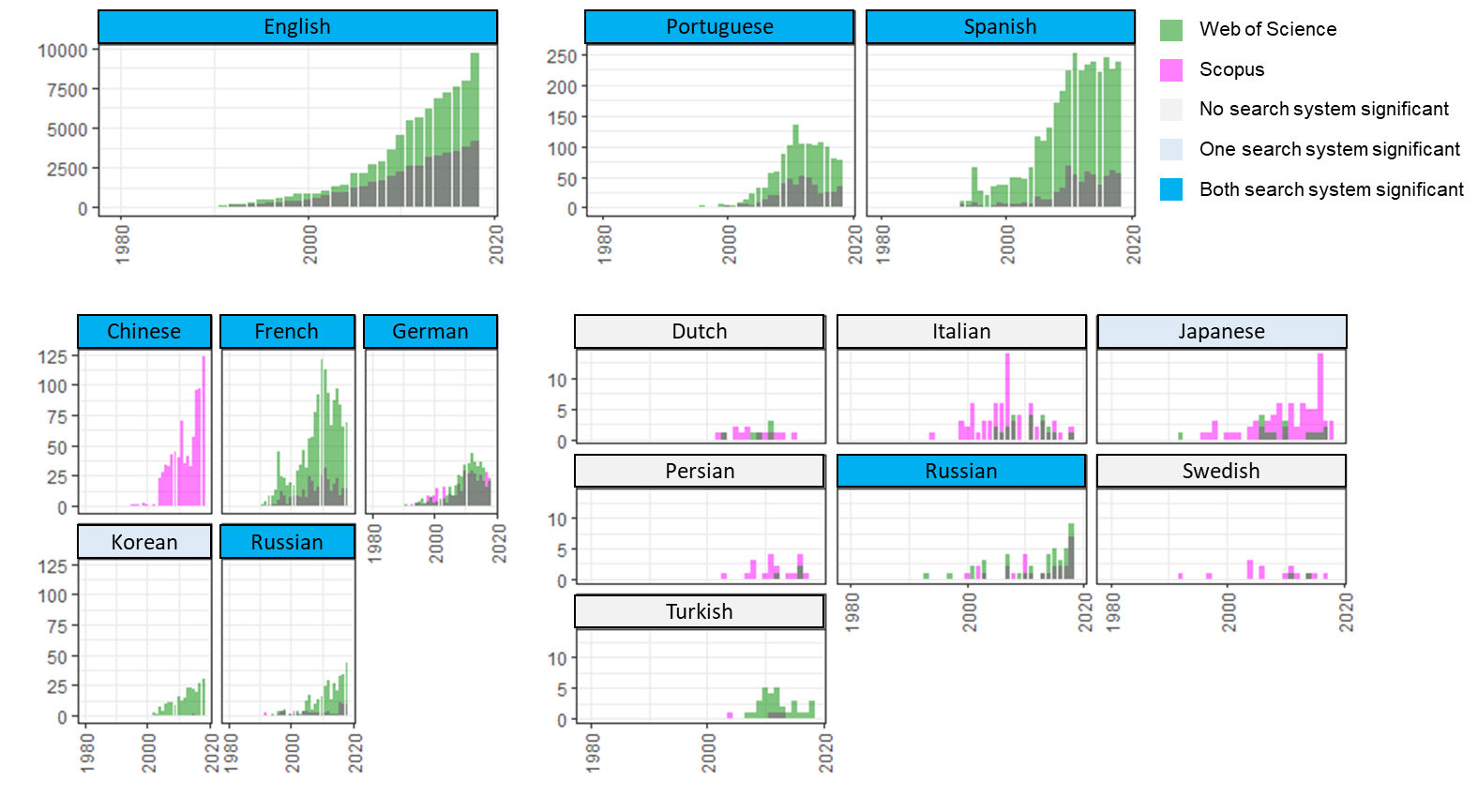
**Figure S2.** The number of conservation articles published between 1980 and 2018 in 16 languages based on searches with Scopus and Web of Science. Both Scopus and Web of Science do not distinguish simplified and traditional Chinese, so search results for Chinese can include articles in both languages. Here, Chinese (S) and Chinese (T) represent simplified and traditional Chinese, respectively.

**Appendix III**

**Table S3.** Results of generalized linear models (response variable: the number of articles published each year, explanatory variable: year) showing the annual rate of changes in the number of conservation articles published each year in 16 languages based on searches with different search systems. As some languages seemed to show a non-linear pattern in changes in the number of articles over years, we compared models with and without the quadratic term using AIC. Significant results and the model with lower AIC are shown in bold. The AIC is ‘NA’ in some language-search system combinations because of small sample size (i.e., only a small number of non-zero values were available). As both Scopus and Web of Science do not distinguish simplified and traditional Chinese, search results for Chinese in general are shown under simplified Chinese. Here, ‘L’ is linear model and ‘Q’ is quadratic model.

| **Language** | **Search system** | **Year** | | | | **Year^2^** | | | | **AIC** | **Model** |
| --- | --- | --- | --- | --- | --- | --- | --- | --- | --- | --- | --- |
|  |  | **Estimate** | **SE** | **z** | **p** | **Estimate** | **SE** | **z** | **p** |  |  |
| Simplified Chinese | Google Scholar | 0.19 | 0.02 | 9.05 | **< 0.0001** |  |  |  |  | 602.38 | L |
|  | Google Scholar | 0.21 | 0.01 | 24.87 | **< 0.0001** | -0.01 | 0.001 | -11.06 | **< 0.0001** | **548.09** | **Q** |
|  | Local | 0.09 | 0.02 | 4.41 | **< 0.0001** |  |  |  |  | 289.51 | L |
|  | Local | 0.10 | 0.01 | 11.79 | **< 0.0001** | -0.01 | 0.002 | -8.18 | **< 0.0001** | **262.12** | **Q** |
|  | Scopus | 0.22 | 0.03 | 6.96 | **< 0.0001** |  |  |  |  | 186.66 | L |
|  | Scopus | 0.25 | 0.03 | 8.88 | **< 0.0001** | -0.01 | 0.004 | -3.39 | **< 0.0001** | **179.31** | **Q** |
|  | Web of Science | 0.07 | 0.03 | 2.77 | 0.006 |  |  |  |  | 140.96 | L |
|  | Web of Science | 0.09 | 0.02 | 3.79 | **< 0.0001** | -0.01 | 0.005 | -2.58 | 0.01 | **136.91** | **Q** |
| Traditional Chinese | Google Scholar | 0.20 | 0.02 | 10.26 | **< 0.0001** |  |  |  |  | 308.43 | L |
|  | Google Scholar | 0.26 | 0.02 | 13.32 | **< 0.0001** | -0.01 | 0.002 | -7.39 | **< 0.0001** | **288.84** | **Q** |
|  | Local | 0.08 | 0.03 | 3.16 | 0.002 |  |  |  |  | **84.37** | **L** |
|  | Local | 0.09 | 0.03 | 3.07 | 0.002 | 0.00 | 0.01 | -0.84 | 0.4 | 85.63 | Q |
| Dutch | Google Scholar | 0.15 | 0.02 | 6.73 | **< 0.0001** |  |  |  |  | 285.52 | L |
|  | Google Scholar | 0.17 | 0.01 | 16.59 | **< 0.0001** | -0.01 | 0.001 | -11.25 | **< 0.0001** | **228.81** | **Q** |
|  | Local | 0.04 | 0.02 | 1.59 | 0.11 |  |  |  |  | **81.56** | **L** |
|  | Local | 0.05 | 0.03 | 1.70 | 0.09 | 0.00 | 0.004 | -1.04 | 0.3 | 82.45 | Q |
|  | Scopus | -0.08 | 0.06 | -1.39 | 0.16 |  |  |  |  | 39.71 | L |
|  | Scopus | Model convergence problem due to small sample size | | | | | | | |  | Q |
|  | Web of Science | -0.10 | 0.14 | -0.76 | 0.4 |  |  |  |  | 30.99 | L |
|  | Web of Science | -0.23 | 0.23 | -1.02 | 0.31 | -0.05 | 0.04 | -1.24 | 0.21 | **30.79** | **Q** |
| English | Google Scholar | 0.21 | 0.01 | 18.16 | **< 0.0001** |  |  |  |  | 775.94 | L |
|  | Google Scholar | 0.20 | 0.0004 | 44.59 | **< 0.0001** | -0.01 | 0.0004 | -12.00 | **< 0.0001** | **721.47** | **Q** |
|  | Scopus | 0.17 | 0.01 | 12.35 | **< 0.0001** |  |  |  |  | 454.15 | L |
|  | Scopus | 0.18 | 0.01 | 28.78 | **< 0.0001** | -0.01 | 0.0008 | -8.33 | **< 0.0001** | **420.90** | **Q** |
|  | Web of Science | 0.17 | 0.01 | 14.85 | **< 0.0001** |  |  |  |  | 485.58 | L |
|  | Web of Science | 0.18 | 0.01 | 25.34 | **< 0.0001** | -0.01 | 0.0010 | -5.71 | **< 0.0001** | **466.56** | **Q** |
| French | Google Scholar | 0.21 | 0.01 | 16.27 | **< 0.0001** |  |  |  |  | 511.98 | L |
|  | Google Scholar | 0.23 | 0.004 | 54.65 | **< 0.0001** | -0.01 | 0.0003 | -23.28 | **< 0.0001** | **480.00** | **Q** |
|  | Local | 0.02 | 0.02 | 1.53 | 0.12 |  |  |  |  | 324.69 | L |
|  | Local | 0.03 | 0.01 | 4.13 | **< 0.0001** | -0.01 | 0.0010 | -8.90 | **< 0.0001** | **289.19** | **Q** |
|  | Scopus | 0.08 | 0.02 | 4.55 | **< 0.0001** |  |  |  |  | 169.98 | L |
|  | Scopus | 0.08 | 0.01 | 6.32 | **< 0.0001** | -0.01 | 0.0019 | -4.11 | **< 0.0001** | **158.66** | **Q** |
|  | Web of Science | 0.10 | 0.01 | 8.26 | **< 0.0001** |  |  |  |  | 244.52 | L |
|  | Web of Science | 0.10 | 0.01 | 11.17 | **< 0.0001** | -0.01 | 0.0013 | -4.09 | **< 0.0001** | **233.31** | **Q** |
| German | Google Scholar | 0.24 | 0.02 | 13.32 | **< 0.0001** |  |  |  |  | 397.52 | L |
|  | Google Scholar | 0.29 | 0.01 | 35.67 | **< 0.0001** | -0.01 | 0.0005 | -21.49 | **< 0.0001** | **310.11** | **Q** |
|  | Local | 0.38 | 0.03 | 13.09 | **< 0.0001** |  |  |  |  | **187.72** | **L** |
|  | Local | 0.35 | 0.03 | 10.81 | **< 0.0001** | 0.01 | 0.01 | 1.38 | 0.17 | 188.05 | Q |
|  | Scopus | 0.10 | 0.01 | 7.38 | **< 0.0001** |  |  |  |  | 162.65 | L |
|  | Scopus | 0.11 | 0.01 | 8.41 | **< 0.0001** | 0.00 | 0.0018 | -2.79 | 0.005 | **157.44** | **Q** |
|  | Web of Science | 0.13 | 0.02 | 8.77 | **< 0.0001** |  |  |  |  | 174.07 | L |
|  | Web of Science | 0.15 | 0.02 | 9.85 | **< 0.0001** | -0.01 | 0.0018 | -4.06 | **< 0.0001** | **163.38** | **Q** |
| Italian | Google Scholar | 0.25 | 0.02 | 15.85 | **< 0.0001** |  |  |  |  | 396.51 | L |
|  | Google Scholar | 0.36 | 0.01 | 42.05 | **< 0.0001** | -0.01 | 0.0004 | -28.74 | **< 0.0001** | **331.52** | **Q** |
|  | Local | Couldn't find an appropriate local search system | | | | | | | | | |
|  | Scopus | 0.03 | 0.04 | 0.75 | 0.45 |  |  |  |  | 114.62 | L |
|  | Scopus | 0.04 | 0.03 | 1.41 | 0.16 | -0.02 | 0.005 | -3.96 | **< 0.0001** | **101.80** | **Q** |
|  | Web of Science | -0.07 | 0.07 | -1.00 | 0.31 |  |  |  |  | **51.53** | **L** |
|  | Web of Science | -0.09 | 0.08 | -1.15 | 0.25 | -0.02 | 0.02 | -0.93 | 0.35 | 52.65 | Q |
| Japanese | Google Scholar | 0.23 | 0.02 | 10.20 | **< 0.0001** |  |  |  |  | 435.47 | L |
|  | Google Scholar | 0.26 | 0.01 | 24.85 | **< 0.0001** | -0.01 | 0.001 | -13.37 | **< 0.0001** | **362.12** | **Q** |
|  | Local | 0.11 | 0.02 | 7.29 | **< 0.0001** |  |  |  |  | 342.67 | L |
|  | Local | 0.12 | 0.01 | 19.03 | **< 0.0001** | -0.01 | 0.001 | -9.18 | **< 0.0001** | **304.65** | **Q** |
|  | Scopus | 0.09 | 0.02 | 4.08 | **< 0.0001** |  |  |  |  | **93.74** | **L** |
|  | Scopus | 0.10 | 0.03 | 3.93 | **< 0.0001** | 0.00 | 0.004 | -0.71 | 0.47 | 95.24 | Q |
|  | Web of Science | 0.08 | 0.05 | 1.72 | 0.09 |  |  |  |  | **57.39** | **L** |
|  | Web of Science | 0.09 | 0.06 | 1.71 | 0.09 | 0.00 | 0.01 | -0.56 | 0.57 | 59.07 | Q |
| Korean | Google Scholar | 0.15 | 0.01 | 16.67 | **< 0.0001** |  |  |  |  | 252.49 | L |
|  | Google Scholar | 0.15 | 0.01 | 23.37 | **< 0.0001** | 0.00 | 0.001 | -5.49 | **< 0.0001** | **233.44** | **Q** |
|  | Local | 0.15 | 0.01 | 10.71 | **< 0.0001** |  |  |  |  | 104.32 | L |
|  | Local | 0.17 | 0.02 | 9.15 | **< 0.0001** | -0.01 | 0.003 | -2.12 | 0.03 | **101.60** | **Q** |
|  | Web of Science | Model convergence problem due to small sample size | | | | | | | | | L |
|  | Web of Science | 0.14 | 0.02 | 7.69 | **< 0.0001** | -0.01 | 0.004 | -1.92 | 0.05 | 91.68 | Q |
| Persian | Google Scholar | 0.28 | 0.02 | 11.57 | **< 0.0001** |  |  |  |  | 124.78 | L |
|  | Google Scholar | 0.38 | 0.04 | 10.26 | **< 0.0001** | -0.02 | 0.003 | -4.77 | **< 0.0001** | **110.27** | **Q** |
|  | Local | Not analyzed due to small sample size | | | | | | | | |  |
|  | Scopus | 0.06 | 0.07 | 0.96 | 0.34 |  |  |  |  | **52.69** | **L** |
|  | Scopus | 0.08 | 0.07 | 1.16 | 0.25 | -0.02 | 0.02 | -1.37 | 0.17 | 52.75 | Q |
|  | Web of Science | Not analyzed due to small sample size | | | | | | | | |  |
| Polish | Google Scholar | 0.26 | 0.02 | 12.07 | **< 0.0001** |  |  |  |  | 217.93 | L |
|  | Google Scholar | 0.44 | 0.04 | 11.24 | **< 0.0001** | -0.01 | 0.002 | -6.73 | **< 0.0001** | **201.34** | **Q** |
|  | Local | 0.08 | 0.03 | 2.29 | 0.02 |  |  |  |  | 106.16 | L |
|  | Local | 0.08 | 0.03 | 2.34 | 0.02 | 0.01 | 0.01 | 2.14 | 0.03 | **104.03** | **Q** |
|  | Scopus | 0.11 | 0.04 | 2.57 | 0.01 |  |  |  |  | 61.52 | L |
|  | Scopus | 0.09 | 0.04 | 2.37 | 0.02 | 0.01 | 0.01 | 1.68 | 0.09 | **61.20** | **Q** |
|  | Web of Science | 0.14 | 0.03 | 4.36 | **< 0.0001** |  |  |  |  | 81.09 | L |
|  | Web of Science | Model convergence problem due to small sample size | | | | | | | | | Q |
| Portuguese | Google Scholar | 0.28 | 0.01 | 25.60 | **< 0.0001** |  |  |  |  | 518.63 | L |
|  | Google Scholar | 0.29 | 0.01 | 34.68 | **< 0.0001** | -0.01 | 0.001 | -7.32 | **< 0.0001** | **492.92** | **Q** |
|  | Local | 0.18 | 0.02 | 8.57 | **< 0.0001** |  |  |  |  | 150.65 | L |
|  | Local | Model convergence problem due to small sample size | | | | | | | | | Q |
|  | Scopus | 0.2005 | 0.0244 | 8.2233 | **< 0.0001** |  |  |  |  | 171.23 | L |
|  | Scopus | 0.28 | 0.03 | 8.80 | **< 0.0001** | -0.02 | 0.003 | -5.33 | **< 0.0001** | **154.45** | **Q** |
|  | Web of Science | 0.22 | 0.02 | 10.78 | **< 0.0001** |  |  |  |  | 213.97 | L |
|  | Web of Science | 0.30 | 0.02 | 15.04 | **< 0.0001** | -0.02 | 0.002 | -9.72 | **< 0.0001** | **180.74** | **Q** |
| Russian | Google Scholar | 0.19 | 0.01 | 17.25 | **< 0.0001** |  |  |  |  | 194.41 | L |
|  | Google Scholar | 0.23 | 0.01 | 15.81 | **< 0.0001** | -0.01 | 0.001 | -5.48 | **< 0.0001** | **171.70** | **Q** |
|  | Local | 0.28 | 0.02 | 13.10 | **< 0.0001** |  |  |  |  | 414.81 | L |
|  | Local | 0.30 | 0.01 | 23.99 | **< 0.0001** | -0.01 | 0.001 | -7.86 | **< 0.0001** | **376.90** | **Q** |
|  | Scopus | 0.08 | 0.02 | 3.77 | **< 0.0001** |  |  |  |  | **108.16** | **L** |
|  | Scopus | 0.07 | 0.02 | 3.38 | **< 0.0001** | 0.00 | 0.003 | 1.25 | 0.21 | 108.76 | Q |
|  | Web of Science | 0.15 | 0.02 | 9.98 | **< 0.0001** |  |  |  |  | **144.41** | **L** |
|  | Web of Science | 0.16 | 0.02 | 9.07 | **< 0.0001** | 0.00 | 0.002 | -1.07 | 0.29 | 145.32 | Q |
| Spanish | Google Scholar | 0.19 | 0.01 | 34.87 | **< 0.0001** |  |  |  |  | 572.42 | L |
|  | Google Scholar | 0.19 | 0.005 | 41.95 | **< 0.0001** | 0.00 | 0.0004 | -3.28 | 0.001 | **565.72** | **Q** |
|  | Local | 0.17 | 0.02 | 11.35 | **< 0.0001** |  |  |  |  | 132.35 | L |
|  | Local | 0.19 | 0.02 | 12.83 | **< 0.0001** | -0.01 | 0.002 | -3.62 | **< 0.0001** | **122.87** | **Q** |
|  | Scopus | 0.13 | 0.01 | 10.26 | **< 0.0001** |  |  |  |  | 180.71 | L |
|  | Scopus | 0.13 | 0.01 | 10.20 | **< 0.0001** | 0.00 | 0.002 | -0.23 | 0.82 | 182.66 | Q |
|  | Web of Science | 0.13 | 0.01 | 10.98 | **< 0.0001** |  |  |  |  | 283.80 | L |
|  | Web of Science | 0.13 | 0.01 | 14.82 | **< 0.0001** | 0.00 | 0.001 | -3.69 | **< 0.0001** | **274.93** | **Q** |
| Swedish | Google Scholar | 0.20 | 0.02 | 11.01 | **< 0.0001** |  |  |  |  | 268.68 | L |
|  | Google Scholar | 0.22 | 0.01 | 24.58 | **< 0.0001** | -0.01 | 0.001 | -14.18 | **< 0.0001** | **205.56** | **Q** |
|  | Local | Couldn't find an appropriate local search system | | | | | | | | |  |
|  | Scopus | 0.04 | 0.04 | 1.05 | 0.29 |  |  |  |  | **57.15** | **L** |
|  | Scopus | 0.05 | 0.04 | 1.13 | 0.26 | 0.00 | 0.01 | -0.79 | 0.40 | 58.51 | Q |
|  | Web of Science | Not analyzed due to small sample size | | | | | | | | |  |
| Turkish | Google Scholar | 0.22 | 0.02 | 14.44 | **< 0.0001** |  |  |  |  | 225.66 | L |
|  | Google Scholar | 0.25 | 0.01 | 20.75 | **< 0.0001** | -0.01 | 0.001 | -7.23 | **< 0.0001** | **199.59** | **Q** |
|  | Local | 0.26 | 0.03 | 10.00 | **< 0.0001** |  |  |  |  | **91.19** | **L** |
|  | Local | 0.29 | 0.05 | 6.21 | **< 0.0001** | 0.00 | 0.01 | -0.85 | 0.4 | 92.39 | Q |
|  | Scopus | Not analyzed due to small sample size | | | | | | | | | L |
|  | Scopus |  |  |  |  |  |  |  |  |  | Q |
|  | Web of Science | Not analyzed due to small sample size | | | | | | | | | L |
|  | Web of Science |  |  |  |  |  |  |  |  |  | Q |

**Appendix IV**

**
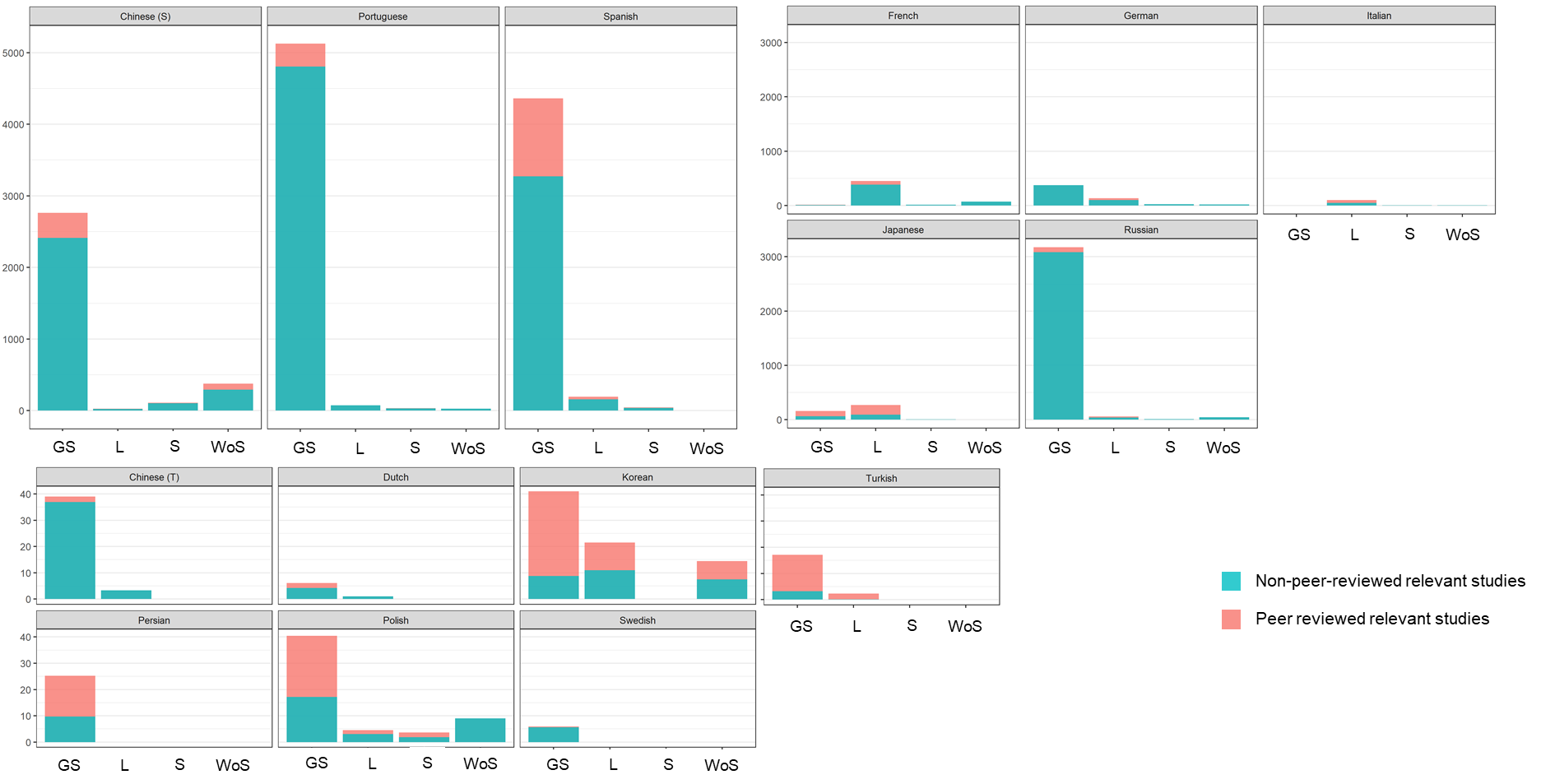
**

**Figure S4.** The estimated number of peer-reviewed (red) and non-peer-reviewed (blue) relevant (i.e., those that mention biodiversity and conservation) articles published in 2018 for different language-search systems combinations. Here, GS is for Google Scholar, L is Local search systems, S is Scopus, WoS is Web of Science. We estimated the number by multiplying the total number of articles identified in 2018 on each language-system combination (shown in Figure 1) by the proportion of peer-reviewed and non-peer-reviewed relevant articles (shown in Figure 4).

**Appendix V**

**Table S5.** Number of year-wise published studies on biodiversity conservation in 16 languages using different search systems.

| **Language** | **Year** | **Search engine** | **Search engine [group]** |
| --- | --- | --- | --- |
| Chinese (S) | 2018 | Airiti Library | Local |
| Chinese (S) | 2017 | Airiti Library | Local |
| Chinese (S) | 2016 | Airiti Library | Local |
| Chinese (S) | 2015 | Airiti Library | Local |
| Chinese (S) | 2014 | Airiti Library | Local |
| Chinese (S) | 2013 | Airiti Library | Local |
| Chinese (S) | 2012 | Airiti Library | Local |
| Chinese (S) | 2011 | Airiti Library | Local |
| Chinese (S) | 2010 | Airiti Library | Local |
| Chinese (S) | 2009 | Airiti Library | Local |
| Chinese (S) | 2008 | Airiti Library | Local |
| Chinese (S) | 2007 | Airiti Library | Local |
| Chinese (S) | 2006 | Airiti Library | Local |
| Chinese (S) | 2005 | Airiti Library | Local |
| Chinese (S) | 2004 | Airiti Library | Local |
| Chinese (S) | 2003 | Airiti Library | Local |
| Chinese (S) | 2002 | Airiti Library | Local |
| Chinese (S) | 2001 | Airiti Library | Local |
| Chinese (S) | 2000 | Airiti Library | Local |
| Chinese (S) | 1999 | Airiti Library | Local |
| Chinese (S) | 1998 | Airiti Library | Local |
| Chinese (S) | 1997 | Airiti Library | Local |
| Chinese (S) | 1996 | Airiti Library | Local |
| Chinese (S) | 1995 | Airiti Library | Local |
| Chinese (S) | 1994 | Airiti Library | Local |
| Chinese (S) | 1993 | Airiti Library | Local |
| Chinese (S) | 1992 | Airiti Library | Local |
| Chinese (S) | 1991 | Airiti Library | Local |
| Chinese (S) | 1990 | Airiti Library | Local |
| Chinese (S) | 1989 | Airiti Library | Local |
| Chinese (S) | 1988 | Airiti Library | Local |
| Chinese (S) | 1987 | Airiti Library | Local |
| Chinese (S) | 1986 | Airiti Library | Local |
| Chinese (S) | 1985 | Airiti Library | Local |
| Chinese (S) | 1984 | Airiti Library | Local |
| Chinese (S) | 1983 | Airiti Library | Local |
| Chinese (S) | 1982 | Airiti Library | Local |
| Chinese (S) | 1981 | Airiti Library | Local |
| Chinese (S) | 1980 | Airiti Library | Local |
| Chinese (S) | 2018 | Google.Scholar | Google Scholar |
| Chinese (S) | 2017 | Google.Scholar | Google Scholar |
| Chinese (S) | 2016 | Google.Scholar | Google Scholar |
| Chinese (S) | 2015 | Google.Scholar | Google Scholar |
| Chinese (S) | 2014 | Google.Scholar | Google Scholar |
| Chinese (S) | 2013 | Google.Scholar | Google Scholar |
| Chinese (S) | 2012 | Google.Scholar | Google Scholar |
| Chinese (S) | 2011 | Google.Scholar | Google Scholar |
| Chinese (S) | 2010 | Google.Scholar | Google Scholar |
| Chinese (S) | 2009 | Google.Scholar | Google Scholar |
| Chinese (S) | 2008 | Google.Scholar | Google Scholar |
| Chinese (S) | 2007 | Google.Scholar | Google Scholar |
| Chinese (S) | 2006 | Google.Scholar | Google Scholar |
| Chinese (S) | 2005 | Google.Scholar | Google Scholar |
| Chinese (S) | 2004 | Google.Scholar | Google Scholar |
| Chinese (S) | 2003 | Google.Scholar | Google Scholar |
| Chinese (S) | 2002 | Google.Scholar | Google Scholar |
| Chinese (S) | 2001 | Google.Scholar | Google Scholar |
| Chinese (S) | 2000 | Google.Scholar | Google Scholar |
| Chinese (S) | 1999 | Google.Scholar | Google Scholar |
| Chinese (S) | 1998 | Google.Scholar | Google Scholar |
| Chinese (S) | 1997 | Google.Scholar | Google Scholar |
| Chinese (S) | 1996 | Google.Scholar | Google Scholar |
| Chinese (S) | 1995 | Google.Scholar | Google Scholar |
| Chinese (S) | 1994 | Google.Scholar | Google Scholar |
| Chinese (S) | 1993 | Google.Scholar | Google Scholar |
| Chinese (S) | 1992 | Google.Scholar | Google Scholar |
| Chinese (S) | 1991 | Google.Scholar | Google Scholar |
| Chinese (S) | 1990 | Google.Scholar | Google Scholar |
| Chinese (S) | 1989 | Google.Scholar | Google Scholar |
| Chinese (S) | 1988 | Google.Scholar | Google Scholar |
| Chinese (S) | 1987 | Google.Scholar | Google Scholar |
| Chinese (S) | 1986 | Google.Scholar | Google Scholar |
| Chinese (S) | 1985 | Google.Scholar | Google Scholar |
| Chinese (S) | 1984 | Google.Scholar | Google Scholar |
| Chinese (S) | 1983 | Google.Scholar | Google Scholar |
| Chinese (S) | 1982 | Google.Scholar | Google Scholar |
| Chinese (S) | 1981 | Google.Scholar | Google Scholar |
| Chinese (S) | 1980 | Google.Scholar | Google Scholar |
| Chinese (S) | 2018 | Scopus | Scopus |
| Chinese (S) | 2017 | Scopus | Scopus |
| Chinese (S) | 2016 | Scopus | Scopus |
| Chinese (S) | 2015 | Scopus | Scopus |
| Chinese (S) | 2014 | Scopus | Scopus |
| Chinese (S) | 2013 | Scopus | Scopus |
| Chinese (S) | 2012 | Scopus | Scopus |
| Chinese (S) | 2011 | Scopus | Scopus |
| Chinese (S) | 2010 | Scopus | Scopus |
| Chinese (S) | 2009 | Scopus | Scopus |
| Chinese (S) | 2008 | Scopus | Scopus |
| Chinese (S) | 2007 | Scopus | Scopus |
| Chinese (S) | 2006 | Scopus | Scopus |
| Chinese (S) | 2005 | Scopus | Scopus |
| Chinese (S) | 2004 | Scopus | Scopus |
| Chinese (S) | 2003 | Scopus | Scopus |
| Chinese (S) | 2002 | Scopus | Scopus |
| Chinese (S) | 2001 | Scopus | Scopus |
| Chinese (S) | 2000 | Scopus | Scopus |
| Chinese (S) | 1999 | Scopus | Scopus |
| Chinese (S) | 1998 | Scopus | Scopus |
| Chinese (S) | 1997 | Scopus | Scopus |
| Chinese (S) | 1996 | Scopus | Scopus |
| Chinese (S) | 1995 | Scopus | Scopus |
| Chinese (S) | 1994 | Scopus | Scopus |
| Chinese (S) | 1993 | Scopus | Scopus |
| Chinese (S) | 1992 | Scopus | Scopus |
| Chinese (S) | 1991 | Scopus | Scopus |
| Chinese (S) | 1990 | Scopus | Scopus |
| Chinese (S) | 1989 | Scopus | Scopus |
| Chinese (S) | 1988 | Scopus | Scopus |
| Chinese (S) | 1987 | Scopus | Scopus |
| Chinese (S) | 1986 | Scopus | Scopus |
| Chinese (S) | 1985 | Scopus | Scopus |
| Chinese (S) | 1984 | Scopus | Scopus |
| Chinese (S) | 1983 | Scopus | Scopus |
| Chinese (S) | 1982 | Scopus | Scopus |
| Chinese (S) | 1981 | Scopus | Scopus |
| Chinese (S) | 1980 | Scopus | Scopus |
| Chinese (S) | 2018 | Web.of.Science | Web of Science |
| Chinese (S) | 2017 | Web.of.Science | Web of Science |
| Chinese (S) | 2016 | Web.of.Science | Web of Science |
| Chinese (S) | 2015 | Web.of.Science | Web of Science |
| Chinese (S) | 2014 | Web.of.Science | Web of Science |
| Chinese (S) | 2013 | Web.of.Science | Web of Science |
| Chinese (S) | 2012 | Web.of.Science | Web of Science |
| Chinese (S) | 2011 | Web.of.Science | Web of Science |
| Chinese (S) | 2010 | Web.of.Science | Web of Science |
| Chinese (S) | 2009 | Web.of.Science | Web of Science |
| Chinese (S) | 2008 | Web.of.Science | Web of Science |
| Chinese (S) | 2007 | Web.of.Science | Web of Science |
| Chinese (S) | 2006 | Web.of.Science | Web of Science |
| Chinese (S) | 2005 | Web.of.Science | Web of Science |
| Chinese (S) | 2004 | Web.of.Science | Web of Science |
| Chinese (S) | 2003 | Web.of.Science | Web of Science |
| Chinese (S) | 2002 | Web.of.Science | Web of Science |
| Chinese (S) | 2001 | Web.of.Science | Web of Science |
| Chinese (S) | 2000 | Web.of.Science | Web of Science |
| Chinese (S) | 1999 | Web.of.Science | Web of Science |
| Chinese (S) | 1998 | Web.of.Science | Web of Science |
| Chinese (S) | 1997 | Web.of.Science | Web of Science |
| Chinese (S) | 1996 | Web.of.Science | Web of Science |
| Chinese (S) | 1995 | Web.of.Science | Web of Science |
| Chinese (S) | 1994 | Web.of.Science | Web of Science |
| Chinese (S) | 1993 | Web.of.Science | Web of Science |
| Chinese (S) | 1992 | Web.of.Science | Web of Science |
| Chinese (S) | 1991 | Web.of.Science | Web of Science |
| Chinese (S) | 1990 | Web.of.Science | Web of Science |
| Chinese (S) | 1989 | Web.of.Science | Web of Science |
| Chinese (S) | 1988 | Web.of.Science | Web of Science |
| Chinese (S) | 1987 | Web.of.Science | Web of Science |
| Chinese (S) | 1986 | Web.of.Science | Web of Science |
| Chinese (S) | 1985 | Web.of.Science | Web of Science |
| Chinese (S) | 1984 | Web.of.Science | Web of Science |
| Chinese (S) | 1983 | Web.of.Science | Web of Science |
| Chinese (S) | 1982 | Web.of.Science | Web of Science |
| Chinese (S) | 1981 | Web.of.Science | Web of Science |
| Chinese (S) | 1980 | Web.of.Science | Web of Science |
| Chinese (T) | 2018 | Airiti Library | Local |
| Chinese (T) | 2017 | Airiti Library | Local |
| Chinese (T) | 2016 | Airiti Library | Local |
| Chinese (T) | 2015 | Airiti Library | Local |
| Chinese (T) | 2014 | Airiti Library | Local |
| Chinese (T) | 2013 | Airiti Library | Local |
| Chinese (T) | 2012 | Airiti Library | Local |
| Chinese (T) | 2011 | Airiti Library | Local |
| Chinese (T) | 2010 | Airiti Library | Local |
| Chinese (T) | 2009 | Airiti Library | Local |
| Chinese (T) | 2008 | Airiti Library | Local |
| Chinese (T) | 2007 | Airiti Library | Local |
| Chinese (T) | 2006 | Airiti Library | Local |
| Chinese (T) | 2005 | Airiti Library | Local |
| Chinese (T) | 2004 | Airiti Library | Local |
| Chinese (T) | 2003 | Airiti Library | Local |
| Chinese (T) | 2002 | Airiti Library | Local |
| Chinese (T) | 2001 | Airiti Library | Local |
| Chinese (T) | 2000 | Airiti Library | Local |
| Chinese (T) | 1999 | Airiti Library | Local |
| Chinese (T) | 1998 | Airiti Library | Local |
| Chinese (T) | 1997 | Airiti Library | Local |
| Chinese (T) | 1996 | Airiti Library | Local |
| Chinese (T) | 1995 | Airiti Library | Local |
| Chinese (T) | 1994 | Airiti Library | Local |
| Chinese (T) | 1993 | Airiti Library | Local |
| Chinese (T) | 1992 | Airiti Library | Local |
| Chinese (T) | 1991 | Airiti Library | Local |
| Chinese (T) | 1990 | Airiti Library | Local |
| Chinese (T) | 1989 | Airiti Library | Local |
| Chinese (T) | 1988 | Airiti Library | Local |
| Chinese (T) | 1987 | Airiti Library | Local |
| Chinese (T) | 1986 | Airiti Library | Local |
| Chinese (T) | 1985 | Airiti Library | Local |
| Chinese (T) | 1984 | Airiti Library | Local |
| Chinese (T) | 1983 | Airiti Library | Local |
| Chinese (T) | 1982 | Airiti Library | Local |
| Chinese (T) | 1981 | Airiti Library | Local |
| Chinese (T) | 1980 | Airiti Library | Local |
| Chinese (T) | 2018 | Google.Scholar | Google Scholar |
| Chinese (T) | 2017 | Google.Scholar | Google Scholar |
| Chinese (T) | 2016 | Google.Scholar | Google Scholar |
| Chinese (T) | 2015 | Google.Scholar | Google Scholar |
| Chinese (T) | 2014 | Google.Scholar | Google Scholar |
| Chinese (T) | 2013 | Google.Scholar | Google Scholar |
| Chinese (T) | 2012 | Google.Scholar | Google Scholar |
| Chinese (T) | 2011 | Google.Scholar | Google Scholar |
| Chinese (T) | 2010 | Google.Scholar | Google Scholar |
| Chinese (T) | 2009 | Google.Scholar | Google Scholar |
| Chinese (T) | 2008 | Google.Scholar | Google Scholar |
| Chinese (T) | 2007 | Google.Scholar | Google Scholar |
| Chinese (T) | 2006 | Google.Scholar | Google Scholar |
| Chinese (T) | 2005 | Google.Scholar | Google Scholar |
| Chinese (T) | 2004 | Google.Scholar | Google Scholar |
| Chinese (T) | 2003 | Google.Scholar | Google Scholar |
| Chinese (T) | 2002 | Google.Scholar | Google Scholar |
| Chinese (T) | 2001 | Google.Scholar | Google Scholar |
| Chinese (T) | 2000 | Google.Scholar | Google Scholar |
| Chinese (T) | 1999 | Google.Scholar | Google Scholar |
| Chinese (T) | 1998 | Google.Scholar | Google Scholar |
| Chinese (T) | 1997 | Google.Scholar | Google Scholar |
| Chinese (T) | 1996 | Google.Scholar | Google Scholar |
| Chinese (T) | 1995 | Google.Scholar | Google Scholar |
| Chinese (T) | 1994 | Google.Scholar | Google Scholar |
| Chinese (T) | 1993 | Google.Scholar | Google Scholar |
| Chinese (T) | 1992 | Google.Scholar | Google Scholar |
| Chinese (T) | 1991 | Google.Scholar | Google Scholar |
| Chinese (T) | 1990 | Google.Scholar | Google Scholar |
| Chinese (T) | 1989 | Google.Scholar | Google Scholar |
| Chinese (T) | 1988 | Google.Scholar | Google Scholar |
| Chinese (T) | 1987 | Google.Scholar | Google Scholar |
| Chinese (T) | 1986 | Google.Scholar | Google Scholar |
| Chinese (T) | 1985 | Google.Scholar | Google Scholar |
| Chinese (T) | 1984 | Google.Scholar | Google Scholar |
| Chinese (T) | 1983 | Google.Scholar | Google Scholar |
| Chinese (T) | 1982 | Google.Scholar | Google Scholar |
| Chinese (T) | 1981 | Google.Scholar | Google Scholar |
| Chinese (T) | 1980 | Google.Scholar | Google Scholar |
| Chinese (T) | 2018 | Scopus | Scopus |
| Chinese (T) | 2017 | Scopus | Scopus |
| Chinese (T) | 2016 | Scopus | Scopus |
| Chinese (T) | 2015 | Scopus | Scopus |
| Chinese (T) | 2014 | Scopus | Scopus |
| Chinese (T) | 2013 | Scopus | Scopus |
| Chinese (T) | 2012 | Scopus | Scopus |
| Chinese (T) | 2011 | Scopus | Scopus |
| Chinese (T) | 2010 | Scopus | Scopus |
| Chinese (T) | 2009 | Scopus | Scopus |
| Chinese (T) | 2008 | Scopus | Scopus |
| Chinese (T) | 2007 | Scopus | Scopus |
| Chinese (T) | 2006 | Scopus | Scopus |
| Chinese (T) | 2005 | Scopus | Scopus |
| Chinese (T) | 2004 | Scopus | Scopus |
| Chinese (T) | 2003 | Scopus | Scopus |
| Chinese (T) | 2002 | Scopus | Scopus |
| Chinese (T) | 2001 | Scopus | Scopus |
| Chinese (T) | 2000 | Scopus | Scopus |
| Chinese (T) | 1999 | Scopus | Scopus |
| Chinese (T) | 1998 | Scopus | Scopus |
| Chinese (T) | 1997 | Scopus | Scopus |
| Chinese (T) | 1996 | Scopus | Scopus |
| Chinese (T) | 1995 | Scopus | Scopus |
| Chinese (T) | 1994 | Scopus | Scopus |
| Chinese (T) | 1993 | Scopus | Scopus |
| Chinese (T) | 1992 | Scopus | Scopus |
| Chinese (T) | 1991 | Scopus | Scopus |
| Chinese (T) | 1990 | Scopus | Scopus |
| Chinese (T) | 1989 | Scopus | Scopus |
| Chinese (T) | 1988 | Scopus | Scopus |
| Chinese (T) | 1987 | Scopus | Scopus |
| Chinese (T) | 1986 | Scopus | Scopus |
| Chinese (T) | 1985 | Scopus | Scopus |
| Chinese (T) | 1984 | Scopus | Scopus |
| Chinese (T) | 1983 | Scopus | Scopus |
| Chinese (T) | 1982 | Scopus | Scopus |
| Chinese (T) | 1981 | Scopus | Scopus |
| Chinese (T) | 1980 | Scopus | Scopus |
| Chinese (T) | 2018 | Web.of.Science | Web of Science |
| Chinese (T) | 2017 | Web.of.Science | Web of Science |
| Chinese (T) | 2016 | Web.of.Science | Web of Science |
| Chinese (T) | 2015 | Web.of.Science | Web of Science |
| Chinese (T) | 2014 | Web.of.Science | Web of Science |
| Chinese (T) | 2013 | Web.of.Science | Web of Science |
| Chinese (T) | 2012 | Web.of.Science | Web of Science |
| Chinese (T) | 2011 | Web.of.Science | Web of Science |
| Chinese (T) | 2010 | Web.of.Science | Web of Science |
| Chinese (T) | 2009 | Web.of.Science | Web of Science |
| Chinese (T) | 2008 | Web.of.Science | Web of Science |
| Chinese (T) | 2007 | Web.of.Science | Web of Science |
| Chinese (T) | 2006 | Web.of.Science | Web of Science |
| Chinese (T) | 2005 | Web.of.Science | Web of Science |
| Chinese (T) | 2004 | Web.of.Science | Web of Science |
| Chinese (T) | 2003 | Web.of.Science | Web of Science |
| Chinese (T) | 2002 | Web.of.Science | Web of Science |
| Chinese (T) | 2001 | Web.of.Science | Web of Science |
| Chinese (T) | 2000 | Web.of.Science | Web of Science |
| Chinese (T) | 1999 | Web.of.Science | Web of Science |
| Chinese (T) | 1998 | Web.of.Science | Web of Science |
| Chinese (T) | 1997 | Web.of.Science | Web of Science |
| Chinese (T) | 1996 | Web.of.Science | Web of Science |
| Chinese (T) | 1995 | Web.of.Science | Web of Science |
| Chinese (T) | 1994 | Web.of.Science | Web of Science |
| Chinese (T) | 1993 | Web.of.Science | Web of Science |
| Chinese (T) | 1992 | Web.of.Science | Web of Science |
| Chinese (T) | 1991 | Web.of.Science | Web of Science |
| Chinese (T) | 1990 | Web.of.Science | Web of Science |
| Chinese (T) | 1989 | Web.of.Science | Web of Science |
| Chinese (T) | 1988 | Web.of.Science | Web of Science |
| Chinese (T) | 1987 | Web.of.Science | Web of Science |
| Chinese (T) | 1986 | Web.of.Science | Web of Science |
| Chinese (T) | 1985 | Web.of.Science | Web of Science |
| Chinese (T) | 1984 | Web.of.Science | Web of Science |
| Chinese (T) | 1983 | Web.of.Science | Web of Science |
| Chinese (T) | 1982 | Web.of.Science | Web of Science |
| Chinese (T) | 1981 | Web.of.Science | Web of Science |
| Chinese (T) | 1980 | Web.of.Science | Web of Science |
| Dutch | 2018 | Google.Scholar | Google Scholar |
| Dutch | 2017 | Google.Scholar | Google Scholar |
| Dutch | 2016 | Google.Scholar | Google Scholar |
| Dutch | 2015 | Google.Scholar | Google Scholar |
| Dutch | 2014 | Google.Scholar | Google Scholar |
| Dutch | 2013 | Google.Scholar | Google Scholar |
| Dutch | 2012 | Google.Scholar | Google Scholar |
| Dutch | 2011 | Google.Scholar | Google Scholar |
| Dutch | 2010 | Google.Scholar | Google Scholar |
| Dutch | 2009 | Google.Scholar | Google Scholar |
| Dutch | 2008 | Google.Scholar | Google Scholar |
| Dutch | 2007 | Google.Scholar | Google Scholar |
| Dutch | 2006 | Google.Scholar | Google Scholar |
| Dutch | 2005 | Google.Scholar | Google Scholar |
| Dutch | 2004 | Google.Scholar | Google Scholar |
| Dutch | 2003 | Google.Scholar | Google Scholar |
| Dutch | 2002 | Google.Scholar | Google Scholar |
| Dutch | 2001 | Google.Scholar | Google Scholar |
| Dutch | 2000 | Google.Scholar | Google Scholar |
| Dutch | 1999 | Google.Scholar | Google Scholar |
| Dutch | 1998 | Google.Scholar | Google Scholar |
| Dutch | 1997 | Google.Scholar | Google Scholar |
| Dutch | 1996 | Google.Scholar | Google Scholar |
| Dutch | 1995 | Google.Scholar | Google Scholar |
| Dutch | 1994 | Google.Scholar | Google Scholar |
| Dutch | 1993 | Google.Scholar | Google Scholar |
| Dutch | 1992 | Google.Scholar | Google Scholar |
| Dutch | 1991 | Google.Scholar | Google Scholar |
| Dutch | 1990 | Google.Scholar | Google Scholar |
| Dutch | 1989 | Google.Scholar | Google Scholar |
| Dutch | 1988 | Google.Scholar | Google Scholar |
| Dutch | 1987 | Google.Scholar | Google Scholar |
| Dutch | 1986 | Google.Scholar | Google Scholar |
| Dutch | 1985 | Google.Scholar | Google Scholar |
| Dutch | 1984 | Google.Scholar | Google Scholar |
| Dutch | 1983 | Google.Scholar | Google Scholar |
| Dutch | 1982 | Google.Scholar | Google Scholar |
| Dutch | 1981 | Google.Scholar | Google Scholar |
| Dutch | 1980 | Google.Scholar | Google Scholar |
| Dutch | 2018 | Narcis | Local |
| Dutch | 2017 | Narcis | Local |
| Dutch | 2016 | Narcis | Local |
| Dutch | 2015 | Narcis | Local |
| Dutch | 2014 | Narcis | Local |
| Dutch | 2013 | Narcis | Local |
| Dutch | 2012 | Narcis | Local |
| Dutch | 2011 | Narcis | Local |
| Dutch | 2010 | Narcis | Local |
| Dutch | 2009 | Narcis | Local |
| Dutch | 2008 | Narcis | Local |
| Dutch | 2007 | Narcis | Local |
| Dutch | 2006 | Narcis | Local |
| Dutch | 2005 | Narcis | Local |
| Dutch | 2004 | Narcis | Local |
| Dutch | 2003 | Narcis | Local |
| Dutch | 2002 | Narcis | Local |
| Dutch | 2001 | Narcis | Local |
| Dutch | 2000 | Narcis | Local |
| Dutch | 1999 | Narcis | Local |
| Dutch | 1998 | Narcis | Local |
| Dutch | 1997 | Narcis | Local |
| Dutch | 1996 | Narcis | Local |
| Dutch | 1995 | Narcis | Local |
| Dutch | 1994 | Narcis | Local |
| Dutch | 1993 | Narcis | Local |
| Dutch | 1992 | Narcis | Local |
| Dutch | 1991 | Narcis | Local |
| Dutch | 1990 | Narcis | Local |
| Dutch | 1989 | Narcis | Local |
| Dutch | 1988 | Narcis | Local |
| Dutch | 1987 | Narcis | Local |
| Dutch | 1986 | Narcis | Local |
| Dutch | 1985 | Narcis | Local |
| Dutch | 1984 | Narcis | Local |
| Dutch | 1983 | Narcis | Local |
| Dutch | 1982 | Narcis | Local |
| Dutch | 1981 | Narcis | Local |
| Dutch | 1980 | Narcis | Local |
| Dutch | 2018 | Scopus | Scopus |
| Dutch | 2017 | Scopus | Scopus |
| Dutch | 2016 | Scopus | Scopus |
| Dutch | 2015 | Scopus | Scopus |
| Dutch | 2014 | Scopus | Scopus |
| Dutch | 2013 | Scopus | Scopus |
| Dutch | 2012 | Scopus | Scopus |
| Dutch | 2011 | Scopus | Scopus |
| Dutch | 2010 | Scopus | Scopus |
| Dutch | 2009 | Scopus | Scopus |
| Dutch | 2008 | Scopus | Scopus |
| Dutch | 2007 | Scopus | Scopus |
| Dutch | 2006 | Scopus | Scopus |
| Dutch | 2005 | Scopus | Scopus |
| Dutch | 2004 | Scopus | Scopus |
| Dutch | 2003 | Scopus | Scopus |
| Dutch | 2002 | Scopus | Scopus |
| Dutch | 2001 | Scopus | Scopus |
| Dutch | 2000 | Scopus | Scopus |
| Dutch | 1999 | Scopus | Scopus |
| Dutch | 1998 | Scopus | Scopus |
| Dutch | 1997 | Scopus | Scopus |
| Dutch | 1996 | Scopus | Scopus |
| Dutch | 1995 | Scopus | Scopus |
| Dutch | 1994 | Scopus | Scopus |
| Dutch | 1993 | Scopus | Scopus |
| Dutch | 1992 | Scopus | Scopus |
| Dutch | 1991 | Scopus | Scopus |
| Dutch | 1990 | Scopus | Scopus |
| Dutch | 1989 | Scopus | Scopus |
| Dutch | 1988 | Scopus | Scopus |
| Dutch | 1987 | Scopus | Scopus |
| Dutch | 1986 | Scopus | Scopus |
| Dutch | 1985 | Scopus | Scopus |
| Dutch | 1984 | Scopus | Scopus |
| Dutch | 1983 | Scopus | Scopus |
| Dutch | 1982 | Scopus | Scopus |
| Dutch | 1981 | Scopus | Scopus |
| Dutch | 1980 | Scopus | Scopus |
| Dutch | 2018 | Web.of.Science | Web of Science |
| Dutch | 2017 | Web.of.Science | Web of Science |
| Dutch | 2016 | Web.of.Science | Web of Science |
| Dutch | 2015 | Web.of.Science | Web of Science |
| Dutch | 2014 | Web.of.Science | Web of Science |
| Dutch | 2013 | Web.of.Science | Web of Science |
| Dutch | 2012 | Web.of.Science | Web of Science |
| Dutch | 2011 | Web.of.Science | Web of Science |
| Dutch | 2010 | Web.of.Science | Web of Science |
| Dutch | 2009 | Web.of.Science | Web of Science |
| Dutch | 2008 | Web.of.Science | Web of Science |
| Dutch | 2007 | Web.of.Science | Web of Science |
| Dutch | 2006 | Web.of.Science | Web of Science |
| Dutch | 2005 | Web.of.Science | Web of Science |
| Dutch | 2004 | Web.of.Science | Web of Science |
| Dutch | 2003 | Web.of.Science | Web of Science |
| Dutch | 2002 | Web.of.Science | Web of Science |
| Dutch | 2001 | Web.of.Science | Web of Science |
| Dutch | 2000 | Web.of.Science | Web of Science |
| Dutch | 1999 | Web.of.Science | Web of Science |
| Dutch | 1998 | Web.of.Science | Web of Science |
| Dutch | 1997 | Web.of.Science | Web of Science |
| Dutch | 1996 | Web.of.Science | Web of Science |
| Dutch | 1995 | Web.of.Science | Web of Science |
| Dutch | 1994 | Web.of.Science | Web of Science |
| Dutch | 1993 | Web.of.Science | Web of Science |
| Dutch | 1992 | Web.of.Science | Web of Science |
| Dutch | 1991 | Web.of.Science | Web of Science |
| Dutch | 1990 | Web.of.Science | Web of Science |
| Dutch | 1989 | Web.of.Science | Web of Science |
| Dutch | 1988 | Web.of.Science | Web of Science |
| Dutch | 1987 | Web.of.Science | Web of Science |
| Dutch | 1986 | Web.of.Science | Web of Science |
| Dutch | 1985 | Web.of.Science | Web of Science |
| Dutch | 1984 | Web.of.Science | Web of Science |
| Dutch | 1983 | Web.of.Science | Web of Science |
| Dutch | 1982 | Web.of.Science | Web of Science |
| Dutch | 1981 | Web.of.Science | Web of Science |
| Dutch | 1980 | Web.of.Science | Web of Science |
| English | 2018 | Google.Scholar | Google Scholar |
| English | 2017 | Google.Scholar | Google Scholar |
| English | 2016 | Google.Scholar | Google Scholar |
| English | 2015 | Google.Scholar | Google Scholar |
| English | 2014 | Google.Scholar | Google Scholar |
| English | 2013 | Google.Scholar | Google Scholar |
| English | 2012 | Google.Scholar | Google Scholar |
| English | 2011 | Google.Scholar | Google Scholar |
| English | 2010 | Google.Scholar | Google Scholar |
| English | 2009 | Google.Scholar | Google Scholar |
| English | 2008 | Google.Scholar | Google Scholar |
| English | 2007 | Google.Scholar | Google Scholar |
| English | 2006 | Google.Scholar | Google Scholar |
| English | 2005 | Google.Scholar | Google Scholar |
| English | 2004 | Google.Scholar | Google Scholar |
| English | 2003 | Google.Scholar | Google Scholar |
| English | 2002 | Google.Scholar | Google Scholar |
| English | 2001 | Google.Scholar | Google Scholar |
| English | 2000 | Google.Scholar | Google Scholar |
| English | 1999 | Google.Scholar | Google Scholar |
| English | 1998 | Google.Scholar | Google Scholar |
| English | 1997 | Google.Scholar | Google Scholar |
| English | 1996 | Google.Scholar | Google Scholar |
| English | 1995 | Google.Scholar | Google Scholar |
| English | 1994 | Google.Scholar | Google Scholar |
| English | 1993 | Google.Scholar | Google Scholar |
| English | 1992 | Google.Scholar | Google Scholar |
| English | 1991 | Google.Scholar | Google Scholar |
| English | 1990 | Google.Scholar | Google Scholar |
| English | 1989 | Google.Scholar | Google Scholar |
| English | 1988 | Google.Scholar | Google Scholar |
| English | 1987 | Google.Scholar | Google Scholar |
| English | 1986 | Google.Scholar | Google Scholar |
| English | 1985 | Google.Scholar | Google Scholar |
| English | 1984 | Google.Scholar | Google Scholar |
| English | 1983 | Google.Scholar | Google Scholar |
| English | 1982 | Google.Scholar | Google Scholar |
| English | 1981 | Google.Scholar | Google Scholar |
| English | 1980 | Google.Scholar | Google Scholar |
| English | 2018 | Scopus | Scopus |
| English | 2017 | Scopus | Scopus |
| English | 2016 | Scopus | Scopus |
| English | 2015 | Scopus | Scopus |
| English | 2014 | Scopus | Scopus |
| English | 2013 | Scopus | Scopus |
| English | 2012 | Scopus | Scopus |
| English | 2011 | Scopus | Scopus |
| English | 2010 | Scopus | Scopus |
| English | 2009 | Scopus | Scopus |
| English | 2008 | Scopus | Scopus |
| English | 2007 | Scopus | Scopus |
| English | 2006 | Scopus | Scopus |
| English | 2005 | Scopus | Scopus |
| English | 2004 | Scopus | Scopus |
| English | 2003 | Scopus | Scopus |
| English | 2002 | Scopus | Scopus |
| English | 2001 | Scopus | Scopus |
| English | 2000 | Scopus | Scopus |
| English | 1999 | Scopus | Scopus |
| English | 1998 | Scopus | Scopus |
| English | 1997 | Scopus | Scopus |
| English | 1996 | Scopus | Scopus |
| English | 1995 | Scopus | Scopus |
| English | 1994 | Scopus | Scopus |
| English | 1993 | Scopus | Scopus |
| English | 1992 | Scopus | Scopus |
| English | 1991 | Scopus | Scopus |
| English | 1990 | Scopus | Scopus |
| English | 1989 | Scopus | Scopus |
| English | 1988 | Scopus | Scopus |
| English | 1987 | Scopus | Scopus |
| English | 1986 | Scopus | Scopus |
| English | 1985 | Scopus | Scopus |
| English | 1984 | Scopus | Scopus |
| English | 1983 | Scopus | Scopus |
| English | 1982 | Scopus | Scopus |
| English | 1981 | Scopus | Scopus |
| English | 1980 | Scopus | Scopus |
| English | 2018 | Web.of.Science | Web of Science |
| English | 2017 | Web.of.Science | Web of Science |
| English | 2016 | Web.of.Science | Web of Science |
| English | 2015 | Web.of.Science | Web of Science |
| English | 2014 | Web.of.Science | Web of Science |
| English | 2013 | Web.of.Science | Web of Science |
| English | 2012 | Web.of.Science | Web of Science |
| English | 2011 | Web.of.Science | Web of Science |
| English | 2010 | Web.of.Science | Web of Science |
| English | 2009 | Web.of.Science | Web of Science |
| English | 2008 | Web.of.Science | Web of Science |
| English | 2007 | Web.of.Science | Web of Science |
| English | 2006 | Web.of.Science | Web of Science |
| English | 2005 | Web.of.Science | Web of Science |
| English | 2004 | Web.of.Science | Web of Science |
| English | 2003 | Web.of.Science | Web of Science |
| English | 2002 | Web.of.Science | Web of Science |
| English | 2001 | Web.of.Science | Web of Science |
| English | 2000 | Web.of.Science | Web of Science |
| English | 1999 | Web.of.Science | Web of Science |
| English | 1998 | Web.of.Science | Web of Science |
| English | 1997 | Web.of.Science | Web of Science |
| English | 1996 | Web.of.Science | Web of Science |
| English | 1995 | Web.of.Science | Web of Science |
| English | 1994 | Web.of.Science | Web of Science |
| English | 1993 | Web.of.Science | Web of Science |
| English | 1992 | Web.of.Science | Web of Science |
| English | 1991 | Web.of.Science | Web of Science |
| English | 1990 | Web.of.Science | Web of Science |
| English | 1989 | Web.of.Science | Web of Science |
| English | 1988 | Web.of.Science | Web of Science |
| English | 1987 | Web.of.Science | Web of Science |
| English | 1986 | Web.of.Science | Web of Science |
| English | 1985 | Web.of.Science | Web of Science |
| English | 1984 | Web.of.Science | Web of Science |
| English | 1983 | Web.of.Science | Web of Science |
| English | 1982 | Web.of.Science | Web of Science |
| English | 1981 | Web.of.Science | Web of Science |
| English | 1980 | Web.of.Science | Web of Science |
| French | 2018 | Google.Scholar | Google Scholar |
| French | 2017 | Google.Scholar | Google Scholar |
| French | 2016 | Google.Scholar | Google Scholar |
| French | 2015 | Google.Scholar | Google Scholar |
| French | 2014 | Google.Scholar | Google Scholar |
| French | 2013 | Google.Scholar | Google Scholar |
| French | 2012 | Google.Scholar | Google Scholar |
| French | 2011 | Google.Scholar | Google Scholar |
| French | 2010 | Google.Scholar | Google Scholar |
| French | 2009 | Google.Scholar | Google Scholar |
| French | 2008 | Google.Scholar | Google Scholar |
| French | 2007 | Google.Scholar | Google Scholar |
| French | 2006 | Google.Scholar | Google Scholar |
| French | 2005 | Google.Scholar | Google Scholar |
| French | 2004 | Google.Scholar | Google Scholar |
| French | 2003 | Google.Scholar | Google Scholar |
| French | 2002 | Google.Scholar | Google Scholar |
| French | 2001 | Google.Scholar | Google Scholar |
| French | 2000 | Google.Scholar | Google Scholar |
| French | 1999 | Google.Scholar | Google Scholar |
| French | 1998 | Google.Scholar | Google Scholar |
| French | 1997 | Google.Scholar | Google Scholar |
| French | 1996 | Google.Scholar | Google Scholar |
| French | 1995 | Google.Scholar | Google Scholar |
| French | 1994 | Google.Scholar | Google Scholar |
| French | 1993 | Google.Scholar | Google Scholar |
| French | 1992 | Google.Scholar | Google Scholar |
| French | 1991 | Google.Scholar | Google Scholar |
| French | 1990 | Google.Scholar | Google Scholar |
| French | 1989 | Google.Scholar | Google Scholar |
| French | 1988 | Google.Scholar | Google Scholar |
| French | 1987 | Google.Scholar | Google Scholar |
| French | 1986 | Google.Scholar | Google Scholar |
| French | 1985 | Google.Scholar | Google Scholar |
| French | 1984 | Google.Scholar | Google Scholar |
| French | 1983 | Google.Scholar | Google Scholar |
| French | 1982 | Google.Scholar | Google Scholar |
| French | 1981 | Google.Scholar | Google Scholar |
| French | 1980 | Google.Scholar | Google Scholar |
| French | 2018 | Persee | Local |
| French | 2017 | Persee | Local |
| French | 2016 | Persee | Local |
| French | 2015 | Persee | Local |
| French | 2014 | Persee | Local |
| French | 2013 | Persee | Local |
| French | 2012 | Persee | Local |
| French | 2011 | Persee | Local |
| French | 2010 | Persee | Local |
| French | 2009 | Persee | Local |
| French | 2008 | Persee | Local |
| French | 2007 | Persee | Local |
| French | 2006 | Persee | Local |
| French | 2005 | Persee | Local |
| French | 2004 | Persee | Local |
| French | 2003 | Persee | Local |
| French | 2002 | Persee | Local |
| French | 2001 | Persee | Local |
| French | 2000 | Persee | Local |
| French | 1999 | Persee | Local |
| French | 1998 | Persee | Local |
| French | 1997 | Persee | Local |
| French | 1996 | Persee | Local |
| French | 1995 | Persee | Local |
| French | 1994 | Persee | Local |
| French | 1993 | Persee | Local |
| French | 1992 | Persee | Local |
| French | 1991 | Persee | Local |
| French | 1990 | Persee | Local |
| French | 1989 | Persee | Local |
| French | 1988 | Persee | Local |
| French | 1987 | Persee | Local |
| French | 1986 | Persee | Local |
| French | 1985 | Persee | Local |
| French | 1984 | Persee | Local |
| French | 1983 | Persee | Local |
| French | 1982 | Persee | Local |
| French | 1981 | Persee | Local |
| French | 1980 | Persee | Local |
| French | 2018 | Scopus | Scopus |
| French | 2017 | Scopus | Scopus |
| French | 2016 | Scopus | Scopus |
| French | 2015 | Scopus | Scopus |
| French | 2014 | Scopus | Scopus |
| French | 2013 | Scopus | Scopus |
| French | 2012 | Scopus | Scopus |
| French | 2011 | Scopus | Scopus |
| French | 2010 | Scopus | Scopus |
| French | 2009 | Scopus | Scopus |
| French | 2008 | Scopus | Scopus |
| French | 2007 | Scopus | Scopus |
| French | 2006 | Scopus | Scopus |
| French | 2005 | Scopus | Scopus |
| French | 2004 | Scopus | Scopus |
| French | 2003 | Scopus | Scopus |
| French | 2002 | Scopus | Scopus |
| French | 2001 | Scopus | Scopus |
| French | 2000 | Scopus | Scopus |
| French | 1999 | Scopus | Scopus |
| French | 1998 | Scopus | Scopus |
| French | 1997 | Scopus | Scopus |
| French | 1996 | Scopus | Scopus |
| French | 1995 | Scopus | Scopus |
| French | 1994 | Scopus | Scopus |
| French | 1993 | Scopus | Scopus |
| French | 1992 | Scopus | Scopus |
| French | 1991 | Scopus | Scopus |
| French | 1990 | Scopus | Scopus |
| French | 1989 | Scopus | Scopus |
| French | 1988 | Scopus | Scopus |
| French | 1987 | Scopus | Scopus |
| French | 1986 | Scopus | Scopus |
| French | 1985 | Scopus | Scopus |
| French | 1984 | Scopus | Scopus |
| French | 1983 | Scopus | Scopus |
| French | 1982 | Scopus | Scopus |
| French | 1981 | Scopus | Scopus |
| French | 1980 | Scopus | Scopus |
| French | 2018 | Web.of.Science | Web of Science |
| French | 2017 | Web.of.Science | Web of Science |
| French | 2016 | Web.of.Science | Web of Science |
| French | 2015 | Web.of.Science | Web of Science |
| French | 2014 | Web.of.Science | Web of Science |
| French | 2013 | Web.of.Science | Web of Science |
| French | 2012 | Web.of.Science | Web of Science |
| French | 2011 | Web.of.Science | Web of Science |
| French | 2010 | Web.of.Science | Web of Science |
| French | 2009 | Web.of.Science | Web of Science |
| French | 2008 | Web.of.Science | Web of Science |
| French | 2007 | Web.of.Science | Web of Science |
| French | 2006 | Web.of.Science | Web of Science |
| French | 2005 | Web.of.Science | Web of Science |
| French | 2004 | Web.of.Science | Web of Science |
| French | 2003 | Web.of.Science | Web of Science |
| French | 2002 | Web.of.Science | Web of Science |
| French | 2001 | Web.of.Science | Web of Science |
| French | 2000 | Web.of.Science | Web of Science |
| French | 1999 | Web.of.Science | Web of Science |
| French | 1998 | Web.of.Science | Web of Science |
| French | 1997 | Web.of.Science | Web of Science |
| French | 1996 | Web.of.Science | Web of Science |
| French | 1995 | Web.of.Science | Web of Science |
| French | 1994 | Web.of.Science | Web of Science |
| French | 1993 | Web.of.Science | Web of Science |
| French | 1992 | Web.of.Science | Web of Science |
| French | 1991 | Web.of.Science | Web of Science |
| French | 1990 | Web.of.Science | Web of Science |
| French | 1989 | Web.of.Science | Web of Science |
| French | 1988 | Web.of.Science | Web of Science |
| French | 1987 | Web.of.Science | Web of Science |
| French | 1986 | Web.of.Science | Web of Science |
| French | 1985 | Web.of.Science | Web of Science |
| French | 1984 | Web.of.Science | Web of Science |
| French | 1983 | Web.of.Science | Web of Science |
| French | 1982 | Web.of.Science | Web of Science |
| French | 1981 | Web.of.Science | Web of Science |
| French | 1980 | Web.of.Science | Web of Science |
| German | 2018 | BASE | Local |
| German | 2017 | BASE | Local |
| German | 2016 | BASE | Local |
| German | 2015 | BASE | Local |
| German | 2014 | BASE | Local |
| German | 2013 | BASE | Local |
| German | 2012 | BASE | Local |
| German | 2011 | BASE | Local |
| German | 2010 | BASE | Local |
| German | 2009 | BASE | Local |
| German | 2008 | BASE | Local |
| German | 2007 | BASE | Local |
| German | 2006 | BASE | Local |
| German | 2005 | BASE | Local |
| German | 2004 | BASE | Local |
| German | 2003 | BASE | Local |
| German | 2002 | BASE | Local |
| German | 2001 | BASE | Local |
| German | 2000 | BASE | Local |
| German | 1999 | BASE | Local |
| German | 1998 | BASE | Local |
| German | 1997 | BASE | Local |
| German | 1996 | BASE | Local |
| German | 1995 | BASE | Local |
| German | 1994 | BASE | Local |
| German | 1993 | BASE | Local |
| German | 1992 | BASE | Local |
| German | 1991 | BASE | Local |
| German | 1990 | BASE | Local |
| German | 1989 | BASE | Local |
| German | 1988 | BASE | Local |
| German | 1987 | BASE | Local |
| German | 1986 | BASE | Local |
| German | 1985 | BASE | Local |
| German | 1984 | BASE | Local |
| German | 1983 | BASE | Local |
| German | 1982 | BASE | Local |
| German | 1981 | BASE | Local |
| German | 1980 | BASE | Local |
| German | 2018 | Google.Scholar | Google Scholar |
| German | 2017 | Google.Scholar | Google Scholar |
| German | 2016 | Google.Scholar | Google Scholar |
| German | 2015 | Google.Scholar | Google Scholar |
| German | 2014 | Google.Scholar | Google Scholar |
| German | 2013 | Google.Scholar | Google Scholar |
| German | 2012 | Google.Scholar | Google Scholar |
| German | 2011 | Google.Scholar | Google Scholar |
| German | 2010 | Google.Scholar | Google Scholar |
| German | 2009 | Google.Scholar | Google Scholar |
| German | 2008 | Google.Scholar | Google Scholar |
| German | 2007 | Google.Scholar | Google Scholar |
| German | 2006 | Google.Scholar | Google Scholar |
| German | 2005 | Google.Scholar | Google Scholar |
| German | 2004 | Google.Scholar | Google Scholar |
| German | 2003 | Google.Scholar | Google Scholar |
| German | 2002 | Google.Scholar | Google Scholar |
| German | 2001 | Google.Scholar | Google Scholar |
| German | 2000 | Google.Scholar | Google Scholar |
| German | 1999 | Google.Scholar | Google Scholar |
| German | 1998 | Google.Scholar | Google Scholar |
| German | 1997 | Google.Scholar | Google Scholar |
| German | 1996 | Google.Scholar | Google Scholar |
| German | 1995 | Google.Scholar | Google Scholar |
| German | 1994 | Google.Scholar | Google Scholar |
| German | 1993 | Google.Scholar | Google Scholar |
| German | 1992 | Google.Scholar | Google Scholar |
| German | 1991 | Google.Scholar | Google Scholar |
| German | 1990 | Google.Scholar | Google Scholar |
| German | 1989 | Google.Scholar | Google Scholar |
| German | 1988 | Google.Scholar | Google Scholar |
| German | 1987 | Google.Scholar | Google Scholar |
| German | 1986 | Google.Scholar | Google Scholar |
| German | 1985 | Google.Scholar | Google Scholar |
| German | 1984 | Google.Scholar | Google Scholar |
| German | 1983 | Google.Scholar | Google Scholar |
| German | 1982 | Google.Scholar | Google Scholar |
| German | 1981 | Google.Scholar | Google Scholar |
| German | 1980 | Google.Scholar | Google Scholar |
| German | 2018 | Scopus | Scopus |
| German | 2017 | Scopus | Scopus |
| German | 2016 | Scopus | Scopus |
| German | 2015 | Scopus | Scopus |
| German | 2014 | Scopus | Scopus |
| German | 2013 | Scopus | Scopus |
| German | 2012 | Scopus | Scopus |
| German | 2011 | Scopus | Scopus |
| German | 2010 | Scopus | Scopus |
| German | 2009 | Scopus | Scopus |
| German | 2008 | Scopus | Scopus |
| German | 2007 | Scopus | Scopus |
| German | 2006 | Scopus | Scopus |
| German | 2005 | Scopus | Scopus |
| German | 2004 | Scopus | Scopus |
| German | 2003 | Scopus | Scopus |
| German | 2002 | Scopus | Scopus |
| German | 2001 | Scopus | Scopus |
| German | 2000 | Scopus | Scopus |
| German | 1999 | Scopus | Scopus |
| German | 1998 | Scopus | Scopus |
| German | 1997 | Scopus | Scopus |
| German | 1996 | Scopus | Scopus |
| German | 1995 | Scopus | Scopus |
| German | 1994 | Scopus | Scopus |
| German | 1993 | Scopus | Scopus |
| German | 1992 | Scopus | Scopus |
| German | 1991 | Scopus | Scopus |
| German | 1990 | Scopus | Scopus |
| German | 1989 | Scopus | Scopus |
| German | 1988 | Scopus | Scopus |
| German | 1987 | Scopus | Scopus |
| German | 1986 | Scopus | Scopus |
| German | 1985 | Scopus | Scopus |
| German | 1984 | Scopus | Scopus |
| German | 1983 | Scopus | Scopus |
| German | 1982 | Scopus | Scopus |
| German | 1981 | Scopus | Scopus |
| German | 1980 | Scopus | Scopus |
| German | 2018 | Web.of.Science | Web of Science |
| German | 2017 | Web.of.Science | Web of Science |
| German | 2016 | Web.of.Science | Web of Science |
| German | 2015 | Web.of.Science | Web of Science |
| German | 2014 | Web.of.Science | Web of Science |
| German | 2013 | Web.of.Science | Web of Science |
| German | 2012 | Web.of.Science | Web of Science |
| German | 2011 | Web.of.Science | Web of Science |
| German | 2010 | Web.of.Science | Web of Science |
| German | 2009 | Web.of.Science | Web of Science |
| German | 2008 | Web.of.Science | Web of Science |
| German | 2007 | Web.of.Science | Web of Science |
| German | 2006 | Web.of.Science | Web of Science |
| German | 2005 | Web.of.Science | Web of Science |
| German | 2004 | Web.of.Science | Web of Science |
| German | 2003 | Web.of.Science | Web of Science |
| German | 2002 | Web.of.Science | Web of Science |
| German | 2001 | Web.of.Science | Web of Science |
| German | 2000 | Web.of.Science | Web of Science |
| German | 1999 | Web.of.Science | Web of Science |
| German | 1998 | Web.of.Science | Web of Science |
| German | 1997 | Web.of.Science | Web of Science |
| German | 1996 | Web.of.Science | Web of Science |
| German | 1995 | Web.of.Science | Web of Science |
| German | 1994 | Web.of.Science | Web of Science |
| German | 1993 | Web.of.Science | Web of Science |
| German | 1992 | Web.of.Science | Web of Science |
| German | 1991 | Web.of.Science | Web of Science |
| German | 1990 | Web.of.Science | Web of Science |
| German | 1989 | Web.of.Science | Web of Science |
| German | 1988 | Web.of.Science | Web of Science |
| German | 1987 | Web.of.Science | Web of Science |
| German | 1986 | Web.of.Science | Web of Science |
| German | 1985 | Web.of.Science | Web of Science |
| German | 1984 | Web.of.Science | Web of Science |
| German | 1983 | Web.of.Science | Web of Science |
| German | 1982 | Web.of.Science | Web of Science |
| German | 1981 | Web.of.Science | Web of Science |
| German | 1980 | Web.of.Science | Web of Science |
| Italian | 2018 | Google.Scholar | Google Scholar |
| Italian | 2017 | Google.Scholar | Google Scholar |
| Italian | 2016 | Google.Scholar | Google Scholar |
| Italian | 2015 | Google.Scholar | Google Scholar |
| Italian | 2014 | Google.Scholar | Google Scholar |
| Italian | 2013 | Google.Scholar | Google Scholar |
| Italian | 2012 | Google.Scholar | Google Scholar |
| Italian | 2011 | Google.Scholar | Google Scholar |
| Italian | 2010 | Google.Scholar | Google Scholar |
| Italian | 2009 | Google.Scholar | Google Scholar |
| Italian | 2008 | Google.Scholar | Google Scholar |
| Italian | 2007 | Google.Scholar | Google Scholar |
| Italian | 2006 | Google.Scholar | Google Scholar |
| Italian | 2005 | Google.Scholar | Google Scholar |
| Italian | 2004 | Google.Scholar | Google Scholar |
| Italian | 2003 | Google.Scholar | Google Scholar |
| Italian | 2002 | Google.Scholar | Google Scholar |
| Italian | 2001 | Google.Scholar | Google Scholar |
| Italian | 2000 | Google.Scholar | Google Scholar |
| Italian | 1999 | Google.Scholar | Google Scholar |
| Italian | 1998 | Google.Scholar | Google Scholar |
| Italian | 1997 | Google.Scholar | Google Scholar |
| Italian | 1996 | Google.Scholar | Google Scholar |
| Italian | 1995 | Google.Scholar | Google Scholar |
| Italian | 1994 | Google.Scholar | Google Scholar |
| Italian | 1993 | Google.Scholar | Google Scholar |
| Italian | 1992 | Google.Scholar | Google Scholar |
| Italian | 1991 | Google.Scholar | Google Scholar |
| Italian | 1990 | Google.Scholar | Google Scholar |
| Italian | 1989 | Google.Scholar | Google Scholar |
| Italian | 1988 | Google.Scholar | Google Scholar |
| Italian | 1987 | Google.Scholar | Google Scholar |
| Italian | 1986 | Google.Scholar | Google Scholar |
| Italian | 1985 | Google.Scholar | Google Scholar |
| Italian | 1984 | Google.Scholar | Google Scholar |
| Italian | 1983 | Google.Scholar | Google Scholar |
| Italian | 1982 | Google.Scholar | Google Scholar |
| Italian | 1981 | Google.Scholar | Google Scholar |
| Italian | 1980 | Google.Scholar | Google Scholar |
| Italian | 2018 | Scopus | Scopus |
| Italian | 2017 | Scopus | Scopus |
| Italian | 2016 | Scopus | Scopus |
| Italian | 2015 | Scopus | Scopus |
| Italian | 2014 | Scopus | Scopus |
| Italian | 2013 | Scopus | Scopus |
| Italian | 2012 | Scopus | Scopus |
| Italian | 2011 | Scopus | Scopus |
| Italian | 2010 | Scopus | Scopus |
| Italian | 2009 | Scopus | Scopus |
| Italian | 2008 | Scopus | Scopus |
| Italian | 2007 | Scopus | Scopus |
| Italian | 2006 | Scopus | Scopus |
| Italian | 2005 | Scopus | Scopus |
| Italian | 2004 | Scopus | Scopus |
| Italian | 2003 | Scopus | Scopus |
| Italian | 2002 | Scopus | Scopus |
| Italian | 2001 | Scopus | Scopus |
| Italian | 2000 | Scopus | Scopus |
| Italian | 1999 | Scopus | Scopus |
| Italian | 1998 | Scopus | Scopus |
| Italian | 1997 | Scopus | Scopus |
| Italian | 1996 | Scopus | Scopus |
| Italian | 1995 | Scopus | Scopus |
| Italian | 1994 | Scopus | Scopus |
| Italian | 1993 | Scopus | Scopus |
| Italian | 1992 | Scopus | Scopus |
| Italian | 1991 | Scopus | Scopus |
| Italian | 1990 | Scopus | Scopus |
| Italian | 1989 | Scopus | Scopus |
| Italian | 1988 | Scopus | Scopus |
| Italian | 1987 | Scopus | Scopus |
| Italian | 1986 | Scopus | Scopus |
| Italian | 1985 | Scopus | Scopus |
| Italian | 1984 | Scopus | Scopus |
| Italian | 1983 | Scopus | Scopus |
| Italian | 1982 | Scopus | Scopus |
| Italian | 1981 | Scopus | Scopus |
| Italian | 1980 | Scopus | Scopus |
| Italian | 2018 | Web.of.Science | Web of Science |
| Italian | 2017 | Web.of.Science | Web of Science |
| Italian | 2016 | Web.of.Science | Web of Science |
| Italian | 2015 | Web.of.Science | Web of Science |
| Italian | 2014 | Web.of.Science | Web of Science |
| Italian | 2013 | Web.of.Science | Web of Science |
| Italian | 2012 | Web.of.Science | Web of Science |
| Italian | 2011 | Web.of.Science | Web of Science |
| Italian | 2010 | Web.of.Science | Web of Science |
| Italian | 2009 | Web.of.Science | Web of Science |
| Italian | 2008 | Web.of.Science | Web of Science |
| Italian | 2007 | Web.of.Science | Web of Science |
| Italian | 2006 | Web.of.Science | Web of Science |
| Italian | 2005 | Web.of.Science | Web of Science |
| Italian | 2004 | Web.of.Science | Web of Science |
| Italian | 2003 | Web.of.Science | Web of Science |
| Italian | 2002 | Web.of.Science | Web of Science |
| Italian | 2001 | Web.of.Science | Web of Science |
| Italian | 2000 | Web.of.Science | Web of Science |
| Italian | 1999 | Web.of.Science | Web of Science |
| Italian | 1998 | Web.of.Science | Web of Science |
| Italian | 1997 | Web.of.Science | Web of Science |
| Italian | 1996 | Web.of.Science | Web of Science |
| Italian | 1995 | Web.of.Science | Web of Science |
| Italian | 1994 | Web.of.Science | Web of Science |
| Italian | 1993 | Web.of.Science | Web of Science |
| Italian | 1992 | Web.of.Science | Web of Science |
| Italian | 1991 | Web.of.Science | Web of Science |
| Italian | 1990 | Web.of.Science | Web of Science |
| Italian | 1989 | Web.of.Science | Web of Science |
| Italian | 1988 | Web.of.Science | Web of Science |
| Italian | 1987 | Web.of.Science | Web of Science |
| Italian | 1986 | Web.of.Science | Web of Science |
| Italian | 1985 | Web.of.Science | Web of Science |
| Italian | 1984 | Web.of.Science | Web of Science |
| Italian | 1983 | Web.of.Science | Web of Science |
| Italian | 1982 | Web.of.Science | Web of Science |
| Italian | 1981 | Web.of.Science | Web of Science |
| Italian | 1980 | Web.of.Science | Web of Science |
| Japanese | 2018 | Google.Scholar | Google Scholar |
| Japanese | 2017 | Google.Scholar | Google Scholar |
| Japanese | 2016 | Google.Scholar | Google Scholar |
| Japanese | 2015 | Google.Scholar | Google Scholar |
| Japanese | 2014 | Google.Scholar | Google Scholar |
| Japanese | 2013 | Google.Scholar | Google Scholar |
| Japanese | 2012 | Google.Scholar | Google Scholar |
| Japanese | 2011 | Google.Scholar | Google Scholar |
| Japanese | 2010 | Google.Scholar | Google Scholar |
| Japanese | 2009 | Google.Scholar | Google Scholar |
| Japanese | 2008 | Google.Scholar | Google Scholar |
| Japanese | 2007 | Google.Scholar | Google Scholar |
| Japanese | 2006 | Google.Scholar | Google Scholar |
| Japanese | 2005 | Google.Scholar | Google Scholar |
| Japanese | 2004 | Google.Scholar | Google Scholar |
| Japanese | 2003 | Google.Scholar | Google Scholar |
| Japanese | 2002 | Google.Scholar | Google Scholar |
| Japanese | 2001 | Google.Scholar | Google Scholar |
| Japanese | 2000 | Google.Scholar | Google Scholar |
| Japanese | 1999 | Google.Scholar | Google Scholar |
| Japanese | 1998 | Google.Scholar | Google Scholar |
| Japanese | 1997 | Google.Scholar | Google Scholar |
| Japanese | 1996 | Google.Scholar | Google Scholar |
| Japanese | 1995 | Google.Scholar | Google Scholar |
| Japanese | 1994 | Google.Scholar | Google Scholar |
| Japanese | 1993 | Google.Scholar | Google Scholar |
| Japanese | 1992 | Google.Scholar | Google Scholar |
| Japanese | 1991 | Google.Scholar | Google Scholar |
| Japanese | 1990 | Google.Scholar | Google Scholar |
| Japanese | 1989 | Google.Scholar | Google Scholar |
| Japanese | 1988 | Google.Scholar | Google Scholar |
| Japanese | 1987 | Google.Scholar | Google Scholar |
| Japanese | 1986 | Google.Scholar | Google Scholar |
| Japanese | 1985 | Google.Scholar | Google Scholar |
| Japanese | 1984 | Google.Scholar | Google Scholar |
| Japanese | 1983 | Google.Scholar | Google Scholar |
| Japanese | 1982 | Google.Scholar | Google Scholar |
| Japanese | 1981 | Google.Scholar | Google Scholar |
| Japanese | 1980 | Google.Scholar | Google Scholar |
| Japanese | 2018 | J-stage | Local |
| Japanese | 2017 | J-stage | Local |
| Japanese | 2016 | J-stage | Local |
| Japanese | 2015 | J-stage | Local |
| Japanese | 2014 | J-stage | Local |
| Japanese | 2013 | J-stage | Local |
| Japanese | 2012 | J-stage | Local |
| Japanese | 2011 | J-stage | Local |
| Japanese | 2010 | J-stage | Local |
| Japanese | 2009 | J-stage | Local |
| Japanese | 2008 | J-stage | Local |
| Japanese | 2007 | J-stage | Local |
| Japanese | 2006 | J-stage | Local |
| Japanese | 2005 | J-stage | Local |
| Japanese | 2004 | J-stage | Local |
| Japanese | 2003 | J-stage | Local |
| Japanese | 2002 | J-stage | Local |
| Japanese | 2001 | J-stage | Local |
| Japanese | 2000 | J-stage | Local |
| Japanese | 1999 | J-stage | Local |
| Japanese | 1998 | J-stage | Local |
| Japanese | 1997 | J-stage | Local |
| Japanese | 1996 | J-stage | Local |
| Japanese | 1995 | J-stage | Local |
| Japanese | 1994 | J-stage | Local |
| Japanese | 1993 | J-stage | Local |
| Japanese | 1992 | J-stage | Local |
| Japanese | 1991 | J-stage | Local |
| Japanese | 1990 | J-stage | Local |
| Japanese | 1989 | J-stage | Local |
| Japanese | 1988 | J-stage | Local |
| Japanese | 1987 | J-stage | Local |
| Japanese | 1986 | J-stage | Local |
| Japanese | 1985 | J-stage | Local |
| Japanese | 1984 | J-stage | Local |
| Japanese | 1983 | J-stage | Local |
| Japanese | 1982 | J-stage | Local |
| Japanese | 1981 | J-stage | Local |
| Japanese | 1980 | J-stage | Local |
| Japanese | 2018 | Scopus | Scopus |
| Japanese | 2017 | Scopus | Scopus |
| Japanese | 2016 | Scopus | Scopus |
| Japanese | 2015 | Scopus | Scopus |
| Japanese | 2014 | Scopus | Scopus |
| Japanese | 2013 | Scopus | Scopus |
| Japanese | 2012 | Scopus | Scopus |
| Japanese | 2011 | Scopus | Scopus |
| Japanese | 2010 | Scopus | Scopus |
| Japanese | 2009 | Scopus | Scopus |
| Japanese | 2008 | Scopus | Scopus |
| Japanese | 2007 | Scopus | Scopus |
| Japanese | 2006 | Scopus | Scopus |
| Japanese | 2005 | Scopus | Scopus |
| Japanese | 2004 | Scopus | Scopus |
| Japanese | 2003 | Scopus | Scopus |
| Japanese | 2002 | Scopus | Scopus |
| Japanese | 2001 | Scopus | Scopus |
| Japanese | 2000 | Scopus | Scopus |
| Japanese | 1999 | Scopus | Scopus |
| Japanese | 1998 | Scopus | Scopus |
| Japanese | 1997 | Scopus | Scopus |
| Japanese | 1996 | Scopus | Scopus |
| Japanese | 1995 | Scopus | Scopus |
| Japanese | 1994 | Scopus | Scopus |
| Japanese | 1993 | Scopus | Scopus |
| Japanese | 1992 | Scopus | Scopus |
| Japanese | 1991 | Scopus | Scopus |
| Japanese | 1990 | Scopus | Scopus |
| Japanese | 1989 | Scopus | Scopus |
| Japanese | 1988 | Scopus | Scopus |
| Japanese | 1987 | Scopus | Scopus |
| Japanese | 1986 | Scopus | Scopus |
| Japanese | 1985 | Scopus | Scopus |
| Japanese | 1984 | Scopus | Scopus |
| Japanese | 1983 | Scopus | Scopus |
| Japanese | 1982 | Scopus | Scopus |
| Japanese | 1981 | Scopus | Scopus |
| Japanese | 1980 | Scopus | Scopus |
| Japanese | 2018 | Web.of.Science | Web of Science |
| Japanese | 2017 | Web.of.Science | Web of Science |
| Japanese | 2016 | Web.of.Science | Web of Science |
| Japanese | 2015 | Web.of.Science | Web of Science |
| Japanese | 2014 | Web.of.Science | Web of Science |
| Japanese | 2013 | Web.of.Science | Web of Science |
| Japanese | 2012 | Web.of.Science | Web of Science |
| Japanese | 2011 | Web.of.Science | Web of Science |
| Japanese | 2010 | Web.of.Science | Web of Science |
| Japanese | 2009 | Web.of.Science | Web of Science |
| Japanese | 2008 | Web.of.Science | Web of Science |
| Japanese | 2007 | Web.of.Science | Web of Science |
| Japanese | 2006 | Web.of.Science | Web of Science |
| Japanese | 2005 | Web.of.Science | Web of Science |
| Japanese | 2004 | Web.of.Science | Web of Science |
| Japanese | 2003 | Web.of.Science | Web of Science |
| Japanese | 2002 | Web.of.Science | Web of Science |
| Japanese | 2001 | Web.of.Science | Web of Science |
| Japanese | 2000 | Web.of.Science | Web of Science |
| Japanese | 1999 | Web.of.Science | Web of Science |
| Japanese | 1998 | Web.of.Science | Web of Science |
| Japanese | 1997 | Web.of.Science | Web of Science |
| Japanese | 1996 | Web.of.Science | Web of Science |
| Japanese | 1995 | Web.of.Science | Web of Science |
| Japanese | 1994 | Web.of.Science | Web of Science |
| Japanese | 1993 | Web.of.Science | Web of Science |
| Japanese | 1992 | Web.of.Science | Web of Science |
| Japanese | 1991 | Web.of.Science | Web of Science |
| Japanese | 1990 | Web.of.Science | Web of Science |
| Japanese | 1989 | Web.of.Science | Web of Science |
| Japanese | 1988 | Web.of.Science | Web of Science |
| Japanese | 1987 | Web.of.Science | Web of Science |
| Japanese | 1986 | Web.of.Science | Web of Science |
| Japanese | 1985 | Web.of.Science | Web of Science |
| Japanese | 1984 | Web.of.Science | Web of Science |
| Japanese | 1983 | Web.of.Science | Web of Science |
| Japanese | 1982 | Web.of.Science | Web of Science |
| Japanese | 1981 | Web.of.Science | Web of Science |
| Japanese | 1980 | Web.of.Science | Web of Science |
| Korean | 2018 | Google.Scholar | Google Scholar |
| Korean | 2017 | Google.Scholar | Google Scholar |
| Korean | 2016 | Google.Scholar | Google Scholar |
| Korean | 2015 | Google.Scholar | Google Scholar |
| Korean | 2014 | Google.Scholar | Google Scholar |
| Korean | 2013 | Google.Scholar | Google Scholar |
| Korean | 2012 | Google.Scholar | Google Scholar |
| Korean | 2011 | Google.Scholar | Google Scholar |
| Korean | 2010 | Google.Scholar | Google Scholar |
| Korean | 2009 | Google.Scholar | Google Scholar |
| Korean | 2008 | Google.Scholar | Google Scholar |
| Korean | 2007 | Google.Scholar | Google Scholar |
| Korean | 2006 | Google.Scholar | Google Scholar |
| Korean | 2005 | Google.Scholar | Google Scholar |
| Korean | 2004 | Google.Scholar | Google Scholar |
| Korean | 2003 | Google.Scholar | Google Scholar |
| Korean | 2002 | Google.Scholar | Google Scholar |
| Korean | 2001 | Google.Scholar | Google Scholar |
| Korean | 2000 | Google.Scholar | Google Scholar |
| Korean | 1999 | Google.Scholar | Google Scholar |
| Korean | 1998 | Google.Scholar | Google Scholar |
| Korean | 1997 | Google.Scholar | Google Scholar |
| Korean | 1996 | Google.Scholar | Google Scholar |
| Korean | 1995 | Google.Scholar | Google Scholar |
| Korean | 1994 | Google.Scholar | Google Scholar |
| Korean | 1993 | Google.Scholar | Google Scholar |
| Korean | 1992 | Google.Scholar | Google Scholar |
| Korean | 1991 | Google.Scholar | Google Scholar |
| Korean | 1990 | Google.Scholar | Google Scholar |
| Korean | 1989 | Google.Scholar | Google Scholar |
| Korean | 1988 | Google.Scholar | Google Scholar |
| Korean | 1987 | Google.Scholar | Google Scholar |
| Korean | 1986 | Google.Scholar | Google Scholar |
| Korean | 1985 | Google.Scholar | Google Scholar |
| Korean | 1984 | Google.Scholar | Google Scholar |
| Korean | 1983 | Google.Scholar | Google Scholar |
| Korean | 1982 | Google.Scholar | Google Scholar |
| Korean | 1981 | Google.Scholar | Google Scholar |
| Korean | 1980 | Google.Scholar | Google Scholar |
| Korean | 2018 | KCI | Local |
| Korean | 2017 | KCI | Local |
| Korean | 2016 | KCI | Local |
| Korean | 2015 | KCI | Local |
| Korean | 2014 | KCI | Local |
| Korean | 2013 | KCI | Local |
| Korean | 2012 | KCI | Local |
| Korean | 2011 | KCI | Local |
| Korean | 2010 | KCI | Local |
| Korean | 2009 | KCI | Local |
| Korean | 2008 | KCI | Local |
| Korean | 2007 | KCI | Local |
| Korean | 2006 | KCI | Local |
| Korean | 2005 | KCI | Local |
| Korean | 2004 | KCI | Local |
| Korean | 2003 | KCI | Local |
| Korean | 2002 | KCI | Local |
| Korean | 2001 | KCI | Local |
| Korean | 2000 | KCI | Local |
| Korean | 1999 | KCI | Local |
| Korean | 1998 | KCI | Local |
| Korean | 1997 | KCI | Local |
| Korean | 1996 | KCI | Local |
| Korean | 1995 | KCI | Local |
| Korean | 1994 | KCI | Local |
| Korean | 1993 | KCI | Local |
| Korean | 1992 | KCI | Local |
| Korean | 1991 | KCI | Local |
| Korean | 1990 | KCI | Local |
| Korean | 1989 | KCI | Local |
| Korean | 1988 | KCI | Local |
| Korean | 1987 | KCI | Local |
| Korean | 1986 | KCI | Local |
| Korean | 1985 | KCI | Local |
| Korean | 1984 | KCI | Local |
| Korean | 1983 | KCI | Local |
| Korean | 1982 | KCI | Local |
| Korean | 1981 | KCI | Local |
| Korean | 1980 | KCI | Local |
| Korean | 2018 | Scopus | Scopus |
| Korean | 2017 | Scopus | Scopus |
| Korean | 2016 | Scopus | Scopus |
| Korean | 2015 | Scopus | Scopus |
| Korean | 2014 | Scopus | Scopus |
| Korean | 2013 | Scopus | Scopus |
| Korean | 2012 | Scopus | Scopus |
| Korean | 2011 | Scopus | Scopus |
| Korean | 2010 | Scopus | Scopus |
| Korean | 2009 | Scopus | Scopus |
| Korean | 2008 | Scopus | Scopus |
| Korean | 2007 | Scopus | Scopus |
| Korean | 2006 | Scopus | Scopus |
| Korean | 2005 | Scopus | Scopus |
| Korean | 2004 | Scopus | Scopus |
| Korean | 2003 | Scopus | Scopus |
| Korean | 2002 | Scopus | Scopus |
| Korean | 2001 | Scopus | Scopus |
| Korean | 2000 | Scopus | Scopus |
| Korean | 1999 | Scopus | Scopus |
| Korean | 1998 | Scopus | Scopus |
| Korean | 1997 | Scopus | Scopus |
| Korean | 1996 | Scopus | Scopus |
| Korean | 1995 | Scopus | Scopus |
| Korean | 1994 | Scopus | Scopus |
| Korean | 1993 | Scopus | Scopus |
| Korean | 1992 | Scopus | Scopus |
| Korean | 1991 | Scopus | Scopus |
| Korean | 1990 | Scopus | Scopus |
| Korean | 1989 | Scopus | Scopus |
| Korean | 1988 | Scopus | Scopus |
| Korean | 1987 | Scopus | Scopus |
| Korean | 1986 | Scopus | Scopus |
| Korean | 1985 | Scopus | Scopus |
| Korean | 1984 | Scopus | Scopus |
| Korean | 1983 | Scopus | Scopus |
| Korean | 1982 | Scopus | Scopus |
| Korean | 1981 | Scopus | Scopus |
| Korean | 1980 | Scopus | Scopus |
| Korean | 2018 | Web.of.Science | Web of Science |
| Korean | 2017 | Web.of.Science | Web of Science |
| Korean | 2016 | Web.of.Science | Web of Science |
| Korean | 2015 | Web.of.Science | Web of Science |
| Korean | 2014 | Web.of.Science | Web of Science |
| Korean | 2013 | Web.of.Science | Web of Science |
| Korean | 2012 | Web.of.Science | Web of Science |
| Korean | 2011 | Web.of.Science | Web of Science |
| Korean | 2010 | Web.of.Science | Web of Science |
| Korean | 2009 | Web.of.Science | Web of Science |
| Korean | 2008 | Web.of.Science | Web of Science |
| Korean | 2007 | Web.of.Science | Web of Science |
| Korean | 2006 | Web.of.Science | Web of Science |
| Korean | 2005 | Web.of.Science | Web of Science |
| Korean | 2004 | Web.of.Science | Web of Science |
| Korean | 2003 | Web.of.Science | Web of Science |
| Korean | 2002 | Web.of.Science | Web of Science |
| Korean | 2001 | Web.of.Science | Web of Science |
| Korean | 2000 | Web.of.Science | Web of Science |
| Korean | 1999 | Web.of.Science | Web of Science |
| Korean | 1998 | Web.of.Science | Web of Science |
| Korean | 1997 | Web.of.Science | Web of Science |
| Korean | 1996 | Web.of.Science | Web of Science |
| Korean | 1995 | Web.of.Science | Web of Science |
| Korean | 1994 | Web.of.Science | Web of Science |
| Korean | 1993 | Web.of.Science | Web of Science |
| Korean | 1992 | Web.of.Science | Web of Science |
| Korean | 1991 | Web.of.Science | Web of Science |
| Korean | 1990 | Web.of.Science | Web of Science |
| Korean | 1989 | Web.of.Science | Web of Science |
| Korean | 1988 | Web.of.Science | Web of Science |
| Korean | 1987 | Web.of.Science | Web of Science |
| Korean | 1986 | Web.of.Science | Web of Science |
| Korean | 1985 | Web.of.Science | Web of Science |
| Korean | 1984 | Web.of.Science | Web of Science |
| Korean | 1983 | Web.of.Science | Web of Science |
| Korean | 1982 | Web.of.Science | Web of Science |
| Korean | 1981 | Web.of.Science | Web of Science |
| Korean | 1980 | Web.of.Science | Web of Science |
| Persian | 2018 | Google.Scholar | Google Scholar |
| Persian | 2017 | Google.Scholar | Google Scholar |
| Persian | 2016 | Google.Scholar | Google Scholar |
| Persian | 2015 | Google.Scholar | Google Scholar |
| Persian | 2014 | Google.Scholar | Google Scholar |
| Persian | 2013 | Google.Scholar | Google Scholar |
| Persian | 2012 | Google.Scholar | Google Scholar |
| Persian | 2011 | Google.Scholar | Google Scholar |
| Persian | 2010 | Google.Scholar | Google Scholar |
| Persian | 2009 | Google.Scholar | Google Scholar |
| Persian | 2008 | Google.Scholar | Google Scholar |
| Persian | 2007 | Google.Scholar | Google Scholar |
| Persian | 2006 | Google.Scholar | Google Scholar |
| Persian | 2005 | Google.Scholar | Google Scholar |
| Persian | 2004 | Google.Scholar | Google Scholar |
| Persian | 2003 | Google.Scholar | Google Scholar |
| Persian | 2002 | Google.Scholar | Google Scholar |
| Persian | 2001 | Google.Scholar | Google Scholar |
| Persian | 2000 | Google.Scholar | Google Scholar |
| Persian | 1999 | Google.Scholar | Google Scholar |
| Persian | 1998 | Google.Scholar | Google Scholar |
| Persian | 1997 | Google.Scholar | Google Scholar |
| Persian | 1996 | Google.Scholar | Google Scholar |
| Persian | 1995 | Google.Scholar | Google Scholar |
| Persian | 1994 | Google.Scholar | Google Scholar |
| Persian | 1993 | Google.Scholar | Google Scholar |
| Persian | 1992 | Google.Scholar | Google Scholar |
| Persian | 1991 | Google.Scholar | Google Scholar |
| Persian | 1990 | Google.Scholar | Google Scholar |
| Persian | 1989 | Google.Scholar | Google Scholar |
| Persian | 1988 | Google.Scholar | Google Scholar |
| Persian | 1987 | Google.Scholar | Google Scholar |
| Persian | 1986 | Google.Scholar | Google Scholar |
| Persian | 1985 | Google.Scholar | Google Scholar |
| Persian | 1984 | Google.Scholar | Google Scholar |
| Persian | 1983 | Google.Scholar | Google Scholar |
| Persian | 1982 | Google.Scholar | Google Scholar |
| Persian | 1981 | Google.Scholar | Google Scholar |
| Persian | 1980 | Google.Scholar | Google Scholar |
| Persian | 2018 | Scopus | Scopus |
| Persian | 2017 | Scopus | Scopus |
| Persian | 2016 | Scopus | Scopus |
| Persian | 2015 | Scopus | Scopus |
| Persian | 2014 | Scopus | Scopus |
| Persian | 2013 | Scopus | Scopus |
| Persian | 2012 | Scopus | Scopus |
| Persian | 2011 | Scopus | Scopus |
| Persian | 2010 | Scopus | Scopus |
| Persian | 2009 | Scopus | Scopus |
| Persian | 2008 | Scopus | Scopus |
| Persian | 2007 | Scopus | Scopus |
| Persian | 2006 | Scopus | Scopus |
| Persian | 2005 | Scopus | Scopus |
| Persian | 2004 | Scopus | Scopus |
| Persian | 2003 | Scopus | Scopus |
| Persian | 2002 | Scopus | Scopus |
| Persian | 2001 | Scopus | Scopus |
| Persian | 2000 | Scopus | Scopus |
| Persian | 1999 | Scopus | Scopus |
| Persian | 1998 | Scopus | Scopus |
| Persian | 1997 | Scopus | Scopus |
| Persian | 1996 | Scopus | Scopus |
| Persian | 1995 | Scopus | Scopus |
| Persian | 1994 | Scopus | Scopus |
| Persian | 1993 | Scopus | Scopus |
| Persian | 1992 | Scopus | Scopus |
| Persian | 1991 | Scopus | Scopus |
| Persian | 1990 | Scopus | Scopus |
| Persian | 1989 | Scopus | Scopus |
| Persian | 1988 | Scopus | Scopus |
| Persian | 1987 | Scopus | Scopus |
| Persian | 1986 | Scopus | Scopus |
| Persian | 1985 | Scopus | Scopus |
| Persian | 1984 | Scopus | Scopus |
| Persian | 1983 | Scopus | Scopus |
| Persian | 1982 | Scopus | Scopus |
| Persian | 1981 | Scopus | Scopus |
| Persian | 1980 | Scopus | Scopus |
| Persian | 2018 | Web.of.Science | Web of Science |
| Persian | 2017 | Web.of.Science | Web of Science |
| Persian | 2016 | Web.of.Science | Web of Science |
| Persian | 2015 | Web.of.Science | Web of Science |
| Persian | 2014 | Web.of.Science | Web of Science |
| Persian | 2013 | Web.of.Science | Web of Science |
| Persian | 2012 | Web.of.Science | Web of Science |
| Persian | 2011 | Web.of.Science | Web of Science |
| Persian | 2010 | Web.of.Science | Web of Science |
| Persian | 2009 | Web.of.Science | Web of Science |
| Persian | 2008 | Web.of.Science | Web of Science |
| Persian | 2007 | Web.of.Science | Web of Science |
| Persian | 2006 | Web.of.Science | Web of Science |
| Persian | 2005 | Web.of.Science | Web of Science |
| Persian | 2004 | Web.of.Science | Web of Science |
| Persian | 2003 | Web.of.Science | Web of Science |
| Persian | 2002 | Web.of.Science | Web of Science |
| Persian | 2001 | Web.of.Science | Web of Science |
| Persian | 2000 | Web.of.Science | Web of Science |
| Persian | 1999 | Web.of.Science | Web of Science |
| Persian | 1998 | Web.of.Science | Web of Science |
| Persian | 1997 | Web.of.Science | Web of Science |
| Persian | 1996 | Web.of.Science | Web of Science |
| Persian | 1995 | Web.of.Science | Web of Science |
| Persian | 1994 | Web.of.Science | Web of Science |
| Persian | 1993 | Web.of.Science | Web of Science |
| Persian | 1992 | Web.of.Science | Web of Science |
| Persian | 1991 | Web.of.Science | Web of Science |
| Persian | 1990 | Web.of.Science | Web of Science |
| Persian | 1989 | Web.of.Science | Web of Science |
| Persian | 1988 | Web.of.Science | Web of Science |
| Persian | 1987 | Web.of.Science | Web of Science |
| Persian | 1986 | Web.of.Science | Web of Science |
| Persian | 1985 | Web.of.Science | Web of Science |
| Persian | 1984 | Web.of.Science | Web of Science |
| Persian | 1983 | Web.of.Science | Web of Science |
| Persian | 1982 | Web.of.Science | Web of Science |
| Persian | 1981 | Web.of.Science | Web of Science |
| Persian | 1980 | Web.of.Science | Web of Science |
| Polish | 2018 | Google.Scholar | Google Scholar |
| Polish | 2017 | Google.Scholar | Google Scholar |
| Polish | 2016 | Google.Scholar | Google Scholar |
| Polish | 2015 | Google.Scholar | Google Scholar |
| Polish | 2014 | Google.Scholar | Google Scholar |
| Polish | 2013 | Google.Scholar | Google Scholar |
| Polish | 2012 | Google.Scholar | Google Scholar |
| Polish | 2011 | Google.Scholar | Google Scholar |
| Polish | 2010 | Google.Scholar | Google Scholar |
| Polish | 2009 | Google.Scholar | Google Scholar |
| Polish | 2008 | Google.Scholar | Google Scholar |
| Polish | 2007 | Google.Scholar | Google Scholar |
| Polish | 2006 | Google.Scholar | Google Scholar |
| Polish | 2005 | Google.Scholar | Google Scholar |
| Polish | 2004 | Google.Scholar | Google Scholar |
| Polish | 2003 | Google.Scholar | Google Scholar |
| Polish | 2002 | Google.Scholar | Google Scholar |
| Polish | 2001 | Google.Scholar | Google Scholar |
| Polish | 2000 | Google.Scholar | Google Scholar |
| Polish | 1999 | Google.Scholar | Google Scholar |
| Polish | 1998 | Google.Scholar | Google Scholar |
| Polish | 1997 | Google.Scholar | Google Scholar |
| Polish | 1996 | Google.Scholar | Google Scholar |
| Polish | 1995 | Google.Scholar | Google Scholar |
| Polish | 1994 | Google.Scholar | Google Scholar |
| Polish | 1993 | Google.Scholar | Google Scholar |
| Polish | 1992 | Google.Scholar | Google Scholar |
| Polish | 1991 | Google.Scholar | Google Scholar |
| Polish | 1990 | Google.Scholar | Google Scholar |
| Polish | 1989 | Google.Scholar | Google Scholar |
| Polish | 1988 | Google.Scholar | Google Scholar |
| Polish | 1987 | Google.Scholar | Google Scholar |
| Polish | 1986 | Google.Scholar | Google Scholar |
| Polish | 1985 | Google.Scholar | Google Scholar |
| Polish | 1984 | Google.Scholar | Google Scholar |
| Polish | 1983 | Google.Scholar | Google Scholar |
| Polish | 1982 | Google.Scholar | Google Scholar |
| Polish | 1981 | Google.Scholar | Google Scholar |
| Polish | 1980 | Google.Scholar | Google Scholar |
| Polish | 2018 | PBN | Local |
| Polish | 2017 | PBN | Local |
| Polish | 2016 | PBN | Local |
| Polish | 2015 | PBN | Local |
| Polish | 2014 | PBN | Local |
| Polish | 2013 | PBN | Local |
| Polish | 2012 | PBN | Local |
| Polish | 2011 | PBN | Local |
| Polish | 2010 | PBN | Local |
| Polish | 2009 | PBN | Local |
| Polish | 2008 | PBN | Local |
| Polish | 2007 | PBN | Local |
| Polish | 2006 | PBN | Local |
| Polish | 2005 | PBN | Local |
| Polish | 2004 | PBN | Local |
| Polish | 2003 | PBN | Local |
| Polish | 2002 | PBN | Local |
| Polish | 2001 | PBN | Local |
| Polish | 2000 | PBN | Local |
| Polish | 1999 | PBN | Local |
| Polish | 1998 | PBN | Local |
| Polish | 1997 | PBN | Local |
| Polish | 1996 | PBN | Local |
| Polish | 1995 | PBN | Local |
| Polish | 1994 | PBN | Local |
| Polish | 1993 | PBN | Local |
| Polish | 1992 | PBN | Local |
| Polish | 1991 | PBN | Local |
| Polish | 1990 | PBN | Local |
| Polish | 1989 | PBN | Local |
| Polish | 1988 | PBN | Local |
| Polish | 1987 | PBN | Local |
| Polish | 1986 | PBN | Local |
| Polish | 1985 | PBN | Local |
| Polish | 1984 | PBN | Local |
| Polish | 1983 | PBN | Local |
| Polish | 1982 | PBN | Local |
| Polish | 1981 | PBN | Local |
| Polish | 1980 | PBN | Local |
| Polish | 2018 | Scopus | Scopus |
| Polish | 2017 | Scopus | Scopus |
| Polish | 2016 | Scopus | Scopus |
| Polish | 2015 | Scopus | Scopus |
| Polish | 2014 | Scopus | Scopus |
| Polish | 2013 | Scopus | Scopus |
| Polish | 2012 | Scopus | Scopus |
| Polish | 2011 | Scopus | Scopus |
| Polish | 2010 | Scopus | Scopus |
| Polish | 2009 | Scopus | Scopus |
| Polish | 2008 | Scopus | Scopus |
| Polish | 2007 | Scopus | Scopus |
| Polish | 2006 | Scopus | Scopus |
| Polish | 2005 | Scopus | Scopus |
| Polish | 2004 | Scopus | Scopus |
| Polish | 2003 | Scopus | Scopus |
| Polish | 2002 | Scopus | Scopus |
| Polish | 2001 | Scopus | Scopus |
| Polish | 2000 | Scopus | Scopus |
| Polish | 1999 | Scopus | Scopus |
| Polish | 1998 | Scopus | Scopus |
| Polish | 1997 | Scopus | Scopus |
| Polish | 1996 | Scopus | Scopus |
| Polish | 1995 | Scopus | Scopus |
| Polish | 1994 | Scopus | Scopus |
| Polish | 1993 | Scopus | Scopus |
| Polish | 1992 | Scopus | Scopus |
| Polish | 1991 | Scopus | Scopus |
| Polish | 1990 | Scopus | Scopus |
| Polish | 1989 | Scopus | Scopus |
| Polish | 1988 | Scopus | Scopus |
| Polish | 1987 | Scopus | Scopus |
| Polish | 1986 | Scopus | Scopus |
| Polish | 1985 | Scopus | Scopus |
| Polish | 1984 | Scopus | Scopus |
| Polish | 1983 | Scopus | Scopus |
| Polish | 1982 | Scopus | Scopus |
| Polish | 1981 | Scopus | Scopus |
| Polish | 1980 | Scopus | Scopus |
| Polish | 2018 | Web.of.Science | Web of Science |
| Polish | 2017 | Web.of.Science | Web of Science |
| Polish | 2016 | Web.of.Science | Web of Science |
| Polish | 2015 | Web.of.Science | Web of Science |
| Polish | 2014 | Web.of.Science | Web of Science |
| Polish | 2013 | Web.of.Science | Web of Science |
| Polish | 2012 | Web.of.Science | Web of Science |
| Polish | 2011 | Web.of.Science | Web of Science |
| Polish | 2010 | Web.of.Science | Web of Science |
| Polish | 2009 | Web.of.Science | Web of Science |
| Polish | 2008 | Web.of.Science | Web of Science |
| Polish | 2007 | Web.of.Science | Web of Science |
| Polish | 2006 | Web.of.Science | Web of Science |
| Polish | 2005 | Web.of.Science | Web of Science |
| Polish | 2004 | Web.of.Science | Web of Science |
| Polish | 2003 | Web.of.Science | Web of Science |
| Polish | 2002 | Web.of.Science | Web of Science |
| Polish | 2001 | Web.of.Science | Web of Science |
| Polish | 2000 | Web.of.Science | Web of Science |
| Polish | 1999 | Web.of.Science | Web of Science |
| Polish | 1998 | Web.of.Science | Web of Science |
| Polish | 1997 | Web.of.Science | Web of Science |
| Polish | 1996 | Web.of.Science | Web of Science |
| Polish | 1995 | Web.of.Science | Web of Science |
| Polish | 1994 | Web.of.Science | Web of Science |
| Polish | 1993 | Web.of.Science | Web of Science |
| Polish | 1992 | Web.of.Science | Web of Science |
| Polish | 1991 | Web.of.Science | Web of Science |
| Polish | 1990 | Web.of.Science | Web of Science |
| Polish | 1989 | Web.of.Science | Web of Science |
| Polish | 1988 | Web.of.Science | Web of Science |
| Polish | 1987 | Web.of.Science | Web of Science |
| Polish | 1986 | Web.of.Science | Web of Science |
| Polish | 1985 | Web.of.Science | Web of Science |
| Polish | 1984 | Web.of.Science | Web of Science |
| Polish | 1983 | Web.of.Science | Web of Science |
| Polish | 1982 | Web.of.Science | Web of Science |
| Polish | 1981 | Web.of.Science | Web of Science |
| Polish | 1980 | Web.of.Science | Web of Science |
| Portuguese | 2018 | Google.Scholar | Google Scholar |
| Portuguese | 2017 | Google.Scholar | Google Scholar |
| Portuguese | 2016 | Google.Scholar | Google Scholar |
| Portuguese | 2015 | Google.Scholar | Google Scholar |
| Portuguese | 2014 | Google.Scholar | Google Scholar |
| Portuguese | 2013 | Google.Scholar | Google Scholar |
| Portuguese | 2012 | Google.Scholar | Google Scholar |
| Portuguese | 2011 | Google.Scholar | Google Scholar |
| Portuguese | 2010 | Google.Scholar | Google Scholar |
| Portuguese | 2009 | Google.Scholar | Google Scholar |
| Portuguese | 2008 | Google.Scholar | Google Scholar |
| Portuguese | 2007 | Google.Scholar | Google Scholar |
| Portuguese | 2006 | Google.Scholar | Google Scholar |
| Portuguese | 2005 | Google.Scholar | Google Scholar |
| Portuguese | 2004 | Google.Scholar | Google Scholar |
| Portuguese | 2003 | Google.Scholar | Google Scholar |
| Portuguese | 2002 | Google.Scholar | Google Scholar |
| Portuguese | 2001 | Google.Scholar | Google Scholar |
| Portuguese | 2000 | Google.Scholar | Google Scholar |
| Portuguese | 1999 | Google.Scholar | Google Scholar |
| Portuguese | 1998 | Google.Scholar | Google Scholar |
| Portuguese | 1997 | Google.Scholar | Google Scholar |
| Portuguese | 1996 | Google.Scholar | Google Scholar |
| Portuguese | 1995 | Google.Scholar | Google Scholar |
| Portuguese | 1994 | Google.Scholar | Google Scholar |
| Portuguese | 1993 | Google.Scholar | Google Scholar |
| Portuguese | 1992 | Google.Scholar | Google Scholar |
| Portuguese | 1991 | Google.Scholar | Google Scholar |
| Portuguese | 1990 | Google.Scholar | Google Scholar |
| Portuguese | 1989 | Google.Scholar | Google Scholar |
| Portuguese | 1988 | Google.Scholar | Google Scholar |
| Portuguese | 1987 | Google.Scholar | Google Scholar |
| Portuguese | 1986 | Google.Scholar | Google Scholar |
| Portuguese | 1985 | Google.Scholar | Google Scholar |
| Portuguese | 1984 | Google.Scholar | Google Scholar |
| Portuguese | 1983 | Google.Scholar | Google Scholar |
| Portuguese | 1982 | Google.Scholar | Google Scholar |
| Portuguese | 1981 | Google.Scholar | Google Scholar |
| Portuguese | 1980 | Google.Scholar | Google Scholar |
| Portuguese | 2018 | SciELO | Local |
| Portuguese | 2017 | SciELO | Local |
| Portuguese | 2016 | SciELO | Local |
| Portuguese | 2015 | SciELO | Local |
| Portuguese | 2014 | SciELO | Local |
| Portuguese | 2013 | SciELO | Local |
| Portuguese | 2012 | SciELO | Local |
| Portuguese | 2011 | SciELO | Local |
| Portuguese | 2010 | SciELO | Local |
| Portuguese | 2009 | SciELO | Local |
| Portuguese | 2008 | SciELO | Local |
| Portuguese | 2007 | SciELO | Local |
| Portuguese | 2006 | SciELO | Local |
| Portuguese | 2005 | SciELO | Local |
| Portuguese | 2004 | SciELO | Local |
| Portuguese | 2003 | SciELO | Local |
| Portuguese | 2002 | SciELO | Local |
| Portuguese | 2001 | SciELO | Local |
| Portuguese | 2000 | SciELO | Local |
| Portuguese | 1999 | SciELO | Local |
| Portuguese | 1998 | SciELO | Local |
| Portuguese | 1997 | SciELO | Local |
| Portuguese | 1996 | SciELO | Local |
| Portuguese | 1995 | SciELO | Local |
| Portuguese | 1994 | SciELO | Local |
| Portuguese | 1993 | SciELO | Local |
| Portuguese | 1992 | SciELO | Local |
| Portuguese | 1991 | SciELO | Local |
| Portuguese | 1990 | SciELO | Local |
| Portuguese | 1989 | SciELO | Local |
| Portuguese | 1988 | SciELO | Local |
| Portuguese | 1987 | SciELO | Local |
| Portuguese | 1986 | SciELO | Local |
| Portuguese | 1985 | SciELO | Local |
| Portuguese | 1984 | SciELO | Local |
| Portuguese | 1983 | SciELO | Local |
| Portuguese | 1982 | SciELO | Local |
| Portuguese | 1981 | SciELO | Local |
| Portuguese | 1980 | SciELO | Local |
| Portuguese | 2018 | Scopus | Scopus |
| Portuguese | 2017 | Scopus | Scopus |
| Portuguese | 2016 | Scopus | Scopus |
| Portuguese | 2015 | Scopus | Scopus |
| Portuguese | 2014 | Scopus | Scopus |
| Portuguese | 2013 | Scopus | Scopus |
| Portuguese | 2012 | Scopus | Scopus |
| Portuguese | 2011 | Scopus | Scopus |
| Portuguese | 2010 | Scopus | Scopus |
| Portuguese | 2009 | Scopus | Scopus |
| Portuguese | 2008 | Scopus | Scopus |
| Portuguese | 2007 | Scopus | Scopus |
| Portuguese | 2006 | Scopus | Scopus |
| Portuguese | 2005 | Scopus | Scopus |
| Portuguese | 2004 | Scopus | Scopus |
| Portuguese | 2003 | Scopus | Scopus |
| Portuguese | 2002 | Scopus | Scopus |
| Portuguese | 2001 | Scopus | Scopus |
| Portuguese | 2000 | Scopus | Scopus |
| Portuguese | 1999 | Scopus | Scopus |
| Portuguese | 1998 | Scopus | Scopus |
| Portuguese | 1997 | Scopus | Scopus |
| Portuguese | 1996 | Scopus | Scopus |
| Portuguese | 1995 | Scopus | Scopus |
| Portuguese | 1994 | Scopus | Scopus |
| Portuguese | 1993 | Scopus | Scopus |
| Portuguese | 1992 | Scopus | Scopus |
| Portuguese | 1991 | Scopus | Scopus |
| Portuguese | 1990 | Scopus | Scopus |
| Portuguese | 1989 | Scopus | Scopus |
| Portuguese | 1988 | Scopus | Scopus |
| Portuguese | 1987 | Scopus | Scopus |
| Portuguese | 1986 | Scopus | Scopus |
| Portuguese | 1985 | Scopus | Scopus |
| Portuguese | 1984 | Scopus | Scopus |
| Portuguese | 1983 | Scopus | Scopus |
| Portuguese | 1982 | Scopus | Scopus |
| Portuguese | 1981 | Scopus | Scopus |
| Portuguese | 1980 | Scopus | Scopus |
| Portuguese | 2018 | Web.of.Science | Web of Science |
| Portuguese | 2017 | Web.of.Science | Web of Science |
| Portuguese | 2016 | Web.of.Science | Web of Science |
| Portuguese | 2015 | Web.of.Science | Web of Science |
| Portuguese | 2014 | Web.of.Science | Web of Science |
| Portuguese | 2013 | Web.of.Science | Web of Science |
| Portuguese | 2012 | Web.of.Science | Web of Science |
| Portuguese | 2011 | Web.of.Science | Web of Science |
| Portuguese | 2010 | Web.of.Science | Web of Science |
| Portuguese | 2009 | Web.of.Science | Web of Science |
| Portuguese | 2008 | Web.of.Science | Web of Science |
| Portuguese | 2007 | Web.of.Science | Web of Science |
| Portuguese | 2006 | Web.of.Science | Web of Science |
| Portuguese | 2005 | Web.of.Science | Web of Science |
| Portuguese | 2004 | Web.of.Science | Web of Science |
| Portuguese | 2003 | Web.of.Science | Web of Science |
| Portuguese | 2002 | Web.of.Science | Web of Science |
| Portuguese | 2001 | Web.of.Science | Web of Science |
| Portuguese | 2000 | Web.of.Science | Web of Science |
| Portuguese | 1999 | Web.of.Science | Web of Science |
| Portuguese | 1998 | Web.of.Science | Web of Science |
| Portuguese | 1997 | Web.of.Science | Web of Science |
| Portuguese | 1996 | Web.of.Science | Web of Science |
| Portuguese | 1995 | Web.of.Science | Web of Science |
| Portuguese | 1994 | Web.of.Science | Web of Science |
| Portuguese | 1993 | Web.of.Science | Web of Science |
| Portuguese | 1992 | Web.of.Science | Web of Science |
| Portuguese | 1991 | Web.of.Science | Web of Science |
| Portuguese | 1990 | Web.of.Science | Web of Science |
| Portuguese | 1989 | Web.of.Science | Web of Science |
| Portuguese | 1988 | Web.of.Science | Web of Science |
| Portuguese | 1987 | Web.of.Science | Web of Science |
| Portuguese | 1986 | Web.of.Science | Web of Science |
| Portuguese | 1985 | Web.of.Science | Web of Science |
| Portuguese | 1984 | Web.of.Science | Web of Science |
| Portuguese | 1983 | Web.of.Science | Web of Science |
| Portuguese | 1982 | Web.of.Science | Web of Science |
| Portuguese | 1981 | Web.of.Science | Web of Science |
| Portuguese | 1980 | Web.of.Science | Web of Science |
| Russian | 2018 | Elibrary | Local |
| Russian | 2017 | Elibrary | Local |
| Russian | 2016 | Elibrary | Local |
| Russian | 2015 | Elibrary | Local |
| Russian | 2014 | Elibrary | Local |
| Russian | 2013 | Elibrary | Local |
| Russian | 2012 | Elibrary | Local |
| Russian | 2011 | Elibrary | Local |
| Russian | 2010 | Elibrary | Local |
| Russian | 2009 | Elibrary | Local |
| Russian | 2008 | Elibrary | Local |
| Russian | 2007 | Elibrary | Local |
| Russian | 2006 | Elibrary | Local |
| Russian | 2005 | Elibrary | Local |
| Russian | 2004 | Elibrary | Local |
| Russian | 2003 | Elibrary | Local |
| Russian | 2002 | Elibrary | Local |
| Russian | 2001 | Elibrary | Local |
| Russian | 2000 | Elibrary | Local |
| Russian | 1999 | Elibrary | Local |
| Russian | 1998 | Elibrary | Local |
| Russian | 1997 | Elibrary | Local |
| Russian | 1996 | Elibrary | Local |
| Russian | 1995 | Elibrary | Local |
| Russian | 1994 | Elibrary | Local |
| Russian | 1993 | Elibrary | Local |
| Russian | 1992 | Elibrary | Local |
| Russian | 1991 | Elibrary | Local |
| Russian | 1990 | Elibrary | Local |
| Russian | 1989 | Elibrary | Local |
| Russian | 1988 | Elibrary | Local |
| Russian | 1987 | Elibrary | Local |
| Russian | 1986 | Elibrary | Local |
| Russian | 1985 | Elibrary | Local |
| Russian | 1984 | Elibrary | Local |
| Russian | 1983 | Elibrary | Local |
| Russian | 1982 | Elibrary | Local |
| Russian | 1981 | Elibrary | Local |
| Russian | 1980 | Elibrary | Local |
| Russian | 2018 | Google.Scholar | Google Scholar |
| Russian | 2017 | Google.Scholar | Google Scholar |
| Russian | 2016 | Google.Scholar | Google Scholar |
| Russian | 2015 | Google.Scholar | Google Scholar |
| Russian | 2014 | Google.Scholar | Google Scholar |
| Russian | 2013 | Google.Scholar | Google Scholar |
| Russian | 2012 | Google.Scholar | Google Scholar |
| Russian | 2011 | Google.Scholar | Google Scholar |
| Russian | 2010 | Google.Scholar | Google Scholar |
| Russian | 2009 | Google.Scholar | Google Scholar |
| Russian | 2008 | Google.Scholar | Google Scholar |
| Russian | 2007 | Google.Scholar | Google Scholar |
| Russian | 2006 | Google.Scholar | Google Scholar |
| Russian | 2005 | Google.Scholar | Google Scholar |
| Russian | 2004 | Google.Scholar | Google Scholar |
| Russian | 2003 | Google.Scholar | Google Scholar |
| Russian | 2002 | Google.Scholar | Google Scholar |
| Russian | 2001 | Google.Scholar | Google Scholar |
| Russian | 2000 | Google.Scholar | Google Scholar |
| Russian | 1999 | Google.Scholar | Google Scholar |
| Russian | 1998 | Google.Scholar | Google Scholar |
| Russian | 1997 | Google.Scholar | Google Scholar |
| Russian | 1996 | Google.Scholar | Google Scholar |
| Russian | 1995 | Google.Scholar | Google Scholar |
| Russian | 1994 | Google.Scholar | Google Scholar |
| Russian | 1993 | Google.Scholar | Google Scholar |
| Russian | 1992 | Google.Scholar | Google Scholar |
| Russian | 1991 | Google.Scholar | Google Scholar |
| Russian | 1990 | Google.Scholar | Google Scholar |
| Russian | 1989 | Google.Scholar | Google Scholar |
| Russian | 1988 | Google.Scholar | Google Scholar |
| Russian | 1987 | Google.Scholar | Google Scholar |
| Russian | 1986 | Google.Scholar | Google Scholar |
| Russian | 1985 | Google.Scholar | Google Scholar |
| Russian | 1984 | Google.Scholar | Google Scholar |
| Russian | 1983 | Google.Scholar | Google Scholar |
| Russian | 1982 | Google.Scholar | Google Scholar |
| Russian | 1981 | Google.Scholar | Google Scholar |
| Russian | 1980 | Google.Scholar | Google Scholar |
| Russian | 2018 | Scopus | Scopus |
| Russian | 2017 | Scopus | Scopus |
| Russian | 2016 | Scopus | Scopus |
| Russian | 2015 | Scopus | Scopus |
| Russian | 2014 | Scopus | Scopus |
| Russian | 2013 | Scopus | Scopus |
| Russian | 2012 | Scopus | Scopus |
| Russian | 2011 | Scopus | Scopus |
| Russian | 2010 | Scopus | Scopus |
| Russian | 2009 | Scopus | Scopus |
| Russian | 2008 | Scopus | Scopus |
| Russian | 2007 | Scopus | Scopus |
| Russian | 2006 | Scopus | Scopus |
| Russian | 2005 | Scopus | Scopus |
| Russian | 2004 | Scopus | Scopus |
| Russian | 2003 | Scopus | Scopus |
| Russian | 2002 | Scopus | Scopus |
| Russian | 2001 | Scopus | Scopus |
| Russian | 2000 | Scopus | Scopus |
| Russian | 1999 | Scopus | Scopus |
| Russian | 1998 | Scopus | Scopus |
| Russian | 1997 | Scopus | Scopus |
| Russian | 1996 | Scopus | Scopus |
| Russian | 1995 | Scopus | Scopus |
| Russian | 1994 | Scopus | Scopus |
| Russian | 1993 | Scopus | Scopus |
| Russian | 1992 | Scopus | Scopus |
| Russian | 1991 | Scopus | Scopus |
| Russian | 1990 | Scopus | Scopus |
| Russian | 1989 | Scopus | Scopus |
| Russian | 1988 | Scopus | Scopus |
| Russian | 1987 | Scopus | Scopus |
| Russian | 1986 | Scopus | Scopus |
| Russian | 1985 | Scopus | Scopus |
| Russian | 1984 | Scopus | Scopus |
| Russian | 1983 | Scopus | Scopus |
| Russian | 1982 | Scopus | Scopus |
| Russian | 1981 | Scopus | Scopus |
| Russian | 1980 | Scopus | Scopus |
| Russian | 2018 | Web.of.Science | Web of Science |
| Russian | 2017 | Web.of.Science | Web of Science |
| Russian | 2016 | Web.of.Science | Web of Science |
| Russian | 2015 | Web.of.Science | Web of Science |
| Russian | 2014 | Web.of.Science | Web of Science |
| Russian | 2013 | Web.of.Science | Web of Science |
| Russian | 2012 | Web.of.Science | Web of Science |
| Russian | 2011 | Web.of.Science | Web of Science |
| Russian | 2010 | Web.of.Science | Web of Science |
| Russian | 2009 | Web.of.Science | Web of Science |
| Russian | 2008 | Web.of.Science | Web of Science |
| Russian | 2007 | Web.of.Science | Web of Science |
| Russian | 2006 | Web.of.Science | Web of Science |
| Russian | 2005 | Web.of.Science | Web of Science |
| Russian | 2004 | Web.of.Science | Web of Science |
| Russian | 2003 | Web.of.Science | Web of Science |
| Russian | 2002 | Web.of.Science | Web of Science |
| Russian | 2001 | Web.of.Science | Web of Science |
| Russian | 2000 | Web.of.Science | Web of Science |
| Russian | 1999 | Web.of.Science | Web of Science |
| Russian | 1998 | Web.of.Science | Web of Science |
| Russian | 1997 | Web.of.Science | Web of Science |
| Russian | 1996 | Web.of.Science | Web of Science |
| Russian | 1995 | Web.of.Science | Web of Science |
| Russian | 1994 | Web.of.Science | Web of Science |
| Russian | 1993 | Web.of.Science | Web of Science |
| Russian | 1992 | Web.of.Science | Web of Science |
| Russian | 1991 | Web.of.Science | Web of Science |
| Russian | 1990 | Web.of.Science | Web of Science |
| Russian | 1989 | Web.of.Science | Web of Science |
| Russian | 1988 | Web.of.Science | Web of Science |
| Russian | 1987 | Web.of.Science | Web of Science |
| Russian | 1986 | Web.of.Science | Web of Science |
| Russian | 1985 | Web.of.Science | Web of Science |
| Russian | 1984 | Web.of.Science | Web of Science |
| Russian | 1983 | Web.of.Science | Web of Science |
| Russian | 1982 | Web.of.Science | Web of Science |
| Russian | 1981 | Web.of.Science | Web of Science |
| Russian | 1980 | Web.of.Science | Web of Science |
| Spanish | 2018 | Google.Scholar | Google Scholar |
| Spanish | 2017 | Google.Scholar | Google Scholar |
| Spanish | 2016 | Google.Scholar | Google Scholar |
| Spanish | 2015 | Google.Scholar | Google Scholar |
| Spanish | 2014 | Google.Scholar | Google Scholar |
| Spanish | 2013 | Google.Scholar | Google Scholar |
| Spanish | 2012 | Google.Scholar | Google Scholar |
| Spanish | 2011 | Google.Scholar | Google Scholar |
| Spanish | 2010 | Google.Scholar | Google Scholar |
| Spanish | 2009 | Google.Scholar | Google Scholar |
| Spanish | 2008 | Google.Scholar | Google Scholar |
| Spanish | 2007 | Google.Scholar | Google Scholar |
| Spanish | 2006 | Google.Scholar | Google Scholar |
| Spanish | 2005 | Google.Scholar | Google Scholar |
| Spanish | 2004 | Google.Scholar | Google Scholar |
| Spanish | 2003 | Google.Scholar | Google Scholar |
| Spanish | 2002 | Google.Scholar | Google Scholar |
| Spanish | 2001 | Google.Scholar | Google Scholar |
| Spanish | 2000 | Google.Scholar | Google Scholar |
| Spanish | 1999 | Google.Scholar | Google Scholar |
| Spanish | 1998 | Google.Scholar | Google Scholar |
| Spanish | 1997 | Google.Scholar | Google Scholar |
| Spanish | 1996 | Google.Scholar | Google Scholar |
| Spanish | 1995 | Google.Scholar | Google Scholar |
| Spanish | 1994 | Google.Scholar | Google Scholar |
| Spanish | 1993 | Google.Scholar | Google Scholar |
| Spanish | 1992 | Google.Scholar | Google Scholar |
| Spanish | 1991 | Google.Scholar | Google Scholar |
| Spanish | 1990 | Google.Scholar | Google Scholar |
| Spanish | 1989 | Google.Scholar | Google Scholar |
| Spanish | 1988 | Google.Scholar | Google Scholar |
| Spanish | 1987 | Google.Scholar | Google Scholar |
| Spanish | 1986 | Google.Scholar | Google Scholar |
| Spanish | 1985 | Google.Scholar | Google Scholar |
| Spanish | 1984 | Google.Scholar | Google Scholar |
| Spanish | 1983 | Google.Scholar | Google Scholar |
| Spanish | 1982 | Google.Scholar | Google Scholar |
| Spanish | 1981 | Google.Scholar | Google Scholar |
| Spanish | 1980 | Google.Scholar | Google Scholar |
| Spanish | 2018 | SciELO | Local |
| Spanish | 2017 | SciELO | Local |
| Spanish | 2016 | SciELO | Local |
| Spanish | 2015 | SciELO | Local |
| Spanish | 2014 | SciELO | Local |
| Spanish | 2013 | SciELO | Local |
| Spanish | 2012 | SciELO | Local |
| Spanish | 2011 | SciELO | Local |
| Spanish | 2010 | SciELO | Local |
| Spanish | 2009 | SciELO | Local |
| Spanish | 2008 | SciELO | Local |
| Spanish | 2007 | SciELO | Local |
| Spanish | 2006 | SciELO | Local |
| Spanish | 2005 | SciELO | Local |
| Spanish | 2004 | SciELO | Local |
| Spanish | 2003 | SciELO | Local |
| Spanish | 2002 | SciELO | Local |
| Spanish | 2001 | SciELO | Local |
| Spanish | 2000 | SciELO | Local |
| Spanish | 1999 | SciELO | Local |
| Spanish | 1998 | SciELO | Local |
| Spanish | 1997 | SciELO | Local |
| Spanish | 1996 | SciELO | Local |
| Spanish | 1995 | SciELO | Local |
| Spanish | 1994 | SciELO | Local |
| Spanish | 1993 | SciELO | Local |
| Spanish | 1992 | SciELO | Local |
| Spanish | 1991 | SciELO | Local |
| Spanish | 1990 | SciELO | Local |
| Spanish | 1989 | SciELO | Local |
| Spanish | 1988 | SciELO | Local |
| Spanish | 1987 | SciELO | Local |
| Spanish | 1986 | SciELO | Local |
| Spanish | 1985 | SciELO | Local |
| Spanish | 1984 | SciELO | Local |
| Spanish | 1983 | SciELO | Local |
| Spanish | 1982 | SciELO | Local |
| Spanish | 1981 | SciELO | Local |
| Spanish | 1980 | SciELO | Local |
| Spanish | 2018 | Scopus | Scopus |
| Spanish | 2017 | Scopus | Scopus |
| Spanish | 2016 | Scopus | Scopus |
| Spanish | 2015 | Scopus | Scopus |
| Spanish | 2014 | Scopus | Scopus |
| Spanish | 2013 | Scopus | Scopus |
| Spanish | 2012 | Scopus | Scopus |
| Spanish | 2011 | Scopus | Scopus |
| Spanish | 2010 | Scopus | Scopus |
| Spanish | 2009 | Scopus | Scopus |
| Spanish | 2008 | Scopus | Scopus |
| Spanish | 2007 | Scopus | Scopus |
| Spanish | 2006 | Scopus | Scopus |
| Spanish | 2005 | Scopus | Scopus |
| Spanish | 2004 | Scopus | Scopus |
| Spanish | 2003 | Scopus | Scopus |
| Spanish | 2002 | Scopus | Scopus |
| Spanish | 2001 | Scopus | Scopus |
| Spanish | 2000 | Scopus | Scopus |
| Spanish | 1999 | Scopus | Scopus |
| Spanish | 1998 | Scopus | Scopus |
| Spanish | 1997 | Scopus | Scopus |
| Spanish | 1996 | Scopus | Scopus |
| Spanish | 1995 | Scopus | Scopus |
| Spanish | 1994 | Scopus | Scopus |
| Spanish | 1993 | Scopus | Scopus |
| Spanish | 1992 | Scopus | Scopus |
| Spanish | 1991 | Scopus | Scopus |
| Spanish | 1990 | Scopus | Scopus |
| Spanish | 1989 | Scopus | Scopus |
| Spanish | 1988 | Scopus | Scopus |
| Spanish | 1987 | Scopus | Scopus |
| Spanish | 1986 | Scopus | Scopus |
| Spanish | 1985 | Scopus | Scopus |
| Spanish | 1984 | Scopus | Scopus |
| Spanish | 1983 | Scopus | Scopus |
| Spanish | 1982 | Scopus | Scopus |
| Spanish | 1981 | Scopus | Scopus |
| Spanish | 1980 | Scopus | Scopus |
| Spanish | 2018 | Web.of.Science | Web of Science |
| Spanish | 2017 | Web.of.Science | Web of Science |
| Spanish | 2016 | Web.of.Science | Web of Science |
| Spanish | 2015 | Web.of.Science | Web of Science |
| Spanish | 2014 | Web.of.Science | Web of Science |
| Spanish | 2013 | Web.of.Science | Web of Science |
| Spanish | 2012 | Web.of.Science | Web of Science |
| Spanish | 2011 | Web.of.Science | Web of Science |
| Spanish | 2010 | Web.of.Science | Web of Science |
| Spanish | 2009 | Web.of.Science | Web of Science |
| Spanish | 2008 | Web.of.Science | Web of Science |
| Spanish | 2007 | Web.of.Science | Web of Science |
| Spanish | 2006 | Web.of.Science | Web of Science |
| Spanish | 2005 | Web.of.Science | Web of Science |
| Spanish | 2004 | Web.of.Science | Web of Science |
| Spanish | 2003 | Web.of.Science | Web of Science |
| Spanish | 2002 | Web.of.Science | Web of Science |
| Spanish | 2001 | Web.of.Science | Web of Science |
| Spanish | 2000 | Web.of.Science | Web of Science |
| Spanish | 1999 | Web.of.Science | Web of Science |
| Spanish | 1998 | Web.of.Science | Web of Science |
| Spanish | 1997 | Web.of.Science | Web of Science |
| Spanish | 1996 | Web.of.Science | Web of Science |
| Spanish | 1995 | Web.of.Science | Web of Science |
| Spanish | 1994 | Web.of.Science | Web of Science |
| Spanish | 1993 | Web.of.Science | Web of Science |
| Spanish | 1992 | Web.of.Science | Web of Science |
| Spanish | 1991 | Web.of.Science | Web of Science |
| Spanish | 1990 | Web.of.Science | Web of Science |
| Spanish | 1989 | Web.of.Science | Web of Science |
| Spanish | 1988 | Web.of.Science | Web of Science |
| Spanish | 1987 | Web.of.Science | Web of Science |
| Spanish | 1986 | Web.of.Science | Web of Science |
| Spanish | 1985 | Web.of.Science | Web of Science |
| Spanish | 1984 | Web.of.Science | Web of Science |
| Spanish | 1983 | Web.of.Science | Web of Science |
| Spanish | 1982 | Web.of.Science | Web of Science |
| Spanish | 1981 | Web.of.Science | Web of Science |
| Spanish | 1980 | Web.of.Science | Web of Science |
| Swedish | 2018 | Google.Scholar | Google Scholar |
| Swedish | 2017 | Google.Scholar | Google Scholar |
| Swedish | 2016 | Google.Scholar | Google Scholar |
| Swedish | 2015 | Google.Scholar | Google Scholar |
| Swedish | 2014 | Google.Scholar | Google Scholar |
| Swedish | 2013 | Google.Scholar | Google Scholar |
| Swedish | 2012 | Google.Scholar | Google Scholar |
| Swedish | 2011 | Google.Scholar | Google Scholar |
| Swedish | 2010 | Google.Scholar | Google Scholar |
| Swedish | 2009 | Google.Scholar | Google Scholar |
| Swedish | 2008 | Google.Scholar | Google Scholar |
| Swedish | 2007 | Google.Scholar | Google Scholar |
| Swedish | 2006 | Google.Scholar | Google Scholar |
| Swedish | 2005 | Google.Scholar | Google Scholar |
| Swedish | 2004 | Google.Scholar | Google Scholar |
| Swedish | 2003 | Google.Scholar | Google Scholar |
| Swedish | 2002 | Google.Scholar | Google Scholar |
| Swedish | 2001 | Google.Scholar | Google Scholar |
| Swedish | 2000 | Google.Scholar | Google Scholar |
| Swedish | 1999 | Google.Scholar | Google Scholar |
| Swedish | 1998 | Google.Scholar | Google Scholar |
| Swedish | 1997 | Google.Scholar | Google Scholar |
| Swedish | 1996 | Google.Scholar | Google Scholar |
| Swedish | 1995 | Google.Scholar | Google Scholar |
| Swedish | 1994 | Google.Scholar | Google Scholar |
| Swedish | 1993 | Google.Scholar | Google Scholar |
| Swedish | 1992 | Google.Scholar | Google Scholar |
| Swedish | 1991 | Google.Scholar | Google Scholar |
| Swedish | 1990 | Google.Scholar | Google Scholar |
| Swedish | 1989 | Google.Scholar | Google Scholar |
| Swedish | 1988 | Google.Scholar | Google Scholar |
| Swedish | 1987 | Google.Scholar | Google Scholar |
| Swedish | 1986 | Google.Scholar | Google Scholar |
| Swedish | 1985 | Google.Scholar | Google Scholar |
| Swedish | 1984 | Google.Scholar | Google Scholar |
| Swedish | 1983 | Google.Scholar | Google Scholar |
| Swedish | 1982 | Google.Scholar | Google Scholar |
| Swedish | 1981 | Google.Scholar | Google Scholar |
| Swedish | 1980 | Google.Scholar | Google Scholar |
| Swedish | 2018 | Scopus | Scopus |
| Swedish | 2017 | Scopus | Scopus |
| Swedish | 2016 | Scopus | Scopus |
| Swedish | 2015 | Scopus | Scopus |
| Swedish | 2014 | Scopus | Scopus |
| Swedish | 2013 | Scopus | Scopus |
| Swedish | 2012 | Scopus | Scopus |
| Swedish | 2011 | Scopus | Scopus |
| Swedish | 2010 | Scopus | Scopus |
| Swedish | 2009 | Scopus | Scopus |
| Swedish | 2008 | Scopus | Scopus |
| Swedish | 2007 | Scopus | Scopus |
| Swedish | 2006 | Scopus | Scopus |
| Swedish | 2005 | Scopus | Scopus |
| Swedish | 2004 | Scopus | Scopus |
| Swedish | 2003 | Scopus | Scopus |
| Swedish | 2002 | Scopus | Scopus |
| Swedish | 2001 | Scopus | Scopus |
| Swedish | 2000 | Scopus | Scopus |
| Swedish | 1999 | Scopus | Scopus |
| Swedish | 1998 | Scopus | Scopus |
| Swedish | 1997 | Scopus | Scopus |
| Swedish | 1996 | Scopus | Scopus |
| Swedish | 1995 | Scopus | Scopus |
| Swedish | 1994 | Scopus | Scopus |
| Swedish | 1993 | Scopus | Scopus |
| Swedish | 1992 | Scopus | Scopus |
| Swedish | 1991 | Scopus | Scopus |
| Swedish | 1990 | Scopus | Scopus |
| Swedish | 1989 | Scopus | Scopus |
| Swedish | 1988 | Scopus | Scopus |
| Swedish | 1987 | Scopus | Scopus |
| Swedish | 1986 | Scopus | Scopus |
| Swedish | 1985 | Scopus | Scopus |
| Swedish | 1984 | Scopus | Scopus |
| Swedish | 1983 | Scopus | Scopus |
| Swedish | 1982 | Scopus | Scopus |
| Swedish | 1981 | Scopus | Scopus |
| Swedish | 1980 | Scopus | Scopus |
| Swedish | 2018 | Web.of.Science | Web of Science |
| Swedish | 2017 | Web.of.Science | Web of Science |
| Swedish | 2016 | Web.of.Science | Web of Science |
| Swedish | 2015 | Web.of.Science | Web of Science |
| Swedish | 2014 | Web.of.Science | Web of Science |
| Swedish | 2013 | Web.of.Science | Web of Science |
| Swedish | 2012 | Web.of.Science | Web of Science |
| Swedish | 2011 | Web.of.Science | Web of Science |
| Swedish | 2010 | Web.of.Science | Web of Science |
| Swedish | 2009 | Web.of.Science | Web of Science |
| Swedish | 2008 | Web.of.Science | Web of Science |
| Swedish | 2007 | Web.of.Science | Web of Science |
| Swedish | 2006 | Web.of.Science | Web of Science |
| Swedish | 2005 | Web.of.Science | Web of Science |
| Swedish | 2004 | Web.of.Science | Web of Science |
| Swedish | 2003 | Web.of.Science | Web of Science |
| Swedish | 2002 | Web.of.Science | Web of Science |
| Swedish | 2001 | Web.of.Science | Web of Science |
| Swedish | 2000 | Web.of.Science | Web of Science |
| Swedish | 1999 | Web.of.Science | Web of Science |
| Swedish | 1998 | Web.of.Science | Web of Science |
| Swedish | 1997 | Web.of.Science | Web of Science |
| Swedish | 1996 | Web.of.Science | Web of Science |
| Swedish | 1995 | Web.of.Science | Web of Science |
| Swedish | 1994 | Web.of.Science | Web of Science |
| Swedish | 1993 | Web.of.Science | Web of Science |
| Swedish | 1992 | Web.of.Science | Web of Science |
| Swedish | 1991 | Web.of.Science | Web of Science |
| Swedish | 1990 | Web.of.Science | Web of Science |
| Swedish | 1989 | Web.of.Science | Web of Science |
| Swedish | 1988 | Web.of.Science | Web of Science |
| Swedish | 1987 | Web.of.Science | Web of Science |
| Swedish | 1986 | Web.of.Science | Web of Science |
| Swedish | 1985 | Web.of.Science | Web of Science |
| Swedish | 1984 | Web.of.Science | Web of Science |
| Swedish | 1983 | Web.of.Science | Web of Science |
| Swedish | 1982 | Web.of.Science | Web of Science |
| Swedish | 1981 | Web.of.Science | Web of Science |
| Swedish | 1980 | Web.of.Science | Web of Science |
| Turkish | 2018 | DergiPark | Local |
| Turkish | 2017 | DergiPark | Local |
| Turkish | 2016 | DergiPark | Local |
| Turkish | 2015 | DergiPark | Local |
| Turkish | 2014 | DergiPark | Local |
| Turkish | 2013 | DergiPark | Local |
| Turkish | 2012 | DergiPark | Local |
| Turkish | 2011 | DergiPark | Local |
| Turkish | 2010 | DergiPark | Local |
| Turkish | 2009 | DergiPark | Local |
| Turkish | 2008 | DergiPark | Local |
| Turkish | 2007 | DergiPark | Local |
| Turkish | 2006 | DergiPark | Local |
| Turkish | 2005 | DergiPark | Local |
| Turkish | 2004 | DergiPark | Local |
| Turkish | 2003 | DergiPark | Local |
| Turkish | 2002 | DergiPark | Local |
| Turkish | 2001 | DergiPark | Local |
| Turkish | 2000 | DergiPark | Local |
| Turkish | 1999 | DergiPark | Local |
| Turkish | 1998 | DergiPark | Local |
| Turkish | 1997 | DergiPark | Local |
| Turkish | 1996 | DergiPark | Local |
| Turkish | 1995 | DergiPark | Local |
| Turkish | 1994 | DergiPark | Local |
| Turkish | 1993 | DergiPark | Local |
| Turkish | 1992 | DergiPark | Local |
| Turkish | 1991 | DergiPark | Local |
| Turkish | 1990 | DergiPark | Local |
| Turkish | 1989 | DergiPark | Local |
| Turkish | 1988 | DergiPark | Local |
| Turkish | 1987 | DergiPark | Local |
| Turkish | 1986 | DergiPark | Local |
| Turkish | 1985 | DergiPark | Local |
| Turkish | 1984 | DergiPark | Local |
| Turkish | 1983 | DergiPark | Local |
| Turkish | 1982 | DergiPark | Local |
| Turkish | 1981 | DergiPark | Local |
| Turkish | 1980 | DergiPark | Local |
| Turkish | 2018 | Google.Scholar | Google Scholar |
| Turkish | 2017 | Google.Scholar | Google Scholar |
| Turkish | 2016 | Google.Scholar | Google Scholar |
| Turkish | 2015 | Google.Scholar | Google Scholar |
| Turkish | 2014 | Google.Scholar | Google Scholar |
| Turkish | 2013 | Google.Scholar | Google Scholar |
| Turkish | 2012 | Google.Scholar | Google Scholar |
| Turkish | 2011 | Google.Scholar | Google Scholar |
| Turkish | 2010 | Google.Scholar | Google Scholar |
| Turkish | 2009 | Google.Scholar | Google Scholar |
| Turkish | 2008 | Google.Scholar | Google Scholar |
| Turkish | 2007 | Google.Scholar | Google Scholar |
| Turkish | 2006 | Google.Scholar | Google Scholar |
| Turkish | 2005 | Google.Scholar | Google Scholar |
| Turkish | 2004 | Google.Scholar | Google Scholar |
| Turkish | 2003 | Google.Scholar | Google Scholar |
| Turkish | 2002 | Google.Scholar | Google Scholar |
| Turkish | 2001 | Google.Scholar | Google Scholar |
| Turkish | 2000 | Google.Scholar | Google Scholar |
| Turkish | 1999 | Google.Scholar | Google Scholar |
| Turkish | 1998 | Google.Scholar | Google Scholar |
| Turkish | 1997 | Google.Scholar | Google Scholar |
| Turkish | 1996 | Google.Scholar | Google Scholar |
| Turkish | 1995 | Google.Scholar | Google Scholar |
| Turkish | 1994 | Google.Scholar | Google Scholar |
| Turkish | 1993 | Google.Scholar | Google Scholar |
| Turkish | 1992 | Google.Scholar | Google Scholar |
| Turkish | 1991 | Google.Scholar | Google Scholar |
| Turkish | 1990 | Google.Scholar | Google Scholar |
| Turkish | 1989 | Google.Scholar | Google Scholar |
| Turkish | 1988 | Google.Scholar | Google Scholar |
| Turkish | 1987 | Google.Scholar | Google Scholar |
| Turkish | 1986 | Google.Scholar | Google Scholar |
| Turkish | 1985 | Google.Scholar | Google Scholar |
| Turkish | 1984 | Google.Scholar | Google Scholar |
| Turkish | 1983 | Google.Scholar | Google Scholar |
| Turkish | 1982 | Google.Scholar | Google Scholar |
| Turkish | 1981 | Google.Scholar | Google Scholar |
| Turkish | 1980 | Google.Scholar | Google Scholar |
| Turkish | 2018 | Scopus | Scopus |
| Turkish | 2017 | Scopus | Scopus |
| Turkish | 2016 | Scopus | Scopus |
| Turkish | 2015 | Scopus | Scopus |
| Turkish | 2014 | Scopus | Scopus |
| Turkish | 2013 | Scopus | Scopus |
| Turkish | 2012 | Scopus | Scopus |
| Turkish | 2011 | Scopus | Scopus |
| Turkish | 2010 | Scopus | Scopus |
| Turkish | 2009 | Scopus | Scopus |
| Turkish | 2008 | Scopus | Scopus |
| Turkish | 2007 | Scopus | Scopus |
| Turkish | 2006 | Scopus | Scopus |
| Turkish | 2005 | Scopus | Scopus |
| Turkish | 2004 | Scopus | Scopus |
| Turkish | 2003 | Scopus | Scopus |
| Turkish | 2002 | Scopus | Scopus |
| Turkish | 2001 | Scopus | Scopus |
| Turkish | 2000 | Scopus | Scopus |
| Turkish | 1999 | Scopus | Scopus |
| Turkish | 1998 | Scopus | Scopus |
| Turkish | 1997 | Scopus | Scopus |
| Turkish | 1996 | Scopus | Scopus |
| Turkish | 1995 | Scopus | Scopus |
| Turkish | 1994 | Scopus | Scopus |
| Turkish | 1993 | Scopus | Scopus |
| Turkish | 1992 | Scopus | Scopus |
| Turkish | 1991 | Scopus | Scopus |
| Turkish | 1990 | Scopus | Scopus |
| Turkish | 1989 | Scopus | Scopus |
| Turkish | 1988 | Scopus | Scopus |
| Turkish | 1987 | Scopus | Scopus |
| Turkish | 1986 | Scopus | Scopus |
| Turkish | 1985 | Scopus | Scopus |
| Turkish | 1984 | Scopus | Scopus |
| Turkish | 1983 | Scopus | Scopus |
| Turkish | 1982 | Scopus | Scopus |
| Turkish | 1981 | Scopus | Scopus |
| Turkish | 1980 | Scopus | Scopus |
| Turkish | 2018 | Web.of.Science | Web of Science |
| Turkish | 2017 | Web.of.Science | Web of Science |
| Turkish | 2016 | Web.of.Science | Web of Science |
| Turkish | 2015 | Web.of.Science | Web of Science |
| Turkish | 2014 | Web.of.Science | Web of Science |
| Turkish | 2013 | Web.of.Science | Web of Science |
| Turkish | 2012 | Web.of.Science | Web of Science |
| Turkish | 2011 | Web.of.Science | Web of Science |
| Turkish | 2010 | Web.of.Science | Web of Science |
| Turkish | 2009 | Web.of.Science | Web of Science |
| Turkish | 2008 | Web.of.Science | Web of Science |
| Turkish | 2007 | Web.of.Science | Web of Science |
| Turkish | 2006 | Web.of.Science | Web of Science |
| Turkish | 2005 | Web.of.Science | Web of Science |
| Turkish | 2004 | Web.of.Science | Web of Science |
| Turkish | 2003 | Web.of.Science | Web of Science |
| Turkish | 2002 | Web.of.Science | Web of Science |
| Turkish | 2001 | Web.of.Science | Web of Science |
| Turkish | 2000 | Web.of.Science | Web of Science |
| Turkish | 1999 | Web.of.Science | Web of Science |
| Turkish | 1998 | Web.of.Science | Web of Science |
| Turkish | 1997 | Web.of.Science | Web of Science |
| Turkish | 1996 | Web.of.Science | Web of Science |
| Turkish | 1995 | Web.of.Science | Web of Science |
| Turkish | 1994 | Web.of.Science | Web of Science |
| Turkish | 1993 | Web.of.Science | Web of Science |
| Turkish | 1992 | Web.of.Science | Web of Science |
| Turkish | 1991 | Web.of.Science | Web of Science |
| Turkish | 1990 | Web.of.Science | Web of Science |
| Turkish | 1989 | Web.of.Science | Web of Science |
| Turkish | 1988 | Web.of.Science | Web of Science |
| Turkish | 1987 | Web.of.Science | Web of Science |
| Turkish | 1986 | Web.of.Science | Web of Science |
| Turkish | 1985 | Web.of.Science | Web of Science |
| Turkish | 1984 | Web.of.Science | Web of Science |
| Turkish | 1983 | Web.of.Science | Web of Science |
| Turkish | 1982 | Web.of.Science | Web of Science |
| Turkish | 1981 | Web.of.Science | Web of Science |
| Turkish | 1980 | Web.of.Science | Web of Science |

**Appendix VI**

**Abstracts in non-English languages**

**Swedish**

**Abstract:** Idag är engelska vida erkänt som vetenskapens språk, och vetenskapliga publikationer på engelska ökar i hög takt. Det antas ofta att antalet publikationer på andra språk samtidigt minskar. Detta antagande bidrar till en underanvändning av publikationer på andra språk inom naturvård, speciellt inom den internationella litteraturen. Dock är dokumentationen knapphändig gällande antalet artiklar inom naturvård på olika språk. Vi använde både lokala och internationella söksystem för att hitta vetenskapliga artiklar inom naturvård som publicerats mellan 1980 och 2018 på engelska och 15 andra språk. Dessutom jämförde vi ökningstakten mellan olika språk. Vi fann att antalet artiklar som publiceras årligen ökade signifikant under de senaste 39 åren för minst 12 av de 15 undersökta språken i en takt liknande den för artiklar på engelska. De kontrasterande resultaten för de resterande tre språken berodde på använda söksystem. Artiklar inom naturvård började dyka upp i början av 1980-talet och har ökat exponentiellt sedan 1990-talet för de flesta av språken. Vi observerade också en markant variation gällande hur många artiklar på andra språk än engelska de olika söksystemen identifierade. Google Scholar identifierade flest artiklar för 11 och lokala söksystem för 4 språk som inte var engelska. Dock var andelen referentgranskade relevanta artiklar på andra språk än engelska högst med Scopus, följt av Web of Science och lokala söksystem, och lägst med Google Scholar. Ungefär 20 % av de samplade artiklarna på andra språk än engelska hade ingen titel eller sammanfattning på engelska, och var därför i teorin ej sökbara med engelska nyckelord. Möjliga anledningar till detta är språkbarriärer och behovet att sprida forskning i länder där engelska inte används i stor utsträckning. Givet tidigare vetenskapliga belägg för att statistiska resultat och studiekaraktärer skiljer sig åt mellan artiklar på engelska och andra språk, kommer artiklar på andra språk att fortsätta spela en viktig roll i att öka vår förståelse för biologisk mångfald och dess bevarande.

**German**

**Zusammenfassung:** Englisch ist heutzutage weithin als die Sprache der Wissenschaft anerkannt und die Zahl der englischsprachigen Veröffentlichungen nimmt rapide zu. Oft wird angenommen, dass die Zahl der nicht-englischsprachigen wissenschaftlichen Veröffentlichungen abnimmt. Diese Annahme trägt dazu bei, dass nicht-englischsprachige Veröffentlichungen in der Naturschutzwissenschaft, -praxis und -politik, insbesondere auf internationaler Ebene, zu wenig genutzt werden. Die Anzahl der in verschiedenen Sprachen veröffentlichten Naturschutzartikel ist jedoch nur unzureichend dokumentiert. Wir haben wissenschaftliche Artikel zum Thema Biodiversitätsschutz, die zwischen 1980 und 2018 in Englisch und 15 nicht-englischen Sprachen veröffentlicht wurden, mit lokalen und internationalen Suchsystemen durchsucht. Außerdem haben wir die Wachstumsrate in den verschiedenen Sprachen verglichen. Wir fanden heraus, dass in mindestens 12 der 15 nicht-englischen Sprachen die Anzahl der Artikel über den Erhalt der Artenvielfalt, die jährlich veröffentlicht werden, in den letzten 39 Jahren deutlich zugenommen hat, und zwar mit einer ähnlichen Rate wie bei englischsprachigen Artikeln. In den anderen drei Sprachen waren die Ergebnisse je nach Suchsystem unterschiedlich. Artikel zum Thema Naturschutz erschienen erstmals in den frühen 1980er Jahren und haben seit den 1990er Jahren in den meisten Sprachen exponentiell zugenommen. Auch bei der Anzahl der nicht-englischsprachigen Artikel, die in den verschiedenen Suchsystemen gefunden wurden, gab es deutliche Unterschiede. Google Scholar und lokale Literatursuchsysteme ermittelten die meisten Artikel für 11 bzw. vier nicht-englische Sprachen. Der Anteil relevanter Artikel mit Peer-Review, die in nicht-englischen Sprachen veröffentlicht wurden, war jedoch bei Scopus am höchsten, gefolgt von Web of Science und lokalen Suchsystemen, und am niedrigsten bei Google Scholar. Etwa 20 % der in die Stichprobe aufgenommenen nicht-englischsprachigen Artikel enthielten weder Titel noch Zusammenfassung in englischer Sprache und sind daher theoretisch nicht über englische Schlüsselwörter recherchierbar. Mögliche Gründe hierfür sind Sprachbarrieren und die Notwendigkeit, Forschungsergebnisse in Ländern zu verbreiten, in denen Englisch nicht weit verbreitet ist. Angesichts der bekannten Verzerrungen in den statistischen Ergebnissen und Studienmerkmalen zwischen englischsprachigen und nicht-englischsprachigen Studien werden nicht-englischsprachige Artikel weiterhin eine wichtige Rolle bei der Verbesserung unseres Verständnisses der biologischen Vielfalt und ihrer Erhaltung spielen.

**Dutch**

**Samenvatting.** Tegenwoordig wordt Engels algemeen erkend als de taal van de wetenschap, en Engelstalige publicaties nemen ook snel toe. Vaak wordt aangenomen dat het aantal niet-Engelstalige wetenschappelijke publicaties afneemt. Deze veronderstelling draagt bij aan de onderbenutting van niet-Engelstalige wetenschappelijke publicaties ten aanzien van bescherming, toepassingen en beleid, vooral op internationaal niveau. Het aantal wetenschappelijke artikelen dat in verschillende talen wordt gepubliceerd ten aanzien van bescherming en biodiversiteit, is echter slecht gedocumenteerd. We zochten naar wetenschappelijke artikelen over bescherming en biodiversiteit gepubliceerd tussen 1980 en 2018 in het Engels en 15 niet-Engelse talen met behulp van zowel lokale als internationale zoeksystemen. Verder vergeleken we de groeisnelheid in verschillende talen. We ontdekten dat in ten minste 12 van de 15 niet-Engelstalige artikelen over bescherming die jaarlijks worden gepubliceerd, de afgelopen 39 jaar aanzienlijk is toegenomen, met een snelheid die vergelijkbaar is met Engelstalige artikelen. De andere drie talen vertoonden contrasterende resultaten, afhankelijk van het zoeksysteem. Beschermingsartikelen begonnen te verschijnen in de vroege jaren 1980 en zijn exponentieel toegenomen sinds de jaren 1990 voor de meeste talen. We zagen ook een duidelijke variatie in het aantal niet-Engelstalige artikelen dat onder de zoeksystemen werd geïdentificeerd. Google Scholar en lokale literatuurzoeksystemen identificeerden de meeste artikelen voor respectievelijk 11 en vier niet-Engelse talen. Het aandeel ‘peer-reviewed’ artikelen in niet-Engelse talen was echter het hoogst in Scopus, gevolgd door Web of Science en lokale zoeksystemen, en het laagst in Google Scholar. Ongeveer 20% van de onderzochte niet-Engelstalige artikelen had geen titel of samenvatting in het Engels en is dus in theorie niet doorzoekbaar met behulp van Engelse trefwoorden. Mogelijke redenen hiervoor zijn taalbarrières en de noodzaak om onderzoek te verspreiden in landen waar Engels niet veel wordt gesproken. Aangezien er een afwijking in statistische resultaten en eigenschappen tussen Engelstalige en niet-Engelstalige studies bestaat, zullen niet-Engelstalige artikelen een belangrijke rol blijven spelen bij het verbeteren van ons begrip van biodiversiteit en de bescherming ervan.

**Italian**

**Riassunto:** Oggi l'inglese è ampiamente riconosciuto come la lingua della scienza. Mentre le pubblicazioni scientifiche in lingua inglese sono in rapido aumento, si presume spesso che quelle non in lingua inglese stiano diminuendo. Questa supposizione contribuisce al minore utilizzo delle pubblicazioni non in lingua inglese negli studi e nelle pratiche legate alla conservazione della biodiversità, soprattutto a livello internazionale. Tuttavia, il numero di articoli sulla conservazione pubblicati in lingue diverse dall’inglese è scarsamente documentato. In questo lavoro, abbiamo cercato articoli scientifici sulla conservazione della biodiversità pubblicati tra il 1980 e il 2018 in inglese e in altre 15 lingue, utilizzando motori di ricerca locali e internazionali. Inoltre, abbiamo confrontato il tasso di crescita degli articoli tra lingue diverse. Il numero di articoli sulla conservazione pubblicati annualmente in 12 lingue è aumentato significativamente negli ultimi 39 anni, a un ritmo simile agli articoli in lingua inglese. Le altre tre lingue hanno mostrato risultati contrastanti a seconda del motore di ricerca utilizzato. Gli articoli sulla conservazione hanno iniziato ad apparire nella letteratura all'inizio degli anni '80 e sono aumentati per la maggior parte delle lingue in modo esponenziale dagli anni '90. Abbiamo anche osservato una marcata variazione nel numero di articoli non in lingua inglese identificati tra i sistemi di ricerca. Google Scholar e i sistemi di ricerca locale hanno identificato la maggior parte degli articoli rispettivamente per 11 e quattro lingue diverse dall'inglese. Tuttavia, la percentuale di articoli pertinenti sottoposti a revisione paritaria pubblicati in lingue diverse dall'inglese era più alta in Scopus, seguita da Web of Science e dai sistemi di ricerca locali e più bassa in Google Scholar. Circa il 20% degli articoli non in lingua inglese campionati non aveva alcun titolo o riassunto in inglese e risultava quindi, almeno in teoria, non ricercabile utilizzando parole chiave inglesi. Possibili ragioni di ciò includono barriere linguistiche e la necessità di diffondere la ricerca in paesi in cui l'inglese non è ampiamente parlato. Considerando la nota differenza tra i risultati statistici e le caratteristiche degli studi pubblicati in lingua inglese o in altre lingue, gli articoli in lingua non inglese continueranno a svolgere un ruolo importante nel migliorare la nostra comprensione della biodiversità e della sua conservazione.

**Russian**

**Abstract:** На сегодняшний день английский язык признан языком науки во всем мире, и количество публикаций на английском языке стремительно растет. Часто предполагается, что количество научных публикаций не на английском языке сокращается. Это предположение способствует недостаточному использованию неанглоязычных публикаций в области природоохранной науки, практики и политики, особенно на международном уровне. Однако количество статей об сохранении биоразнообразия и охране природы в целом, опубликованных на разных языках, плохо документировано. Нами был проведен поиск научных статей по теме сохранения биоразнообразия, опубликованных в период с 1980 по 2018 год на английском языке и на 15 других языках, с помощью как локальных, так и международных поисковых систем. Далее было проведено сравнение темпов роста количества статей на разных языках. Было обнаружено, что по крайней мере в 12 из 15 неанглоязычных статей по теме сохранения биоразнообразия, публикуемых ежегодно, за последние 39 лет значительно увеличилось количество статей, со скоростью, аналогичной статьям на английском языке. Остальные три языка показали противоположные результаты в зависимости от поисковой системы. Статьи о сохранении биоразнообразия начали появляться в начале 1980-х годов и с 1990-х годов увеличились в геометрической прогрессии для большинства языков. Были также отмечены заметные различия в количестве неанглоязычных статей, выявленных в поисковых системах. Система поиска Google Scholar и локальные системы поиска литературы выявили наибольшее количество статей для 11 из четырех неанглийских языков соответственно. Однако доля рецензируемых соответствующих статей, опубликованных не на английском языке, была самой высокой в Scopus, за которой следовали Web of Science и локальные поисковые системы, и самой низкой в Google Scholar. Около 20% отобранных неанглоязычных статей не содержали названия или аннотации на английском языке и, таким образом, теоретически не могут быть найдены с использованием английских ключевых слов. Возможные причины этого включают языковые барьеры и необходимость распространения исследований в странах, где английский язык не распространен. Учитывая известную предвзятость в статистических результатах и характеристиках исследований между исследованиями на английском и других языках, статьи на языке, отличном от английского, будут продолжать играть важную роль в улучшении нашего понимания биоразнообразия и его сохранения.

**Korean**

초록 : 오늘날, 영어는 과학의 언어로서 널리 인식되고 있으며, 영어 출판물들은 빠르게 증가하고 있다. 따라서 비영어 과학 출판물들은 줄어들고 있다고 주로 가정된다. 이러한 가정은 특히 국제적 수준에서 보존에 관한 과학, 실무, 그리고 정책들에 대한 비영어 언어 출판물에 대한 불충분한 활용의 원인이 된다. 그러나, 영어 이외의 언어로 출판된 보존 관련 논문의 수는 제대로 기록되지 않았다. 우리는 1980년에서 2018년 사이에 영어 및 15개의 비영어 언어로 출판된 생물 다양성 보존에 관한 과학 논문들을 지역 및 국제 검색 시스템을 사용하여 검색했다. 또한, 각 언어별 논문의 증가율을 비교했다. 지난 39년 동안 연간 출판된 보존에 관하여 15개의 비영어 언어로 출판된 논문들 중에서 최소 12개의 비영어 언어의 논문이 영어 논문과 비슷한 속도로 크게 증가한 것으로 나타났다. 나머지 3개의 비영어 언어로 출판된 보존에 관한 논문은 검색 시스템에 따라 상반된 결과를 보였다. 보존에 관한 논문은 1980년대 초에 나타나기 시작했고 1990년대 이후 대부분의 언어에서 기하급수적으로 증가했다. 또한, 검색 시스템에 따라 식별된 비영어 언어 논문의 수는 현저하게 달랐다. Google Scholar와 지역 문헌 검색 시스템은 각각 11개와 4개의 비영어 언어에서 논문을 가장 많이 식별했다. 그러나, 비영어 언어로 출판된 동료 평가 논문의 비율은 Scopus에서 가장 높았고, Web of Science와 지역 검색 시스템이 그 뒤를 이었으며, Google Scholar에서 가장 낮았다. 표본으로 추출된 비영어 언어 논문의 약 20%는 영어로 된 제목이나 초록을 제공하지 않았기 때문에 이론적으로는 영어 키워드를 사용하여 검색할 수 없었다. 그 이유로는 언어의 장벽이 존재한다는 점을 생각할 수 있어, 영어가 별로 이용되지 않는 나라에서 연구 보급의 필요성이 요구된다. 영어와 비영어 연구 사이의 통계적 결과와 연구 특성에 대해 알려진 편향을 고려할 때, 앞으로도 비영어 언어 논문은 생물 다양성과 보존에 대한 우리의 이해를 향상시키는 데 중요한 역할을 할 것이다.

**Japanese**

　現在、英語は科学に用いる言語として広く認知されており、英語を用いた出版物は急速に増加している。そのため英語以外の言語を用いた科学に関する出版物は減少していると考えられる。この仮定は特に国際レベルでの保全科学の実践と政策に英語以外の言語で作成された出版物が十分に利用されない一因となる。しかし英語以外の言語を用いて出版された保全に関する出版物の数はこれまでほとんど記録されていない。そこで我々は1980年から2018年の間に英語と15の非英語言語において出版された生物多様性保全に関する科学論文を、国内と海外の検索システムを用いて検索し、異なる言語間での出版物の増加率を比較した。その結果、過去39年間で年毎に出版された保全に関する論文数は、英語以外の15言語の論文のうち少なくとも12言語において、英語論文と同様の割合で著しく増加していることが明らかとなった。また他の3言語では、検索システムによって対照的な結果となった。保全に関する論文は1980年代前半に出現し始め、ほとんどの言語において1990年代以降に急激に増加していた。また英語以外の言語を用いて検出された論文の数は、利用する検索システムによって差が顕著であることが確認された。Google Scholarと現地の文献検索システムにおいて、英語以外の言語で書かれた論文が最も多く検出されたのはそれぞれ11言語と4言語となった。しかし、英語以外の言語で出版された査読付きの保全に関する論文の割合はScopusが最も多く、次いでWeb of science、現地の文献検索システム、そしてGoogle Scholar が最も少なくなった。また英語以外の言語で書かれた論文の約20％は英語で書かれた題名や抄録が無いため、理論上英語のキーワードを用いて検索を行うことができなかった。この理由として、言語の壁が存在することが考えられ、英語があまり利用されていない国で研究普及の必要性が求められる。英語で書かれた論文と英語以外の言語で書かれた論文では統計結果や研究特性に偏りがあることも知られているため、英語以外の言語で書かれた論文は今後も生物多様性とその保全に関する我々の理解を深めるために重要な役割を果たすと考えられる。

**Turkish**

**Öz:** Günümüzde İngilizce yaygın olarak bilim dili olarak kabul edilirken, İngilizce yayınlar hızla artmaktadır. Genellikle İngilizce olmayan bilimsel yayınların sayısının azaldığı varsayılmaktadır. Bu varsayım, özellikle uluslararası düzeyde koruma biyolojisi bilimi, uygulamaları ve politikalarında İngilizce olmayan yayınların yetersiz kullanımına neden olmaktadır. Ancak, farklı dillerde yayınlanan koruma makalelerinin sayısı yetersiz sayıdadır. Hem yerel hem de uluslararası arama sistemlerini kullanarak 1980 ve 2018 yılları arasında İngilizce ve İngilizce olmayan 15 dilde yayınlanan biyolojik çeşitliliğin korunmasına ilişkin bilimsel makaleleri taradık. Ayrıca, farklı dillerdeki büyüme oranını karşılaştırdık. Her yıl yayınlanan koruma biyolojisi konusunda İngilizce olmayan 15 makalenin en az 12'sinde, İngilizce makalelere benzer bir oranda son 39 yılda önemli ölçüde yayınlarda artış olduğunu bulduk. Diğer üç dil, arama sistemine bağlı olarak zıt sonuçlar gösterdi. Koruma biyolojisi alanındaki makaleler 1980 yılların başında görünmeye başlarken ve çoğu dil için yayınlar 1990'lardan beri katlanarak arttı. Ayrıca, arama sistemleri arasında İngilizce olmayan makalelerin sayısında da belirgin bir farklılık gözlemledik. Google Akademik ve yerel literatür arama sistemleri, en çok makaleyi sırasıyla 11 ve İngilizce olmayan dört dil için belirledi. Ancak, İngilizce olmayan dillerde yayınlanan hakemli dergilerdeki makalelerin oranı en yüksek Scopus'ta gözlemlenirken, onu Web of Science ve yerel arama sistemleri ve en düşük Google Akademik izledi. Örneklenen İngilizce olmayan makalelerin yaklaşık %20'si İngilizce başlık veya özet sunmadığı için teorik olarak İngilizce anahtar kelimeler kullanılarak aranamamaktadır. Bunun olası nedenleri arasında dil engelleri ve İngilizce’nin yaygın olarak konuşulmadığı ülkelerde araştırmaları yaygınlaştırma ihtiyacı sayılabilir. İngilizce ve İngilizce olmayan çalışmalar arasındaki istatistiksel sonuçlar ve çalışma özelliklerindeki bilinen önyargı göz önüne alındığında, İngilizce olmayan makaleler, biyolojik çeşitlilik ve korunması konusundaki anlayışımızı geliştirmede önemli bir rol oynamaya devam edecektir.

**French / Français**

**Résumé :**

L'anglais est largement reconnu comme la langue internationale des sciences, et les publications en langue anglaise augmentent rapidement. On suppose souvent que le nombre de publications scientifiques non-anglophones est en baisse. Cette hypothèse contribue à la sous-utilisation des publications non-anglophones dans la science, les pratiques et les politiques de conservation de la biodiversité, en particulier au niveau international. Cependant, le nombre d'articles liés à la conservation publiés dans différentes langues est peu documenté. Nous avons identifié les articles scientifiques sur la conservation de la biodiversité publiés entre 1980 et 2018 en anglais et dans 15 autres langues en utilisant des systèmes de recherche locaux et internationaux. Nous avons également comparé les taux de croissance des publications entre les différentes langues. Dans au moins 12 des 15 langues non-anglophones, les articles sur la conservation de la biodiversité publiés annuellement ont augmenté de manière significative au cours des 39 dernières années, à un taux similaire à celui des articles publiés en langue anglaise. Les trois autres langues ont montré des résultats contrastés en fonction du système de recherche. Les articles sur la conservation ont commencé à apparaître au début des années 1980 et ont augmenté de manière exponentielle depuis les années 1990 pour la plupart des langues. Nous avons observé une variation marquée du nombre d'articles non-anglophones identifiés selon les systèmes de recherche. Google Scholar et les systèmes de recherche locaux de littérature ont identifié le plus grand nombre d'articles pour onze et quatre langues non-anglophones, respectivement. Toutefois, la proportion d'articles pertinents, évalués par des pairs et publiés dans des langues autres que l'anglais était la plus élevée sur Scopus, suivi de Web of Science et des systèmes de recherche locaux, et la plus faible sur Google Scholar. Environ 20 % des articles publiés dans une langue non-anglophone n'ont pas de titre ou de résumé en anglais et ne peuvent donc pas, en théorie, être recherchés à l'aide de mots-clés écrits en anglais. Les raisons possibles pouvant expliquer ce résultat incluent les barrières linguistiques et la nécessité de diffuser les recherches dans les pays où l'anglais n'est pas largement parlé. Étant donné le biais connu entre les résultats statistiques et les caractéristiques des études publiées en anglais et celles publiées en d’autres langues, les articles non-anglophones continueront à jouer un rôle important dans l'amélioration de notre compréhension de la biodiversité et de sa conservation.

**Polish**

**Abstrakt:** W dzisiejszych czasach język angielski jest powszechnie uznawany za język nauki, a liczba publikacji wydawanych w tym języku szybko rośnie. Często przyjmuje się również że liczba nieanglojęzycznych publikacji naukowych maleje. To założenie przyczynia się do niedostatecznego wykorzystywania publikacji nieanglojęzycznych zarówno w naukowych, jak i praktycznych aspektach ochrony przyrody, zwłaszcza na poziomie międzynarodowym. Jednak liczba artykułów dotyczących ochrony przyrody publikowanych w różnych językach jest słabo udokumentowana. Przeszukaliśmy artykuły naukowe na temat ochrony bioróżnorodności opublikowane w latach 1980-2018 w języku angielskim i 15 językach innych niż angielski, korzystając z lokalnych i międzynarodowych systemów wyszukiwania. Ponadto porównaliśmy tempo wzrostu pojawiania się takich artykułów w różnych językach. Odkryliśmy, że co najmniej 12 z 15 badanych języków liczba artykułów na temat ochrony przyrody znacznie wzrosła w ciągu ostatnich 39 lat, w tempie podobnym do artykułów anglojęzycznych. Pozostałe trzy języki wykazały kontrastujące wyniki w zależności od systemu wyszukiwania. Artykuły z zakresu ochrony przyrody zaczęły pojawiać się na początku lat 80. i w większości języków rosły wykładniczo od lat 90. XX wieku. Zaobserwowaliśmy również wyraźne zróżnicowanie liczby artykułów nieanglojęzycznych zidentyfikowanych w systemach wyszukiwania. Google Scholar i systemy wyszukiwania literatury lokalnej zidentyfikowały najwięcej artykułów odpowiednio dla jedenastu i czterech języków innych niż angielski. Jednak odsetek recenzowanych artykułów opublikowanych w językach innych niż angielski był najwyższy w Scopus, następnie w Web of Science i lokalnych systemach wyszukiwania, a najniższy w Google Scholar. Około 20% artykułów nieanglojęzycznych objętych próbą nie zawiera tytułu ani streszczenia w języku angielskim, a zatem teoretycznie nie można ich przeszukiwać za pomocą angielskich słów kluczowych. Możliwe przyczyny tego zjawiska to bariery językowe i potrzeba rozpowszechniania badań w krajach, w których angielski nie jest powszechnie używany. Biorąc pod uwagę znane odchylenia w wynikach statystycznych i charakterystyce badań między badaniami anglojęzycznymi i nieanglojęzycznymi, artykuły nieanglojęzyczne będą nadal odgrywać ważną rolę w pogłębianiu naszego zrozumienia bioróżnorodności i jej ochrony.

**Portuguese**

**Resumo:** Hoje, o inglês é amplamente conhecido como o idioma da ciência, e o número de publicações em inglês está crescendo rapidamente. Frequentemente assume-se que o número de publicações em outros idiomas está diminuindo. Esse pressuposto contribui para uma subutilização de línguas diferentes da inglesa em publicações científicas, em práticas e em legislações relacionadas a conservação, especialmente em nível internacional. Porém, o número de artigos de conservação publicados em diferentes idiomas é ainda mal documentado. Realizamos uma busca por artigos científicos sobre conservação da biodiversidade publicados entre 1980 e 2018 em inglês e em outros 15 idiomas usando bases de dados bibliográficos locais e internacionais. Descobrimos que, em pelo menos 12 das 15 línguas não-inglesas pesquisadas, o número de artigos em conservação publicados anualmente aumentou significativamente nos últimos 39 anos, em uma taxa similar à de artigos em inglês. As outras três línguas mostraram resultados contrastantes dependendo da base de dados utilizada. Artigos em conservação da biodiversidade começaram a surgir no começo da década de 1980 e, desde então, cresceram exponencialmente em número desde os anos 1990 na maioria dos idiomas. Também observamos uma forte variação no número de artigos em língua não-inglesa entre diferentes bases de dados. O Google Scholar e as bases bibliográficas locais retornaram a maioria dos artigos para onze e quatro idiomas não-ingleses, respectivamente. No entanto, a proporção de artigos relevantes revisados por pares publicados em língua não-inglesa foi maior no Scopus, seguido por Web of Science e bases de dados locais, e menor no Google Scholar. Cerca de 20% dos artigos encontrados em idioma não-inglês não apresentavam título ou resumo em inglês, e portanto são, teoricamente, impossíveis de serem localizados usando apenas palavras-chave em inglês. Possíveis razões para isso incluem barreiras linguísticas e a necessidade de disseminar pesquisa em países onde o inglês não é amplamente usado. Apesar de haver vieses nos resultados estatísticos e nas características do estudo, artigos em língua não-inglesa continuarão desempenhando um papel importante em melhorar nosso conhecimento sobre biodiversidade e sua conservação.

**Persian/Farsi**

**چکیده:** امروزه زبان انگلیسی به طور گسترده ای به عنوان زبان علم شناخته می شود، چراکه انتشارات انگلیسی زبان به سرعت در حال افزایش هستند. اغلب تصور می شود که تعداد انتشارات علمی غیر انگلیسی زبان در حال کاهش است. این فرض منجر به کمرنگ شدن نقش مقالات غیر انگلیسی زبان در تولید علم حفاظت و همچنین طراحی شیوه‌ها و سیاست گذاری های حفاظتی، به ویژه در سطح بین المللی شده است. این در حالیست که مستندات موجود از تعداد مقالات حفاظتی منتشر شده به زبان های مختلف بسیار ضعیف است. در این مطالعه ما با استفاده از سیستم‌های جستجوی محلی و بین‌المللی به جستجوی مقالات علمی مربوط به حفاظت از تنوع زیستی که بین سال‌های ۱۹۸۰ و ۲۰۱۸ به زبان انگلیسی و ۱۵ زبان غیرانگلیسی منتشر شده پرداختیم. ما همچنین نرخ رشد تعداد مقالات را در زبان‌های مختلف مقایسه کردیم. ما دریافتیم که حداقل در ۱۲ زبان از ۱۵ زبان غیرانگلیسی مورد بررسی، تعداد مقالات مربوط به حفاظت در طی ۳۹ سال گذشته افزایش قابل توجهی داشته است، با نرخی مشابه مقالات انگلیسی زبان. سه زبان دیگر بسته به سیستم جستجو نتایج متضادی نشان دادند. مقالات حفاظتی در اوایل دهه ۱۹۸۰ ظاهر شدند و از دهه ۱۹۹۰ برای بیشتر زبان ها به طور تصاعدی افزایش یافتند. ما همچنین تنوع قابل توجهی در تعداد مقالات غیر انگلیسی زبان شناسایی شده در میان سیستم های جستجو مشاهده کردیم. Google Scholar و سیستم‌های جستجوی محلی بیشترین مقالات را به ترتیب برای ۱۱ و چهار زبان غیرانگلیسی شناسایی کردند. بالاترین نسبت مقالات مرتبط و داوری شده غیر انگلیسی زبان‌ در Scopus و در رده های بعدی در Web of Science ،سیستم‌های جستجوی محلی و Google Scholar یافت شد. حدود ۲۰ درصد از نمونه‌ای از مقالات غیرانگلیسی زبان عنوان یا چکیده ای به زبان انگلیسی ارائه نکرده بودند و بنابراین با استفاده از کلمات کلیدی انگلیسی قابل جستجو نیستند. دلایل احتمالی این امر شامل موانع زبانی و نیاز به انتشار تحقیقات در کشورهایی است که انگلیسی به طور گسترده صحبت نمی شود. با توجه به تفاوت نتایج آماری و ویژگی های مطالعاتی بین مطالعات انگلیسی و غیرانگلیسی زبان، مقالات غیرانگلیسی زبان همچنان نقش مهمی در بهبود درک ما از تنوع زیستی و حفاظت از آن
